# Isoprenoid-phospholipid conjugates as potential therapeutic agents: Synthesis, characterization and antiproliferative studies

**DOI:** 10.1371/journal.pone.0172238

**Published:** 2017-02-14

**Authors:** Anna Gliszczyńska, Natalia Niezgoda, Witold Gładkowski, Marta Świtalska, Joanna Wietrzyk

**Affiliations:** 1 Department of Chemistry, Wrocław University of Environmental and Life Sciences, Wrocław, Poland; 2 Ludwik Hirszfeld Institute of Immunology and Experimental Therapy, Polish Academy of Sciences, Department of Experimental Oncology, Wrocław, Poland; Helsingin Yliopisto, FINLAND

## Abstract

The aim of this research was to extend application field of isoprenoid compounds by their introduction into phospholipid structure as the transport vehicle. The series of novel isoprenoid phospholipids were synthesized in high yields (24–97%), their structures were fully characterized and its anticancer activity was investigated *in vitro* towards several cell lines of different origin. Most of synthesized compounds showed a significantly higher antiproliferative effect on tested cell lines than free terpene acids. The most active phosphatidylcholine analogue, containing 2,3-dihydro-3-vinylfarnesoic acids instead of fatty acids in both *sn*-1 and *sn-*2 position, inhibits the proliferation of colon cancer cells at 13.6 μM.

## Introduction

In recent years, numerous epidemiological studies about the correlation between consumption of fruits, vegetables and other plant products and reduction of cancer incidence have been presented [1,2]. In the group of bioactive constituents of plants, isoprenoids are mentioned as one of the most important cancer-protective molecules [3]. They represent a broad class of mevalonate-derived phytochemicals, which exhibit many pharmacological and chemopreventive effects. They influence on the life cycle of the cells on the molecular level, activation of certain genes and apoptosis of tumor cells, what is the subject of extensive studies [4,5]. The results of the research from this area show that isoprenoid compounds inhibit proliferation of cancer cell lines, causing inhibition of cell division and transition of cells into the phase of programmed cell death. Limonen has documented antitumor activity against rodent mammary, liver, lung, stomach and skin cancers [6–9]. Chemopreventive activity of perillyl alcohol was also demonstrated against pancreatic [10] and prostate cancer cell lines [11]. For that compound even several clinical trials, including phase I and II trials have been recently conducted [12,13]. It was confirmed that geraniol has also high cytotoxic activity *in vitro* and *in vivo* against murine leukemia, hepatoma and melanoma cells [14,15], similar properties have been documented for farnesol [14, 16, 3].

Replacement of fatty acids in phospholipids with therapeutic or desirable fatty acids improves the physico-chemical, nutritional and pharmaceutical functions. Phosphatidylcholine is particularly attractive lipid for this type of modification because of its health-promoting activity. It is a source of biologically active choline, which is a substrate in the synthesis of acetylcholine strictly responsible for many vital functions of organism such as breathing, heart rate or memory processes [17,18]. Phospholipids with defined molecular structure have been used in drug delivery and gene therapy [19–21].

Therefore it was of interest to synthesize the phospholipid molecules having isoprene acid as the acyl residue in the phospholipid backbone and to combine the valuable activities of these two groups of compounds. In our previous paper we have reported on the synthesis of phosphatidylcholine analogues containing geranic and citronellic acids in the *sn-1* and *sn-2* position [22] and present the results of their cytotoxic studies on leukemia, lung, breast, liver, colon and deoxorubicin-resistant colon cancer cell lines [22]. Encouraged by these results in the current paper we extended our earlier studies, preparing novel isoprenoid-phospholipid conjugates with several new isoprenoid acids. We report on their synthesis, characterization and anticancer activity trying to find relationship between some structural feature, i.e. the number of unsaturated bonds, the chain length, the cyclic or acyclic structure of isoprenoids and their antitumor activities. The synthesized molecules could be potential prodrugs or diet supplements.

## Materials and methods

### General

HPLC chromatography was performed on an Ultimate 3000 Dionex chromatograph equipped with a DGP-3600A dual-pump fluid control module, a TCC-3200 thermostatted column compartment, and a WPS-3000 autosampler. A Corona charged aerosol detector (CAD) from ESA Biosciences was used, with the following parameters: acquisition range: 100 pA, digital filter set to none, N_2_ pressure: 0.24 MPa. The system and data acquisition were carried out using the *Chromeleon 6*.*80* software (Dionex Corporation). Analysis was carried out using a Betasil DIOL 5-mm column (Thermo, 150 2.1mm). The injection volume was 15 mL in all of the experiments and the cooling temperature for the samples was 20°C. The column temperature was maintained at 30°C. The total time of analysis was 19 min. The mobile phase had a constant flow of 1.5 mL min^-1^. Solvent A (1% HCOOH, 0.1% triethylamine (TEA) in water), solvent B (hexane), and solvent C (propan-2-ol) were used in gradient mode starting with 3:40:57 (A:B:C (vol-%/ vol-%/vol-%)), at 4 min 10:40:50, at 9 min 10:40:50, at 9.1 min 3:40:57 and at 19 min 43:40:57.

Column chromatography was performed on silica gel (Kieselgel 60, 230–400 mesh, Merck). Spectroscopic measurements were carried out on a Bruker Avance II 600 MHz spectrometer. Chemical shifts are given in ppm downfield from tetramethylsilane (TMS) as the internal standard. In ^31^P NMR spectra, chemical shifts were referenced to 85% H_3_PO_4_ as a standard. Coupling constant (*J*) values are given in Hertz. High-resolution mass spectra (HRMS) were recorded using electron spray ionization (ESI) technique on Waters ESI-Q-TOF Premier XE spectrometer.

### Substrates and chemicals for the synthesis

*sn*-Glycero-3-phosphocholine (GPC) in the enantiomerically pure form was purchased from Bachem and converted to the cadmium chloride complex (GPC **×** CdCl_2_) using the method described earlier [22]. Racemic acids: 3,7-dimethyl-3-vinyloct-6-enoic acid **(1)**, (*E*)-3,7,11-trimethyl-3-vinyldodeca-6,10-dienoic acid **(2)**, 2-(2-butylidene-1,3,3-trimethylcyclohexyl)-acetic acid **(3)** were obtained according to the procedure described before [23,24]. Organic solvents used in HPLC were purchased from Merck. Phospholipase A_2_ (Lecitase 10 L; 10,000 LEU/mL) was a gift from Novozymes.

### Chemical synthesis

Phytol (97% purity) was purchased from Aldrich and was used as a substrate for the synthesis of 3,7,11,15-tetramethyl-3-vinylhexadecanoic acid **(5)**. The detailed procedure for the synthesis of acid (**5**) was described ealier [23,24]. The spectroscopic data of obtained products is given below:

**Ethyl 3,7,11,15-tetramethyl-3-vinylhexadecanoate (4)**

oily liquid (86% yield); ^**1**^**H NMR** (600 MHz, CDCl_3_) δ: 0.83, 0.84 (two d, 6H, *J* = 5.4 Hz, CH_3_-20, CH_3_-21), 0.86 (d, 6H, *J* = 6.0 Hz, CH_3_-16, CH_3_-22), 1.05–1.37 (m, 20H, CH_2_-4, CH_2_-5, CH_2_-6, CH_2_-8, CH_2_-9, CH_2_-10, CH_2_-12, CH_2_-13, CH_2_-14, H-7, H-11,), 1.11 (s, 3H, CH_3_-19), 1.24 (t, 3H, *J* = 7.2 Hz, -O-CH_2_C**H**_**3**_), 1.52 (m, 1H, H-15), 2.30 (s, 2H, CH_2_-2), 4.1 (q, 2H, *J* = 7.2 Hz, -O-C**H**_**2**_CH_3_), 4.94 (d, 1H, *J* = 17.4 Hz, one of CH_2_-18), 5.00 (d, 1H, *J* = 10.8 Hz, one of CH_2_-18), 5.81 (dd, 1H, *J* = 10.8 and 17.4 Hz, H-17); ^**13**^**C NMR** (151 MHz, CDCl_3_) δ: 14.33 (-O-CH_2_**C**H_3_), 19.66, 19.70 (C-20, C-21), 22.65, 22.74 (C-16, C-22), 23.32 (CH_3_-19), 28.00 (C-15), 32.72, 32.81 (C-7, C-11), 21.52, 24.82, 24.83, 37.30, 37.41, 37.47, 37.65, 37.74, 39.39, 41.08 (C-3, C-4, C-5, C-6, C-8, C-9, C-10, C-12, C-13, C-14), 45.10 (C-2), 59.60 (-O-**C**H_2_CH_3_), 111.83 (C-18), 145.87 (C-17), 171.89 (C-1).

**3,7,11,15-Tetramethyl-3-vinylhexadecanoic acid (5)**

oily liquid (96% yield); ^**1**^**H NMR** (600 MHz, CDCl_3_) δ: 0.58, 0.60 (two d, 6H, *J* = 5.4 Hz, CH_3_-20, CH_3_-21), 0.62 (d, 6H, *J* = 6.6 Hz, CH_3_-16, CH_3_-22), 0.82–1.19 (m, 20H, CH_2_-4, CH_2_-5, CH_2_-6, CH_2_-8, CH_2_-9, CH_2_-10, CH_2_-12, CH_2_-13, CH_2_-14, H-7, H-11,), 0.89 (s, 3H, CH_3_-19), 1.28 (m, 1H, H-15), 2.05 (s, 2H, CH_2_-2), 4.70 (d, 1H, *J* = 17.4 Hz, one of CH_2_-18), 4.75 (d, 1H, *J* = 10.8 Hz, one of CH_2_-18), 5.58 (dd, 1H, *J* = 10.8 and 17.4 Hz, H-17); ^**13**^**C NMR** (151 MHz, CDCl_3_) δ: 19.63, 19.65 (C-20, C-21), 22.66, 22.75 (C-16, C-22), 23.23 (CH_3_-19), 28.01 (C-15), 32.72, 32.82 (C-7, C-11), 21.52, 24.47, 24.83, 37.30, 37.41, 37.46, 37.60, 37.70 39.39, 41.03 (C-3, C-4, C-5, C-6, C-8, C-9, C-10, C-12, C-13, C-14), 44.85 (C-2), 112.24 (C-18), 145.47 (C-17), 177.50 (C-1).

The series of new isoprenoid phospholipids **(6a-6d; 7a-7d; 8a-8d; 9a-9d)** were obtained according to the methods described earlier by Gliszczyńska et al. [22]. The yields of the reaction, their physical and spectroscopic data are presented below:

**1,2-diisoprenoyl-*sn*-glycero-3’-phosphatidylcholines**

**(2’*R*)-1’,2’-di[(3*R*)-3,7-dimethyl-3-vinylocta-6-enyl]-*sn*-glycero-3’-phosphocholine + (2’*R*)-1’,2’-di[(3*S*)-3,7-dimethyl-3-vinylocta-6-enyl]-*sn*-glycero-3’-phosphocholine (6a)**

mixture of two diasteroisomers, colourless greasy solid (97% yield, 99% purity (according to HPLC); ^**1**^**H NMR** (600 MHz, CDCl_3_/CD_3_OD 2:1 (v/v)) δ: 0.89, 0.90 (two s, 12H, CH_3_-11_sn-1_ (A), CH_3_-11_sn-1_ (B), CH_3_-11_sn-2_ (A), CH_3_-11_sn-2_ (B)), 1.14–1.22 (m, 8H, CH_2_-4_sn-1_ (A), CH_2_-4_sn-1_ (B), CH_2_-4_sn-2_ (A), CH_2_-4_sn-2_ (B)), 1.34, 1.43 (two s, 24 H, CH_3_-8_sn-1_ (A), CH_3_-8_sn-1_ (B), CH_3_-8_sn-2_ (A), CH_3_-8_sn-2_ (B), CH_3_-12_sn-1_ (A), CH_3_-12_sn-1_ (B), CH_3_-12_sn-2_ (A), CH_3_-12_sn-2_ (B)), 1.65–1.69 (m, 8H, CH_2_-5_sn-1_ (A), CH_2_-5_sn-1_ (B), CH_2_-5_sn-2_ (A), CH_2_-5_sn-2_ (B)), 2.08–2.15 (m, 8H, CH_2_-2_sn-1_ (A), CH_2_-2_sn-1_ (B), CH_2_-2_sn-2_ (A), CH_2_-2_sn-2_ (B)), 2.98 (s, 18H, -N(CH_3_)_3_ (A), -N(CH_3_)_3_ (B)), 3.37 (m, 4H, CH_2_-β (A), CH_2_-β (B), 3.71–3.74 (m, 4H, CH_2_-3' (A), CH_2_-3' (B)), 3.86–3.89 (two dd, *J* = 12.0, 6.6 Hz, 2H, one of CH_2_-1' (A), one of CH_2_-1' (B)), 3.99–4.04 (broad s, 4H, CH_2_-α (A), CH_2_-α (B)), 4.12–4.16 (two m, 2H, one of CH_2_-1' (A), one of CH_2_-1' (B)), 4.71–4.75 (m, 4H, one of CH_2_-10_sn-1_ (A), one of CH_2_-10_sn-1_ (B), one of CH_2_-10_sn-2_ (A), one of CH_2_-10_sn-2_ (B)), 4.80 (m, 4H, one of CH_2_-10_sn-1_ (A), one of CH_2_-10_sn-1_ (B), one of CH_2_-10_sn-2_ (A), one of CH_2_-10_sn-2_ (B)), 4.82–4.84 (m, 4H, H-6_sn-1_ (A), H-6_sn-1_ (B), H-6_sn-2_ (A), H-6_sn-2_ (B), 4.95 (m, 2H, H-2' (A), H-2' (B)), 5.54–5.60 (m, 4H, H-9_sn-1_ (A), H-9_sn-1_ (B), H-9_sn-2_ (A), H-9_sn-2_ (B); ^**13**^**C NMR** (151 MHz, CDCl_3_/CD_3_OD 2:1 (v/v)) δ: 16.91, 16.93, 24.97 (C-8_sn-1_ (A), C-8_sn-1_ (B), C-8_sn-2_ (A), C-8_sn-2_ (B), C-12_sn-1_ (A), C-12_sn-1_ (B), C-12_sn-2_ (A), C-12_sn-2_ (B)), 22.38 (C-5_sn-1_ (A), C-5_sn-1_ (B), C-5_sn-2_ (A), C-5_sn-2_ (B)), 22.42, 22.44, 22.45, 22.49 (C-11_sn-1_ (A), C-11_sn-1_ (B), C-11_sn-2_ (A), C-11_sn-2_ (B)), 38.69, 38.70, 38.76, 38.77 (C-3_sn-1_ (A), C-3_sn-1_ (B), C-3_sn-2_ (A), C-3_sn-2_ (B)), 40.28, 40.30 (C-4_sn-1_ (A), C-4_sn-1_ (B), C-4_sn-2_ (A), C-4_sn-2_ (B)), 44.40, 44.42, 44.44, 44.48 (C-2_sn-1_ (A), C-2_sn-1_ (B), C-2_sn-2_ (A), C-2_sn-2_ (B)), 53.64 (t, *J* = 3.6 Hz, -N(**C**H_3_)_3_(A), -N(**C**H_3_)_3_(B), 58.62 (d, *J* = 4.7 Hz, C-α (A), C-α (B)), 62.19, 62.20 (C-1' (A), C-1' (B)), 63.09, 63.12, (two d, *J* = 5.6 Hz, C-3' (A), C-3' (B)), 66.04 (m, C-β (A), C-β (B)), 69.92, 69.93 (two d, *J* = 8.0 Hz, C-2' (A), C-2' (B)), 111.81, 111.85 (C-10_sn-1_ (A), C-10_sn-1_ (B), C-10_sn-2_ (A), C-10_sn-2_ (B)), 123.84 (C-6_sn-1_ (A), C-6_sn-1_ (B), C-6_sn-2_ (A), C-6_sn-2_ (B)), 130.99 (C-7, C-7a), 144.74, 144.77 (C-9_sn-1_ (A), C-9_sn-1_ (B), C-9_sn-2_ (A), C-9_sn-2_ (B)), 170.91, 171.36 (C-1_sn-1_ (A), C-1_sn-1_ (B), C-1_sn-2_ (A), C-1_sn-2_ (B)); ^**31**^**P NMR** (243 MHz, CDCl_3_/CD_3_OD 2:1 (v/v)) δ: -0.74; HRMS (ESI): *m/z* calcd. for C_32_H_56_NO_8_P [M + H]^+^ 614.3822; found 614.3837

**(2'*R*)-1’,2’-di[(3*R*,6*E*)-3,7,11-trimethyl-3-vinyldodec-6,10-dienyl]-*sn*-glycero-3’-phosphocholine + (2'*R*)-1’,2-di[(3*S*,6*E*)-3,7,11-trimethyl-3-vinyldodec-6,10-dienyl]-*sn*-glycero-3’-phosphocholine (6b)**

mixture of two diasteroisomers, colourless greasy solid (86% yield, 98% purity (according to HPLC); ^**1**^**H NMR** (600 MHz, CDCl_3_/CD_3_OD 2:1 (v/v)), δ: 0.91, 0.92 (two s, 12H, CH_3_-15_sn-1_ (A), CH_3_-15_sn-1_ (B), CH_3_-15_sn-2_ (A), CH_3_-15_sn-2_ (B)), 1.17–1.22 (m, 8H, CH_2_-4_sn-1_ (A), CH_2_-4_sn-1_ (B), CH_2_-4_sn-2_ (A), CH_2_-4_sn-2_ (B)), 1.36, 1.45 (two s, 24H, CH_3_-12_sn-1_ (A), CH_3_-12_sn-1_ (B), CH_3_-12_sn-2_ (A), CH_3_-12_sn-2_ (B), CH_3_-17_sn-1_ (A), CH_3_-17_sn-1_ (B), CH_3_-17_sn-2_ (A), CH_3_-17_sn-2_ (B)), 1.37 (s, 12H, CH_3_-16_sn-1_ (A), CH_3_-16_sn-1_ (B), CH_3_-16_sn-2_ (A), CH_3_-16_sn-2_ (B)), 1.67–1.71 (m, 8H, CH_2_-5_sn-1_ (A), CH_2_-5_sn-1_ (B), CH_2_-5_sn-2_ (A), CH_2_-5_sn-2_ (B)), 1.72–1.75 (m, 8H, CH_2_-8_sn-1_ (A), CH_2_-8_sn-1_ (B), CH_2_-8_sn-2_ (A), CH_2_-8_sn-2_ (B)), 1.81–1.85 (m, 8H, CH_2_-9_sn-1_ (A), CH_2_-9_sn-1_ (B), CH_2_-9_sn-2_ (A), CH_2_-9_sn-2_ (B)), 2.09–2.18 (m, 8H, CH_2_-2_sn-1_ (A), CH_2_-2_sn-1_ (B), CH_2_-2_sn-2_ (A), CH_2_-2_sn-2_ (B)), 2.99 (s, 18H, -N(CH_3_)_3_ (A), -N(CH_3_)_3_ (B)), 3.36–3.40 (broad s, 4H, CH_2_-β (A), CH_2_-β (B)), 3.72–3.79 (m, 4H, CH_2_-3' (A), CH_2_-3' (B)), 3.89 (dd, *J* = 12.0, 6.6 Hz, 2H, one of CH_2_-1' (A), one of CH_2_-1' (B)), 4.00–4.05 (broad s, 4H, CH_2_-α (A), CH_2_-α (B)), 4.13 (m, 2H, one of CH_2_-1' (A), one of CH_2_-1' (B)), 4.73 (two d, *J* = 17.4 Hz, 4H, one of CH_2_-14_sn-1_ (A), one of CH_2_-14_sn-1_ (B), one of CH_2_-14_sn-2_ (A), one of CH_2_-14_sn-2_ (B)), 4.81 (two d, *J* = 10.8 Hz, 4H, one of CH_2_-14_sn-1_ (A), one of CH_2_-14_sn-1_ (B), one of CH_2_-14_sn-2_ (A), one of CH_2_-14_sn-2_ (B)), 4.85–4.87 (m, 8H, H-6_sn-1_ (A), H-6_sn-1_ (B), H-6_sn-2_ (A), H-6_sn-2_ (B), H-10_sn-1_ (A), H-10_sn-1_ (B), H-10_sn-2_ (A), H-10_sn-2_ (B)), 4.96 (m, 2H, H-2' (A), H-2' (B)), 5.56–5.61 (m, 4H, H-13_sn-1_ (A), H-13_sn-1_ (B), H-13_sn-2_ (A), H-13_sn-2_ (B)); ^**13**^**C NMR** (151 MHz, CDCl_3_/CD_3_OD 2:1 (v/v)) δ: 15.27, 24.99 (C-12_sn-1_ (A), C-12_sn-1_ (B), C-12_sn-2_ (A), C-12_sn-2_ (B), C-17_sn-1_ (A), C-17_sn-1_ (B), C-17_sn-2_ (A), C-17_sn-2_ (B)), 16.98 (C-16_sn-1_ (A), C-16_sn-1_ (B), C-16_sn-2_ (A), C-16_sn-2_ (B)), 22.27 (C-5_sn-1_ (A), C-5_sn-1_ (B), C-5_sn-2_ (A), C-5_sn-2_ (B)), 22.35, 22.40 (C-15_sn-1_ (A), C-15_sn-1_ (B), C-15_sn-2_ (A), C-15_sn-2_ (B)), 26.23 (C-9_sn-1_ (A), C-9_sn-1_ (B), C-9_sn-2_ (A), C-9_sn-2_ (B)), 38.72, 38.80 (C-3_sn-1_ (A), C-3_sn-1_ (B), C-3_sn-2_ (A), C-3_sn-2_ (B)), 39.24 (C-8_sn-1_ (A), C-8_sn-1_ (B), C-8_sn-2_ (A), C-8_sn-2_ (B)), 40.39, 40.42 (C-4_sn-1_ (A), C-4_sn-1_ (B), C-4_sn-2_ (A), C-4_sn-2_ (B)), 44.44, 44.47, 44.48, 44.54 (C-2_sn-1_ (A), C-2_sn-1_ (B), C-2_sn-2_ (A), C-2_sn-2_ (B)), 53.63 (m, -N(**C**H_3_)_3_ (A), -N(**C**H_3_)_3_ (B)), 58.65 (m, C-α (A), C-α (B)), 62.18 (C-1' (A), C-1' (B)), 63.16 (m, C-3' (A), C-3' (B)), 66.01 (m, C-β (A), C-β), 69.90 (d, *J* = 6.0 Hz, C-2' (A), C-2' (B)), 111.84, 111.88 (C-14_sn-1_ (A), C-14_sn-1_ (B), C-14_sn-2_ (A), C-14_sn-2_ (B),), 123.72 (C-10_sn-1_ (A), C-10_sn-1_ (B), C-10_sn-2_ (A), C-10_sn-2_ (B)), 123.83 (C-6_sn-1_ (A), C-6_sn-1_ (B), C-6_sn-2_ (A), C-6_sn-2_ (B)), 130.81 (C-11_sn-1_ (A), C-11_sn-1_ (B), C-11_sn-2_ (A), C-11_sn-2_ (B)), 134.67 (C-7_sn-1_ (A), C-7_sn-1_ (B), C-7_sn-2_ (A), C-7_sn-2_ (B)), 144.71, 144.75 (C-13_sn-1_ (A), C-13_sn-1_ (B)), C-13_sn-2_ (A), C-13_sn-2_ (B)), 170.91, 171.35 (C-1_sn-1_ (A), C-1_sn-1_ (B), C-1_sn-2_ (A), C-1_sn-2_ (B)); ^**31**^**P NMR** (243 MHz, CDCl_3_/CD_3_OD 2:1 (v/v)) -0.80; HRMS (ESI): *m/z* calcd. for C_42_H_72_NO_8_P [M + H]^+^ 750.5074; found 750.5082

**(2’*R*)-1’,2’-di{2-[(3*R*,2"*E*)-2"-butylidene-1",3",3"-trimethyl]cyclohexyl}acetyl*-sn*-glycero-3’-phospocholine + (2’*R*)-1’,2’-di{2-[(3*S*,2"*E*)-2"-butylidene-1",3",3"-trimethyl] cyclohexyl}acetyl*-sn*-glycero-3’-phospocholine (6c)**

mixture of two diasteroisomers, colourless greasy solid (62% yield, 99% purity (according to HPLC); ^**1**^**H NMR** (600 MHz, CDCl_3_/CD_3_OD 2:1 (v/v)), δ: 0.66–0.69 (two t, *J* = 7.2 Hz, 12H, CH_3_-10_sn-1_ (A), CH_3_-10_sn-1_ (B), CH_3_-10_sn-2_ (A), CH_3_-10_sn-2_ (B)), 0.96, 0.97, 0.98, 0.99 (four s, 36H, CH_3_-11_sn-1_ (A), CH_3_-11_sn-1_ (B), CH_3_-11_sn-2_ (A), CH_3_-11_sn-2_ (B), CH_3_-12_sn-1_ (A), CH_3_-12_sn-1_ (B), CH_3_-12_sn-2_ (A), CH_3_-12_sn-2_ (B), CH_3_-13_sn-1_ (A), CH_3_-13_sn-1_ (B), CH_3_-13_sn-2_ (A), CH_3_-13_sn-2_ (B)), 1.11–1.17 (m, 16H, CH_2_-9_sn-1_ (A), CH_2_-9_sn-1_ (B), CH_2_-9_sn-2_ (A), CH_2_-9_sn-2_ (B), one of CH_2_-6_sn-1_ (A), one of CH_2_-6_sn-1_ (B), one of CH_2_-6_sn-2_ (A), one of CH_2_-6_sn-2_ (B), one of CH_2_-5_sn-1_ (A), one of CH_2_-5_sn-1_ (B), one of CH_2_-5_sn-2_ (A), one of CH_2_-5_sn-2_ (B)), 1.31–1.40 (m, 12H, CH_2_-4_sn-1_ (A), CH_2_-4_sn-1_ (B), CH_2_-4_sn-2_ (A), CH_2_-4_sn-2_ (B), one of CH_2_-5_sn-1_ (A), one of CH_2_-5_sn-1_ (B), one of CH_2_-5_sn-2_ (A), one of CH_2_-5_sn-2_ (B)), 1.49–1.54 (m, 4H, one of CH_2_-6_sn-1_ (A), one of CH_2_-6_sn-1_ (B), one of CH_2_-6_sn-2_ (A), one of CH_2_-6_sn-2_ (B)), 1.89–1.99 (m, 4H, CH_2_-8_sn-1_ (A), CH_2_-8_sn-1_ (B), CH_2_-8_sn-2_ (A), CH_2_-8_sn-2_ (B)), 2.11–2.41 (two m, 8H, CH_2_-14_sn-1_ (A), CH_2_-14_sn-1_ (B), CH_2_-14_sn-2_ (A), CH_2_-14_sn-2_ (B)), 2.99 (s, 18H, -N(CH_3_)_3_ (A), -N(CH_3_)_3_ (B)), 3.38 (m, 4H, CH_2_-β (A), CH_2_-β (B)), 3.70–3.75 (m, 4H, CH_2_-3' (A), CH_2_-3' (B)), 3.84–3.92 (m, 2H, one of CH_2_-1' (A), one of CH_2_-1' (B)), 3.99–4.04 (broad s, 2H, CH_2_-α (A), CH_2_-α (B)), 4.05–4.11 (m, 2H, one of CH_2_-1' (A), one of CH_2_-1' (B)), 4.90–4.96 (m, 2H, H-2' (A), H-2' (B)), 4.97–5.01 (m, 4H, H-7_sn-1_ (A), H-7_sn-1_ (B), H-7_sn-2_ (A), H-7_sn-2_ (B)); ^**13**^**C NMR** (151 MHz, CDCl_3_/CD_3_OD 2:1 (v/v)) δ: 13.27 (C-10_sn-1_ (A), C-10_sn-1_ (B), C-10_sn-2_ (A), C-10_sn-2_ (B)), 17.03, 17.06 (C-4_sn-1_ (A), C-4_sn-1_ (B), C-4_sn-2_ (A), C-4_sn-2_ (B)), 23.27, 23.28 (C-9_sn-1_ (A), C-9_sn-1_ (B), C-9_sn-2_ (A), C-9_sn-2_ (B)), 29.92, 29.97, 30.13, 30.15, 30.23 (C-11_sn-1_ (A), C-11_sn-1_ (B), C-11_sn-2_ (A), C-11_sn-2_ (B), C-12_sn-1_ (A), C-12_sn-1_ (B), C-12_sn-2_ (A), C-12_sn-2_ (B), 30.59, 30.69, 30.77, 30.90 (C-13_sn-1_ (A), C-13_sn-1_ (B), C-13_sn-2_ (A), C-13_sn-2_ (B)), 31.79, 31.81 (C-8_sn-1_ (A), C-8_sn-1_ (B), C-8_sn-2_ (A), C-8_sn-2_ (B)), 34.85 (C-3_sn-1_ (A), C-3_sn-1_ (B), C-3_sn-2_ (A), C-3_sn-2_ (B)), 35.22 (C-6_sn-1_ (A), C-6_sn-1_ (B), C-6_sn-2_ (A), C-6_sn-2_ (B)), 39.04, 39.05 (C-1_sn-1_ (A), C-1_sn-1_ (B), C-1_sn-2_ (A), C-1_sn-2_ (B)), 40.72, 40.76 (C-5_sn-1_ (A), C-5_sn-1_ (B), C-5_sn-2_ (A), C-5_sn-2_ (B)), 47.02, 47.04, 47.09 47.19 (C-14_sn-1_ (A), C-14_sn-1_ (B), C-14_sn-2_ (A), C-14_sn-2_ (B)), 53.61 (t, *J* = 3.6 Hz, -N(**C**H_3_)_3_ (A), -N(**C**H_3_)_3_ (B)), 58.61 (d, *J* = 4.8 Hz, C-α (A), C-α (B)), 62.05, 62.10 (C-1' (A), C-1’ (B)), 63.08 (d, *J* = 4.9 Hz, C-3' (A), C-3' (B)), 66.02 (m, C-β (A), C-β (B)), 69.78 (d, *J* = 8.0 Hz, C-2' (A)), 69.82 (d, *J* = 8.6 Hz, C-2' (B)), 126.39, 126.40, 126.47, 126.51 (C-7_sn-1_ (A), C-7_sn-1_ (B), C-7_sn-2_ (A), C-7_sn-2_ (B)), 148.83, 148.88, 149.10, 149.15 (C-2_sn-1_ (A), C-2_sn-1_ (B)), C-2_sn-2_ (A), C-2_sn-2_ (B)), 171.25, 171.73 (C-15_sn-1_ (A), C-15_sn-1_ (B), C-15_sn-2_ (A), C-15_sn-2_ (B)); ^**31**^**P NMR** (243 MHz, CDCl_3_/CD_3_OD 2:1 (v/v)) δ: -0.67; HRMS (ESI): *m/z* calcd. for C_38_H_68_NO_8_P [M + H]^+^ 698.4761; found 698.4766

**(2'*R*)-1’,2’-di[(3*R*,7*R*,11*R*)-3,7,11,15-tetramethyl-3-vinylhexadecyl]-*sn*-glycero-3’-phosphocholine + (2'*R*)-1’,2’-di[(3*S*,7*R*,11*R*)-3,7,11,15-tetramethyl-3-vinylhexadecyl]-*sn*-glycero-3’-phosphocholine (6d)**

mixture of two diasteroisomers, colourless greasy solid (74% yield, 98% purity (according to HPLC); ^**1**^**H NMR** (600 MHz, CDCl_3_/CD_3_OD 2:1 (v/v)), δ: 0.61–0.66 (m, 48H, CH_3_-16_sn-1_ (B), CH_3_-16_sn-2_ (A), CH_3_-16_sn-1_ (A), CH_3_-16_sn-2_ (B), CH_3_-20_sn-1_ (A), CH_3_-20_sn-1_ (B), CH_3_-20_sn-2_ (A), CH_3_-20_sn-2_ (B), CH_3_-21_sn-1_ (A), CH_3_-21_sn-1_ (B), CH_3_-21_sn-2_ (A), CH_3_-21_sn-2_ (B), CH_3_-22_sn-1_ (a), CH_3_-22_sn-1_ (B), CH_3_-22_sn-2_ (A), CH_3_-22_sn-2_ (B)), 0.84–1.15 (four m, 92H, CH_3_-19_sn-1_ (A), CH_3_-19_sn-1_ (B), CH_3_-19_sn-2_ (A), CH_3_-19_sn-2_ (B), CH_2_-4_sn-1_ (A), CH_2_-4_sn-1_ (B), CH_2_-4_sn-2_ (A), CH_2_-4_sn-2_ (B), CH_2_-5_sn-1_ (A), CH_2_-5_sn-1_ (B), CH_2_-5_sn-2_ (A), CH_2_-5_sn-2_ (B), CH_2_-6_sn-1_ (A), CH_2_-6_sn-1_ (B), CH_2_-6_sn-2_ (A), CH_2_-6_sn-2_ (B), CH_2_-8_sn-1_ (A), CH_2_-8_sn-1_ (B), CH_2_-8_sn-2_ (A), CH_2_-8_sn-2_ (B), CH_2_-9_sn-1_ (A), CH_2_-9_sn-1_ (B), CH_2_-9_sn-2_ (A), CH_2_-9_sn-2_ (B), CH_2_-10_sn-1_ (A), CH_2_-10_sn-1_ (B), CH_2_-10_sn-2_ (A), CH_2_-10_sn-2_ (B), CH_2_-12_sn-1_ (A), CH_2_-12_sn-1_ (B), CH_2_-12_sn-2_ (A), CH_2_-12_sn-2_ (B), CH_2_-13_sn-1_ (A), CH_2_-13_sn-1_ (B), CH_2_-13_sn-2_ (A), CH_2_-13_sn-2_ (B), CH_2_-14_sn-1_ (A), CH_2_-14_sn-1_ (B), CH_2_-14_sn-2_ (A), CH_2_-14_sn-2_ (B), H-7_sn-1_ (A), H-7_sn-1_ (B), H-7_sn-2_ (A), H-7_sn-2_ (B), H-11_sn-1_ (A), H-11_sn-1_ (B), H-11_sn-2_ (A), H-11_sn-2_ (B)), 1.26–1.34 (m, 4H, H-15_sn-1_ (A), H-15_sn-1_ (B), H-15_sn-2_ (A), H-15_sn-2_ (B)), 2.07–2.15 (m, 8H, CH_2_-2_sn-1_ (A), CH_2_-2_sn-1_ (B), CH_2_-2_sn-2_ (A), CH_2_-2_sn-2_ (B)), 2.99 (s, 18H, -N(CH_3_)_3_ (A), -N(CH_3_)_3_ (B)), 3.40 (m, 4H, CH_2_-β (A), CH_2_-β (B)), 3.75–3.77 (m, 4H, CH_2_-3' (A), CH_2_-3' (B)), 3.84–3.88 (m, 2H, one of CH_2_-1' (A), one of CH_2_-1' (B)), 4.01–4.08 (m, 4H, CH_2_-α (A), CH_2_-α (B)), 4.11–4.17 (m, 2H, one of CH_2_-1' (A), one of CH_2_-1' (B)), 4.69–4.75 (m, 4H, one of CH_2_-18_sn-1_ (A), one of CH_2_-18_sn-1_ (B), one of CH_2_-18_sn-2_ (A), one of CH_2_-18_sn-2_ (B)), 4.77–4.81 (m, 2H, one of CH_2_-18_sn-1_ (A), one of CH_2_-18_sn-1_ (B), one of CH_2_-18_sn-2_ (A), one of CH_2_-18_sn-2_ (B)), 4.95 (m, 2H, H-2' (A), H-2' (B)), 5.53–5.62 (m, 4H, H-17_sn-1_ (A), H-17_sn-1_ (B), H-17_sn-2_ (A), H-17_sn-2_ (B)); ^**13**^**C NMR** (151 MHz, CDCl_3_/CD_3_OD 2:1 (v/v)) δ: 19.12, 19.18, 19.25 (C-20_sn-1_ (A), C-20_sn-1_ (B), C-20_sn-2_ (A), C-20_sn-2_ (B), C-21_sn-1_ (A), C-21_sn-1_ (B), C-21_sn-2_ (A), C-21_sn-2_ (B)), 21.12, 21.13, 24.07, 24.41, 24.42, 29.27, 36.89, 37.00, 37.02, 37.06, 37.26, 37.28, 37.31, 37.37, 37.40, 38.99, 41.00 (C-4_sn-1_ (A), C-4_sn-1_ (B), C-4_sn-2_ (A), C-4_sn-2_ (B), C-5_sn-1_ (A), C-5_sn-1_ (B), C-5_sn-2_ (A), C-5_sn-2_ (B), C-6_sn-1_ (A), C-6_sn-1_ (B), C-6_sn-2_ (A), C-6_sn-2_ (B), C-8_sn-1_ (A), C-8_sn-1_ (B), C-8_sn-2_ (A), C-8_sn-2_ (B), C-9_sn-1_ (A), C-9_sn-1_ (B), C-9_sn-2_ (A), C-9_sn-2_ (B), C-10_sn-1_ (A), C-10_sn-1_ (B), C-10_sn-2_ (A), C-10_sn-2_ (B), C-12_sn-1_ (A), C-12_sn-1_ (B), C-12_sn-2_ (A), C-12_sn-2_ (B), C-13_sn-1_ (A), C-13_sn-1_ (B), C-13_sn-2_ (A), C-13_sn-2_ (B), C-14_sn-1_ (A), C-14_sn-1_ (B), C-14_sn-2_ (A), C-14_sn-2_ (B), 22.09, 22.18 (C-16_sn-1_ (A), C-16_sn-1_ (B), C-16_sn-2_ (A), C-16_sn-2_ (B), C-22_sn-1_ (A), C-22_sn-1_ (B), C-22_sn-2_ (A), C-22_sn-2_ (B)), 22.44, 22.49, 22.50, 22.51 (C-19_sn-1_ (A), C-19_sn-1_ (B), C-19_sn-2_ (A), C-19_sn-2_ (B)), 27.58 (C-15_sn-1_ (A), C-15_sn-1_ (B), C-15_sn-2_ (A), C-15_sn-2_ (B)), 32.39, 32.40 (C-7_sn-1_ (A), C-7_sn-1_ (B), C-7_sn-2_ (A), C-7_sn-2_ (B), C-11_sn-1_ (A), C-11_sn-1_ (B), C-11_sn-2_ (A), C-11_sn-2_ (B)), 38.84, 38.85, 38.92, 38.93 (C-3_sn-1_ (A), C-3_sn-1_ (B), C-3_sn-1_ (A), C-3_sn-2_ (B)), 44.76, 44.83 (C-2_sn-1_ (A), C-2_sn-2_ (A), C-2_sn-1_ (B), C-2_sn-2_ (B)), 53.75 (t, *J* = 3.5 Hz, -N(**C**H_3_)_3_ (A), -N(**C**H_3_)_3_ (B)), 58.83 (d, *J* = 5.4 Hz, C-α (A), C-α (B)), 62.17 (C-1' (A), C-1' (B)), 63.38 (d, *J* = 4.1 Hz, C-3' (A), C-3' (B)), 66.04 (m, C-β (A), C-β (B)), 69.85 (d, *J* = 9.0 Hz, C-2' (A), C-2' (B)), 111.75, 111.79 (C-18_sn-1_ (A), C-18_sn-1_ (B), C-18_sn-2_ (A), C-18_sn-2_ (B)), 145.04 (C-17_sn-1_ (A), C-17_sn-1_ (B), C-17_sn-2_ (A), C-17_sn-2_ (B)), 171.01, 171.46 (C-1_sn-1_ (A), C-1_sn-1_ (B), C-1_sn-2_ (A), C-1_sn-2_ (B)); ^**31**^**P NMR** (243 MHz, CDCl_3_/CD_3_OD 2:1 (v/v)) δ: -0.93; HRMS (ESI): *m/z* calcd. for C_52_H_100_NO_8_P [M + H]^+^ 898.7265; found 898.7261

**1-palmitoyl-2-isoprenoyl-*sn*-glycero-3’-phosphatidylcholines**

**(2’*R*)-1’-palmitoyl-2’-[(3*R*)-3,7-dimethyl-3-vinylocta-6-enyl]*-sn*-glycero-3’-phosphocholine + (2’*R*)-1’-palmitoyl-2’-[(3*S*)-3,7-dimethyl-3-vinylocta-6-enyl]*-sn*-glycero-3’-phosphocholine (7a)**

mixture of two diasteroisomers, colourless greasy solid (58% yield, 99% purity (according to HPLC); ^**1**^**H NMR** (600 MHz, CDCl_3_/CD_3_OD 2:1 (v/v)), δ: 0.62 (t, *J* = 7.2 Hz, 6H, C**H**_**3**_(CH_2_)_14_C(O) (A), C**H**_**3**_(CH_2_)_14_C(O) (B)), 0.88 (s, 6H, CH_3_-11 (A), CH_3_-11 (B)), 1.00–1.03 (m, 48H, CH_3_(C**H**_**2**_)_**12**_CH_2_CH_2_C(O) (A), CH_3_(C**H**_**2**_)_**12**_CH_2_CH_2_C(O) (B)), 1.15 (m, 4H, CH_2_-4 (A), CH_2_-4 (B)), 1.32, 1.40 (two s, 12H, CH_3_-8 (A), CH_3_-8 (B), CH_3_-12 (A), CH_3_-12 (B)), 1.33–1.36 (m, 4H, CH_3_(CH_2_)_13_C**H**_**2**_CH_2_C(O) (A), CH_3_(CH_2_)_13_C**H**_**2**_CH_2_C(O) (B)), 1.65 (m, 4H, CH_2_-5 (A), CH_2_-5 (B)), 2.05 (t, *J* = 7.8 Hz, 4H, CH_3_(CH_2_)_13_C**H**_**2**_C(O) (A), CH_3_(CH_2_)_13_C**H**_**2**_C(O) (B)), 2.07–2.14 (four d, *J* = 13.2 Hz, two AB systems, 4H, CH_2_-2 (A), CH_2_-2 (B)), 2.96 (s, 18H, -N(CH_3_)_3_ (A), -N(CH_3_)_3_ (B)), 3.35 (m, 4H, CH_2_-β (A), CH_2_-β (B)), 3.69–3.73 (m, 4H, CH_2_-3' (A), CH_2_-3' (B)), 3.87 (m, 2H, one of CH_2_-1' (A), one of CH_2_-1' (B)), 3.99 (m, 4H, CH_2_-α (A), CH_2_-α (B)), 4.14 (m, 2H, one of CH_2_-1' (A), one of CH_2_-1' (B)), 4.68–4.72 (two d, *J* = 17.4, 2H, one of CH_2_-10 (A), one of CH_2_-10 (B)), 4.77–4.79 (two d, *J* = 10.8 Hz, 2H, one of CH_2_-10 (A), one of CH_2_-10 (B)), 4.78–4.80 (m, 2H, H-6 (A), H-6 (B)), 4.94–4.97 (m, 2H, H-2' (A), H-2' (B)), 5.52–5.57 (two dd, *J* = 17.4, 10.8 Hz, 2H, H-9 (A), H-9 (B)); ^**13**^**C NMR** (151 MHz, CDCl_3_/CD_3_OD 2:1 (v/v)) δ: 13.37 (**C**H_3_(CH_2_)_14_C(O) (A), (**C**H_3_(CH_2_)_14_C(O) (B)), 16.85, 24.91 (C-8 (A), C-8 (B), C-12 (A), C-12 (B)), 22.15 (CH_3_**C**H_2_(CH_2_)_13_C(O) (A), CH_3_**C**H_2_(CH_2_)_13_C(O) (B)), 22.33, 22.35 (C-5 (A), C-5 (B)), 22.43, 22.47 (C-11 (A), C-11 (B)), 24.33 (CH_3_(CH_2_)_12_**C**H_2_CH_2_C(O) (A), CH_3_(CH_2_)_12_**C**H_2_CH_2_C(O) (B)), 28.64, 28.78, 28.84, 28.98, 29.11, 29.13, 29.16, 31.42 (CH_3_CH_2_(**C**H_2_)_11_CH_2_CH_2_C(O) (A), CH_3_CH_2_(**C**H_2_)_11_CH_2_CH_2_C(O) (B)), 33.57 (CH_3_(CH_2_)_13_**C**H_2_C(O) (A), CH_3_(CH_2_)_13_**C**H_2_C(O) (B)), 38.73, 38.75 (C-3 (A), C-3 (B)), 40.21, 40.22 (C-4 (A), C-4 (B)), 44.38, 44.43 (C-2 (A), C-2 (B)), 53.56 (t, *J* = 3.6 Hz, -N(**C**H_3_)_3_ (A), -N(**C**H_3_)_3_ (B)), 58.59 (d, *J* = 4.9 Hz, C-α (A), C-α (B)), 62.39, 62.40 (C-1' (A), C-1' (B)), 63.04 (d, *J* = 5.1 Hz (C-3' (A)), 63.07 (d, *J* = 5.3 Hz, (C-3' (B)), 65.98 (m, C-β (A), C-β (B)), 69.88 (d, *J* = 8.0 Hz, C-2' (A), C-2' (B)), 111.75 (C-10 (A), C-10 (B)), 123.80 (C-6 (A), C-6 (B)), 130.92 (C-7 (A), C-7 (B)), 144.73, 144.76 (C-9 (A), C-9 (B)), 170.95 (C-1 (A), C-1 (B)), 173.59 (CH_3_(CH_2_)_13_CH_2_**C**(O) (A), CH_3_(CH_2_)_13_CH_2_**C**(O) (B)); ^**31**^**P NMR** (243 MHz, CDCl_3_/CD_3_OD 2:1 (v/v)) δ: -0.80; HRMS (ESI): *m/z* calcd. for C_36_H_68_NO_8_P [M + H]^+^ 674.4761; found 674.4768

**(2'*R*)-1’-palmitoyl-2’-[(3*R*,6*E*)-3,7,11-trimethyl-3-vinyldodeca-6,10-dienyl]*-sn*-glycero-3’-phosphocholine + (2'*R*)-1’-palmitoyl-2’-[(3*S*,6*E*)-3,7,11-trimethyl-3-vinyldodeca-6,10-dienyl]*-sn*-glycero-3’-phosphocholine (7b)**

mixture of two diasteroisomers, colourless greasy solid (54% yield, 99% purity (according to HPLC); ^**1**^**H NMR** (600 MHz, CDCl_3_/CD_3_OD 2:1 (v/v)), δ: 0.61 (t, *J* = 6.6 Hz, 6H, C**H**_**3**_(CH_2_)_13_CH_2_C(O) (A), C**H**_**3**_(CH_2_)_13_CH_2_C(O) (B)), 0.87 (s, 6H, CH_3_-15 (A), CH_3_-15 (B)), 0.98–1.05 (m, 48H, CH_3_(C**H**_**2**_)_12_CH_2_CH_2_C(O) (A), CH_3_(C**H**_**2**_)_12_CH_2_CH_2_C(O) (B)), 1.16–1.17 (m, 4H, CH_2_-4 (A), CH_2_-4 (B)), 1.31, 1.40 (two s, 12H, CH_3_-12 (A), CH_3_-12 (B), CH_3_-17 (A), CH_3_-17 (B)), 1.33–1.36 (m, 4H, CH_3_(CH_2_)_12_C**H**_**2**_CH_2_C(O) (A), CH_3_(CH_2_)_12_C**H**_**2**_CH_2_C(O) (B)), 1.33 (s, 6H, CH_3_-16 (A), CH_3_-16 (B)), 1.64–1.70 (two m, 8H, CH_2_-5 (A), CH_2_-5 (B), CH_2_-8 (A), CH_2_-8 (B)), 1.77–1.80 (m, 4H, CH_2_-9 (A), CH_2_-9 (B)), 2.05 (t, *J* = 7.8 Hz, 4H, CH_3_(CH_2_)_13_C**H**_**2**_C(O) (A), CH_3_(CH_2_)_13_C**H**_**2**_C(O) (B)), 2.06–2.14 (two d, *J* = 13.8 Hz, 4H, two AB systems, CH_2_-2 (A), CH_2_-2 (B)), 2.96 (s, 18H, -N(CH_3_)_3_ (A), -N(CH_3_)_3_ (B)), 3.36–3.39 (broad s, 4H, CH_2_-β (A), CH_2_-β (B)), 3.74–3.76 (m, 4H, CH_2_-3' (A), CH_2_-3' (B)), 3.87 (dd, *J* = 12.0, 6.6 Hz, 2H, one of CH_2_-1' (A), one of CH_2_-1' (B)), 4.00–4.05 (broad s, 4H, CH_2_-α (A), CH_2_-α (B)), 4.13 (m, 2H, one of CH_2_-1' (A), one of CH_2_-1' (B)), 4.70 (d, *J* = 17.4, 2H, one of CH_2_-14 (A), one of CH_2_-14 (B)), 4.75–4.78 (two d, *J* = 10.8 Hz, 2H, one of CH_2_-14 (A), one of CH_2_-14 (B)), 4.79–4.84 (m, 4H, H-6 (A), H-6 (B), H-10 (A), H-10 (B)), 4.93–4.98 (m, 2H, H-2' (A), H-2' (B)), 5.52–5.57 (two dd, *J* = 17.4, 10.8 Hz, 2H, H-13 (A), H-13 (B)); ^**13**^**C NMR** (151 MHz, CDCl_3_/CD_3_OD 2:1 (v/v)) δ: 13.33 (**C**H_3_(CH_2_)_13_CH_2_C(O) (A), **C**H_3_(CH_2_)_13_CH_2_C(O) (B)), 15.18, 24.89 ((C-12 (A), C-12 (B), C-17 (A), C-17 (B)), 16.87 (C-16 (A), C-16 (B)), 22.13 (CH_3_**C**H_2_(CH_2_)_12_CH_2_C(O) (A), CH_3_**C**H_2_(CH_2_)_12_CH_2_C(O) (B)), 22.23, 22.24 (C-5 (A), C-5 (B)), 22.37, 22.41 (C-15 (A), C-15 (B)), 24.32 (CH_3_(CH_2_)_12_**C**H_2_CH_2_C(O) (A), CH_3_(CH_2_)_12_**C**H_2_CH_2_C(O) (B)), 26.18 (C-9 (A), C-9 (B)), 28.62, 28.78, 28.82, 28.97, 29.10, 29.12, 29.13, 29.15, 31.40 (CH_3_CH_2_(**C**H_2_)_11_CH_2_CH_2_C(O) (A), CH_3_CH_2_(**C**H_2_)_11_CH_2_CH_2_C(O) (B)), 33.55 CH_3_(CH_2_)_13_**C**H_2_C(O) (A), CH_3_(CH_2_)_13_**C**H_2_C(O) (B)), 38.75, 38.78 (C-3 (A), C-3 (B)), 39.20 (C-8 (A), C-8 (B)), 40.29 (C-4 (A), C-4 (B)), 44.36, 44.44 (C-2 (A), C-2 (B)), 53.52 (t, *J* = 3.5 Hz, -N(**C**H_3_)_3_ (A), -N(**C**H_3_)_3_ (B)), 58.89 (d, *J* = 4.9 Hz, C-α (A), C-α (B)), 62.22 (C-1' (A), C-1' (B)), 63.36 (d, *J* = 5.7 Hz, C-3' (A), C-3' (B)), 65.84 (m, C-β (A), C-β (B)), 69.70 (d, *J* = 8.1 Hz, C-2' (A), C-2' (B)), 111.76 (C-14 (A), C-14 (B)), 123.67 (C-10 (A), C-10 (B)), 123.77 (C-6 (A), C-6 (B)), 130.72 (C-11 (A), C-11 (B)), 134.61 (C-7 (A), C-7 (B)), 144.70, 144.74 (C-13 (A), C-13 (B)), 170.87 (C-1 (A), C-1 (B)), 173.51 (CH_3_(CH_2_)_13_CH_2_**C**(O) (A), CH_3_(CH_2_)_13_CH_2_**C**(O) (B));^**31**^**P NMR** (243 MHz, CDCl_3_/CD_3_OD 2:1 (v/v)) δ: -0.97; HRMS (ESI): *m/z* calcd. for C_41_H_76_NO_8_P [M + H]^+^ 742.5387; found 742.5387

**(2’*R*)-1’-palmitoyl-2’-{2-[(3*R*,2"*E*)-2"-butylidene-1",3",3"-trimethyl]cyclohexyl}acetyl*-sn*-glycero-3’-phospocholine + (2’*R*)-1’-palmitoyl-2’-{2-[(3*S*,2"*E*)-2"-butylidene-1",3",3"-trimethyl]cyclohexyl}acetyl*-sn*-glycero-3’-phospocholine (7c)**

mixture of two diasteroisomers, colourless greasy solid (41% yield, 98% purity (according to HPLC); ^**1**^**H NMR** (600 MHz, CDCl_3_/CD_3_OD 2:1 (v/v)), δ: 0.65 (t, *J* = 6.6 Hz, 6H, C**H**_**3**_(CH_2_)_13_CH_2_C(O) (A), C**H**_**3**_(CH_2_)_13_CH_2_C(O) (B)), 0.69 (t, *J* = 7.2 Hz, 6H, CH_3_-10 (A), CH_3_-10 (B)), 0.97 (s, 6H, CH_3_-13 (A), CH_3_-13 (B)), 0.98 (s, 12H, CH_3_-11 (A), CH_3_-11 (B), CH_3_-12 (A), CH_3_-12 (B)), 1.03–1.06 (m, 48H, CH_3_(C**H**_**2**_)_12_CH_2_CH_2_C(O) (A), CH_3_(C**H**_**2**_)_12_CH_2_CH_2_C(O) (B)), 1.14–1.18 (m, 8H, CH_2_-9 (A), CH_2_-9 (B), one of CH_2_-5 (A), one of CH_2_-5 (B), one of CH_2_-6 (A), one of CH_2_-6 (B)), 1.33–1.41 (m, 10H, CH_2_-4 (A), CH_2_-4 (B), CH_3_(CH_2_)_12_C**H**_**2**_CH_2_C(O) (A), CH_3_(CH_2_)_12_C**H**_**2**_CH_2_C(O) (B), one of CH_2_-5 (A), one of CH_2_-5 (B)), 1.49–1.55 (m, 2H, one of CH_2_-6 (A), one of CH_2_-6 (B)), 1.89–2.00 (m, 4H, CH_2_-8 (A), CH_2_-8 (B)), 2.08 (t, *J* = 6.6 Hz, 4H, CH_3_(CH_2_)_13_C**H**_**2**_C(O) (A), CH_3_(CH_2_)_13_C**H**_**2**_C(O) (B)), 2.10–2.43 (four d, *J* = 13.8 Hz, two AB systems, 4H, CH_2_-14 (A), CH_2_-14 (B)), 2.99 (s, 18H, -N(CH_3_)_3_ (A), -N(CH_3_)_3_ (B)), 3.38 (m, 4H, CH_2_-β (A), CH_2_-β (B)), 3.73–3.78 (m, 4H, CH_2_-3' (A), CH_2_-3' (B)), 3.91–3.95 (two dd, *J* = 12.0, 6.6 Hz, 2H, one of CH_2_-1' (A), one of CH_2_-1' (B), 4.00–4.05 (broad m, 4H, CH_2_-α (A), CH_2_-α (B)), 4.13–4.16 (two m, 2H, one of CH_2_-1' (A), one of CH_2_-1' (B)), 4.93–4.98 (m, 2H, H-2' (A), H-2' (B)), 4.97–5.02 (two t, *J* = 7.2 Hz, 2H, H-7 (A), H-7 (B)); ^**13**^**C NMR** (151 MHz, CDCl_3_/CD_3_OD 2:1 (v/v)) δ: 13.31 (**C**H_3_(CH_2_)_13_CH_2_C(O) (A), **C**H_3_(CH_2_)_13_CH_2_C(O) (B)), 13.47 (C-10 (A), C-10 (B)), 17.03, 17.08 (C-4 (A), C-4 (B)), 22.21 (CH_3_**C**H_2_(CH_2_)_12_CH_2_C(O) (A), CH_3_**C**H_2_(CH_2_)_12_CH_2_C(O) (B)), 23.30 (C-9 (A), C-9 (B)), 24.39 (CH_3_(CH_2_)_12_**C**H_2_CH_2_C(O) (A), CH_3_(CH_2_)_12_**C**H_2_CH_2_C(O) (B)), 28.70, 28.84 28.90 29.03 29.19 29.23, 31.47 (CH_3_CH_2_(**C**H_2_)_11_CH_2_CH_2_C(O) (A), CH_3_CH_2_(**C**H_2_)_11_CH_2_CH_2_C(O) (B)), 29.93, 29.99, 30.23, 30.26, 30.64, 30.77 (C-11 (A), C-11 (B), C-12 (A), C-12 (B), C-13 (A), C-13 (B)), 31.85 (C-8 (A), C-8 (B)), 33.63, 33.65 (CH_3_(CH_2_)_13_**C**H_2_C(O) (A), CH_3_(CH_2_)_13_**C**H_2_C(O) (B)), 34.87 (C-3 (A), C-3 (B)), 35.30, 35.31 (C-6 (A), C-6 (B)), 39.09 (C-1 (A), C-1 (B)), 40.74, 40.77 (C-5 (A), C-5 (B)), 47.19, 47.21 (C-14 (A), C-14 (B)), 53.65 (t, *J* = 3.3 Hz, -N(**C**H_3_)_3_ (A), -N(**C**H_3_)_3_ (B)), 58.62 (d, *J* = 4.7 Hz, C-α (A), C-α (B)), 62.46, 62.49 (C-1' (A), C-1' (B)), 63.03 (d, *J* = 5.4 Hz, C-3' (A), C-3' (B)) 66.05 (m, C-β (A), C-β (B)), 69.70, 69.75 (two d, *J* = 7.5 Hz, C-2' (A), C-2' (B)), 126.41, 126.56 (C-7 (A), C-7 (B)), 149.00, 149.11 (C-2 (A), C-2 (B)), 171.32 (C-15 (A), C-15 (B)), 173.64 (CH_3_(CH_2_)_13_CH_2_**C**(O) (A), CH_3_(CH_2_)_13_CH_2_**C**(O) (B)); ^**31**^**P NMR** (243 MHz, CDCl_3_/CD_3_OD 2:1 (v/v)) δ: -0.83; HRMS (ESI): *m/z* calcd. for C_39_H_74_NO_8_P [M + H]^+^ 716.5230; found 716.5206

**(2'*R*)-1’-palmitoyl-2’-[(3*R*,7*R*,11*R*)-3,7,11,15-tetramethyl-3-vinylhexadecyl]-*sn*-glycero-3’-phosphocholine + (2'*R*)-1’-palmitoyl-2’-[(3*S*,7*R*,11*R*)-3,7,11,15-tetramethyl-3-vinylhexadecyl]-*sn*-glycero-3’-phosphocholine (7d)**

mixture of two diasteroisomers, colourless greasy solid (51% yield, 99% purity (according to HPLC); ^**1**^**H NMR** (600 MHz, CDCl_3_/CD_3_OD 2:1 (v/v)), δ: 0.60–0.65 (m, 18H, CH_3_-20 (A), CH_3_-20 (B), CH_3_-21 (A), CH_3_-21 (B), C**H**_**3**_(CH_2_)_13_CH_2_C(O) (A), C**H**_**3**_(CH_2_)_13_CH_2_C(O) (B)), 0.63 (d, *J* = 6.6 Hz, 12H, CH_3_-16 (A), CH_3_-16 (B), CH_3_-22 (A), CH_3_-22 (B)), 0.88 (s, 6H, CH_3_-19 (A), CH_3_-19 (B)), 0.83–1.13 (four m, 88H, CH_2_-4 (A), CH_2_-4 (B), CH_2_-5 (A), CH_2_-5 (B), CH_2_-6 (A), CH_2_-6 (B), CH_2_-8 (A), CH_2_-8 (B), CH_2_-9 (A), CH_2_-9 (B), CH_2_-10 (A), CH_2_-10 (B), CH_2_-12 (A), CH_2_-12 (B), CH_2_-13 (A), CH_2_-13 (B), CH_2_-14 (A), CH_2_-14 (B), H-7 (A), H-7 (B), H-11 (A), H-11 (B), CH_3_(C**H**_**2**_)_12_CH_2_CH_2_C(O) (A), CH_3_(C**H**_**2**_)_12_CH_2_CH_2_C(O) (B)), 1.29 (m, 2H, H-15 (A), H-15 (B)), 1.36–1.38 (m, 2H, CH_3_(CH_2_)_12_C**H**_**2**_CH_2_C(O) (A), CH_3_(CH_2_)_12_C**H**_**2**_CH_2_C(O) (B)), 2.06–2.15 (two m, 8H, CH_2_-2 (A), CH_2_-2 (B), CH_3_(CH_2_)_13_C**H**_**2**_C(O) (A), CH_3_(CH_2_)_13_C**H**_**2**_C(O) (B)), 2.99 (s, 18H, -N(CH_3_)_3_ (A), -N(CH_3_)_3_ (B)), 3.37–3.41 (m, 4H, CH_2_-β (A), CH_2_-β (B)), 3.75–3.77 (m, 4H, CH_2_-3' (A), CH_2_-3' (B)), 3.88–3.91 (m, 2H, one of CH_2_-1' (A), one of CH_2_-1' (B), 4.00–4.06 (m, 4H, CH_2_-α (A), CH_2_-α (B)), 4.16 (m, 2H, one of CH_2_-1' (A), one of CH_2_-1' (B)), 4.69–4.73 (two d, *J* = 18.0 Hz, 2H, one of CH_2_-18 (A), one of CH_2_-18 (B)), 4.76–4.79 (two d, *J* = 10.2 Hz, 2H, H, one of CH_2_-18 (A), one of CH_2_-18 (B)), 4.97 (m, 2H, H-2' (A), H-2' (B)), 5.55 (m, 2H, H-17 (A), H-17 (B)); ^**13**^**C NMR** (151 MHz, CDCl_3_/CD_3_OD 2:1 (v/v)), δ: 13.51, 13.53 (**C**H_3_(CH_2_)_13_CH_2_C(O) (A), **C**H_3_(CH_2_)_13_CH_2_C(O) (B)), 19.08, 19.14, 19.22, 22.07, 22.16 (C-20 (A), C-20 (B), C-21 (A), C-21 (B), (C-16 (A), C-16 (B), C-22 (A), C-22 (B), (C-19 (A), C-19 (B)), 21.11, 21.13, 22.25, 22.42, 22.44, 22.51, 24.06, 24.39, 24.40, 24.43, 24.47, 28.75, 28.90, 28.94, 29.10, 29.24, 29.28, 36.87, 36.98, 36.99, 37.04, 37.06, 37.28, 37.37, 38.83, 38.84, 38.91, 38.93, 40.93 (C-4 (A), C-4 (B), C-5 (A), C-5 (B), C-6 (A), C-6 (B), C-8 (A), C-8 (B), C-9 (A), C-9 (B), C-10 (A), C-10 (B), C-12 (A), C-12 (B), C-13 (A), C-13 (B), C-14 (A), C-14 (B), CH_3_(**C**H_2_)_12_CH_2_CH_2_C(O) (A), CH_3_(**C**H_2_)_12_CH_2_CH_2_C(O) (B), 27.56 (C-15 (A), C-15 (B)), 31.52, 32.37, 32.38 (C-7 (A), C-7 (B), C-11 (A), C-11 (B)), 33.69 (CH_3_(CH_2_)_13_**C**H_2_C(O) (A), CH_3_(CH_2_)_13_**C**H_2_C(O) (B)), 38.97 (C-3 (A), C-3 (B)), 44.73, 44.79 (C-2 (A), C-2 (B)), 53.73 (-N(**C**H_3_)_3_ (A), -N(**C**H_3_)_3_ (B)), 58.81 (d, *J* = 4.2 Hz, C-α (A), C-α (B)), 62.42 (C-1' (A), C-1’ (B)), 63.34 (d, *J* = 5.3 Hz, C-3' (A), C-3' (B)), 66.03 (m, C-β (A), C-β (B)), 69.80 (d, *J* = 8.0 Hz, C-2'(A), C-2'(B)), 111.71 (C-18 (A), C-18 (B)), 145.02 (C-17 (A), C-17 (B)), 171.06 (C-1 (A), C-1 (B)), 173.59 (CH_3_(CH_2_)_13_CH_2_**C**(O) (A), CH_3_(CH_2_)_13_CH_2_**C**(O) (B)); ^**31**^**P NMR** (243 MHz, CDCl_3_/CD_3_OD 2:1 (v/v)), δ: -0.91; HRMS (ESI): *m/z* calcd. for C_46_H_90_NO_8_P [M + H]^+^ 816.6483; found 816.6480

**1-isoprenoyl-2-hydroxy-*sn*-glycero-3’-phosphatidylcholines**

**(2’*R*)-1’-[(3*R*)-3,7-dimethyl-3-vinylocta-6-enyl]*-*2’-hydroxy*-sn*-glycero-3’-phosphocholine + (2’*R*)-1’-[(3*S*)-3,7-dimethyl-3-vinylocta-6-enyl]*-*2’-hydroxy*-sn*-glycero-3’-phosphocholine (8a)**

mixture of two diasteroisomers, colourless greasy solid (32% yield, 98% purity (according to HPLC); ^**1**^**H NMR** (600 MHz, CDCl_3_/CD_3_OD 2:1 (v/v)), δ: 0.91 (s, 6H, CH_3_-11 (A), CH_3_-11 (B)), 1.17–1.21 (m, 4H, CH_2_-4 (a), CH_2_-4 (B)), 1.35, 1.44 (two s, 12H, CH_3_-8 (A), CH_3_-8 (B), CH_3_-12 (A), CH_3_-12 (B)), 1.68 (m, 4H, CH_2_-5 (A), CH_2_-5 (B)), 2.12–2.17 (two d, *J* = 13.8 Hz, 4H, AB system, CH_2_-2 (A), CH_2_-2 (B)), 2.99 (s, 18H, -N(CH_3_)_3_ (A), -N(CH_3_)_3_ (B)), 3.38 (m, 4H, CH_2_-β (A), CH_2_-β (B)), 3.63–3.66 (m, 2H, one of CH_2_-3' (A), one of CH_2_-3' (B)), 3.66–3.73 (m, 4H, H-2' (A), H-2’ (B), one of CH_2_-3' (A), one of CH_2_-3' (B)), 3.83–3.91 (two m, 4H, CH_2_-1' (A), CH_2_-1' (B)), 4.01–4.06 (broad m, 4H, CH_2_-α (A), CH_2_-α (B)), 4.74 (d, *J* = 18.0 Hz, 2H, one of CH_2_-10 (A), one of CH_2_-10 (B)), 4.81 (d, *J* = 10.8 Hz, 2H, one of CH_2_-10 (A), one of CH_2_-10 (B)) 4.84 (t, *J* = 7.2 Hz, 2H, H-6 (A), H-6 (B)), 5.92 (dd, *J* = 18.0, 10.8 Hz, 2H, H-9 (A), H-9 (B)); ^**13**^**C NMR** (151 MHz, CDCl_3_/CD_3_OD 2:1 (v/v)) δ: 16.86, 24.93 (C-8 (A), C-8 (B), C-12 (A), C-12 (B)), 22.40 (C-11 (A), C-11 (B)), 22.43 (C-5 (A), C-5 (B)), 38.75 (C-3 (A), C-3 (B)), 40.35, 40.38 (C-4 (A), C-4 (B)), 44.48, 44.50 (C-2 (a), C-2 (B)), 53.67 (t, *J* = 3.6 Hz, -N(**C**H_3_)_3_ (A), -N(**C**H_3_)_3_ (B)), 58.64 (d, *J* = 4.9 Hz, C-α (A), C-α (B)), 64.37 (C-1' (A), C-1' (B)), 66.09 (m, C-β (A), C-β (B)), 66.55 (d, *J* = 5.7 Hz, C-3' (A), C-3’ (B)), 68.36 (d, *J* = 6.5 Hz, C-2' (A), C-2’ (B)), 111.71 (C-10 (A), C-10 (B)), 123.93 (C-6 (A), C-6 (B)), 130.95 (C-7 (A), C-7 (B)), 144.94 (C-9 (A), C-9 (B)), 171.75 (C-1 (A), C-1 (B)); ^**31**^**P NMR** (243 MHz, CDCl_3_/CD_3_OD 2:1 (v/v)) δ: -0.04; HRMS (ESI): *m/z* calcd. for C_18_H_34_NO_7_P [M + H]^+^ 408.2151; found 408.2153

**(2'R)-1’-[(3R,6E)-3,7,11-trimethyl-3-vinyldodec-6,10-dienyl]-2’-hydroxy-sn-glycero-3’-phosphocholine + (2'R)-1’-[(3S,6E)-3,7,11-trimethyl-3-vinyldodec-6,10-dienyl]-2’-hydroxy-sn-glycero-3’-phosphocholine (8b)**

mixture of two diasteroisomers, colourless greasy solid (52% yield, 97% purity (according to HPLC); ^**1**^**H NMR** (600 MHz, CDCl_3_/CD_3_OD 2:1 (v/v)), δ: 0.91 (s, 6H, CH_3_-15 (A), CH_3_-15 (B)), 1.18–1.18–1.21 (m, 4H, CH_2_-4 (A), CH_2_-4 (B)), 1.35, 1.44 (two s, 12H, CH_3_-12 (A), CH_3_-12 (B), CH_3_-17 (A), CH_3_-17 (B)), 1.36 (s, 3H, CH_3_-16 (A), CH_3_-16 (B)), 1.67–1.71 (m, 4H, CH_2_-5 (A), CH_2_-5 (B)), 1.72–1.74 (m, 2H, CH_2_-8 (A), CH_2_-8 (B)), 1.80–1.84 (m, 2H, CH_2_-9 (A), CH_2_-9 (B)), 2.12–2.18 (two d, *J* = 14.4 Hz, 4H, AB system, CH_2_-2 (A), CH_2_-2 (B)), 2.98 (s, 18H, -N(CH_3_)_3_ (A), -N(CH_3_)_3_ (B)), 3.38 (m, 4H, CH_2_-β (A), CH_2_-β (A)), 3.61–3.66 (m, 2H, one of CH_2_-3' (A), one of CH_2_-3' (B)), 3.70–3.75 (two m, 4H, one of CH_2_-3' (A), one of CH_2_-3' (B), H-2' (A), H-2' (B)), 3.84–3.90 (two m, 4H, CH_2_-1' (A), CH_2_-1' (B)), 4.01–4.06 (broad m, 4H, CH_2_-α (A), CH_2_-α (B)), 4.74 (d, *J* = 17.4 Hz, 2H, one of CH_2_-14 (A), one of CH_2_-14 (B)), 4.80 (d, *J* = 10.8 Hz, 2H, one of CH_2_-10 (A), one of CH_2_-10 (B)), 4.84–4.86 (m, 4H, H-6 (A), H-6 (B), H-10 (A), H-10 (B)), 5.59 (dd, *J* = 17.4, 10.8 Hz, 2H, H-13 (A), H-13 (B)); ^**13**^**C NMR** (151 MHz, CDCl_3_/CD_3_OD 2:1 (v/v)) δ: 15.22, 24.97 (C-12 (A), C-12 (B), C-17 (A), C-17 (B)), 16.95 (C-16 (A), C-16 (B)), 22.25 (C-5 (A), C-5 (B)), 22.27 (C-15 (A), C-15 (B)), 26.21 (C-9 (A), C-9 (B)), 38.77 (C-3 (A), C-3 (B)), 39.22 (C-8), 40.43, 40.46 (C-4 (A), C-4 (B)), 44.49, 44.52 (C-2 (A), C-2 (B)), 53.60 (t, *J* = 3.8 Hz, -N(**C**H_3_)_3_ (A), -N(**C**H_3_)_3_ (B)), 58.61, 58.65 (d, *J* = 4.8 Hz, C-α (A), C-α (B)), 64.31, 64.32 (C-1' (A), C-1' (B)), 66.00 (m, C-β (A), C-β (B)), 66.51 (d, *J* = 5.4 Hz, C-3' (A), C-3' (B)), 68.28 (d, *J* = 6.5 Hz, C-2' (A), C-2' (B)), 111.75 (C-14 (A), C-14 (B)), 123.78 (C-10 (A), C-10 (B)), 123.84 (C-6 (A), C-6 (B)), 130.80 (C-11 (A), C-11 (B)), 134.61 (C-7 (A), C-7 (B)), 144.87 (C-13 (A), C-13 (B)), 171.75 (C-1 (A), C-1 (B)); ^**31**^**P NMR** (243 MHz, CDCl_3_/CD_3_OD 2:1 (v/v)) δ: 0.00; HRMS (ESI): *m/z* calcd. for C_25_H_46_NO_7_P [M + H]^+^ 504.3090; found 504.3102

**(2’*R*)-1’-{2-[(3*R*,2"*E*)-2"-butylidene-1",3",3"-trimethyl]cyclohexyl}acetyl*-*2’-hydroxy*-sn*-glycero-3’-phospocholine + (2’*R*)-1’-{2-[(3*S*,2"*E*)-2"-butylidene-1",3",3"-trimethyl]cyclohexyl}acetyl*-*2’-hydroxy-*sn*-glycero-3’-phospocholine (8c)**

mixture of two diasteroisomers, colourless greasy solid (24% yield, 99% purity (according to HPLC); ^**1**^**H NMR** (600 MHz, CDCl_3_/CD_3_OD 2:1 (v/v)), δ: 0.68 (t, *J* = 7.8 Hz, 6H, CH_3_-10 (A), CH_3_-10 (B)), 0.97 (s, 12H, CH_3_-11 (A), CH_3_-11 (B), CH_3_-12 (A), CH_3_-12 (B)), 0.98 (s, 6H, CH_3_-13 (A), CH_3_-13 (B)), 1.11–1.19 (m, 8H, CH_2_-9 (A), CH_2_-9 (B), one of CH_2_-5 (A), one of CH_2_-5 (B), one of CH_2_-6 (A), one of CH_2_-6 (B)), 1.32–1.40 (m, 6H, CH_2_-4 (A), CH_2_-4 (B), one of CH_2_-5 (A), one of CH_2_-5 (B)), 1.52–1.56 (m, 2H, one of CH_2_-6 (A), one of CH_2_-6 (B)), 1.91–1.96 (m, 4H, CH_2_-8 (A), CH_2_-8 (B)), 2.18–2.37 (d, *J* = 13.8 Hz, AB system, 4H, CH_2_-14 (A), CH_2_-14 (B)), 2.98 (s, 18H, -N(CH_3_)_3_ (A), -N(CH_3_)_3_ (B)), 3.35–3.40 (broad s, 4H, CH_2_-β (A), CH_2_-β (B)), 3.61–3.65 (m, 2H, one of CH_2_-3' (A), one of CH_2_-3’ (B)), 3.71–3.73 (m, 2H, one of CH_2_-3' (A), one of CH_2_-3' (B), H-2' (A), H-2' (B)), 3.79–3.88 (m, 4H, CH_2_-1' (A), CH_2_-1' (B)), 4.01–4.06 (broad s, 4H, CH_2_-α (A), CH_2_-α (B)), 4.99 (t, *J* = 6.6 Hz, 2H, H-7 (A), H-7 (B)); ^**13**^**C NMR** (151 MHz, CDCl_3_/CD_3_OD 2:1 (v/v)) δ: 13.21 (C-10 (A), C-10 (B)), 17.06 (C-4 (A), C-4 (B)), 23.23 (C-9 (A), C-9 (B)), 29.95, 30.12, 30.91, 30.93 (C-11 (A), C-11 (B), C-12 (A), C-12 (B), C-13 (A), C-13 (B)), 31.84 (C-8 (A), C-8 (B)), 34.86 (C-3 (A), C-3 (B)), 35.61, 35.62 (C-6 (A), C-6 (B)), 39.14 (C-1 (A), C-1 (B)), 40.78 (C-5 (A), C-5 (B)), 47.14 (C-14 (A), C-14 (B)), 53.65 (t, *J* = 3.5 Hz, -N(**C**H_3_)_3_ (A), -N(**C**H_3_)_3_ (B)), 58.67 (d, *J* = 4.8 Hz, C-α (A), C-α (B)), 64.20, 64.22 (C-1' (A), C-1’ (B)), 66.06 (m, C-β (A), C-β (B)), 66.63 (d, *J* = 5.4 Hz, C-3' (A), C-3' (B)) 68.42 (d, *J* = 6.5 Hz, C-2' (A), C-2' (B)), 126.48, 126.51 (C-7 (A), C-7 (B)), 148.90, 148.94 (C-2 (A), C-2 (B)), 172.16 (C-15 (A), C-15 (B)); ^**31**^**P NMR** (243 MHz, CDCl_3_/CD_3_OD 2:1 (v/v)) δ: -0.06; HRMS (ESI): *m/z* calcd. for C_23_H_44_NO_7_P [M + H]^+^ 478.2933; found 478.2935

**(2'*R*)-1’-[(3*R*,7*R*,11*R*)-3,7,11,15-tetramethyl-3-vinylhexadecyl]-2’-hydroxy-*sn*-glycero-3’-phosphocholine + (2'*R*)-1’-[(3*S*,7*R*,11*R*)-3,7,11,15-tetramethyl-3-vinylhexadecyl]-2’-hydroxy-*sn*-glycero-3’-phosphocholine (8d)**

mixture of two diasteroisomers, colourless greasy solid (34% yield, 99% purity (according to HPLC); ^**1**^**H NMR** (600 MHz, CDCl_3_/CD_3_OD 2:1 (v/v)), δ: 0.60. 0.61 (two d, *J* = 5.4 Hz, 12H, CH_3_-20 (A), CH_3_-20 (B), CH_3_-21 (A), CH_3_-21 (B)), 0.63 (d, *J* = 6.6 Hz, 12H, CH_3_-16 (A), CH_3_-16 (B), CH_3_-22 (a), CH_3_-22 (B)), 0.89 (s, 6H, CH_3_-19 (A), CH_3_-19 (B)), 0.89–1.16 (four m, 40H, CH_2_-4 (A), CH_2_-4 (B), CH_2_-5 (A), CH_2_-5 (B), CH_2_-6 (A), CH_2_-6 (B), CH_2_-8 (A), CH_2_-8 (B), CH_2_-9 (A), CH_2_-9 (B), CH_2_-10 (A), CH_2_-10 (B), CH_2_-12 (A), CH_2_-12 (B), CH_2_-13 (A), CH_2_-13 (B), CH_2_-14 (A), CH_2_-14 (B), H-7 (A), H-7 (B), H-11 (A), H-11 (B)), 1.29 (m, 2H, H-15 (A), H-15 (B)), 2.09–2.16 (two d, *J* = 13.8 Hz, 4H, CH_2_-2 (A), CH_2_-2 (B)), 2.98 (s, 18H, -N(CH_3_)_3_ (A), -N(CH_3_)_3_ (B)), 3.37 (m, 4H, CH_2_-β (A), CH_2_-β (B)), 3.61–3.65 (m, 2H, one of CH_2_-3' (A), one of CH_2_-3' (B)), 3.69–3.73 (two m, 4H, one of CH_2_-3' (A), one of CH_2_-3' (B), H-2' (A), H-2' (B)), 3.82–3.89 (two m, 4H, CH_2_-1' (A), CH_2_-1' (B)), 4.03 (m, 4H, CH_2_-α (A), CH_2_-α (B)), 4.71 (d, *J* = 17.4 Hz, 2H, one of CH_2_-18 (A), one of CH_2_-18 (B)), 4.78 (d, *J* = 10.8 Hz, 2H, one of CH_2_-18 (A), one of CH_2_-18 (B)), 5.57 (dd, *J* = 17.4, 10.8 Hz, 2H, H-17 (A), H-17 (B)); ^**13**^**C NMR** (151 MHz, CDCl_3_/CD_3_OD 2:1 (v/v)) δ: 18.99, 19.06, 19.13 (C-20 (A), C-20 (B), C-21 (A), C-21 (B)), 21.01, 21.03, 21.05, 23.97, 24.32, 24.33, 29.17, 36.80, 36.91, 36.93, 36.94, 36.95, 36.97, 37.17, 37.25, 38.91, 40.91, 40.92, 40.94, 40.96 (C-4 (A), C-4 (B), C-5 (A), C-5 (B), C-6 (A), C-6 (B), C-8 (A), C-8 (B), C-9 (A), C-9 (B), C-10 (A), C-10 (B), C-12 (A), C-12 (B), C-13 (A), C-13 (B), C-14 (A), C-14 (B), 21.97, 22.07 (C-16 (A), C-16 (B), C-22 (A), C-22 (B)), 22.26, 22.31 (C-19 (A), C-19 (B)), 27.50 (C-15 (A), C-15 (B)), 32.25, 32.27, 32.28, 32.29, 32.31 (C-7 (A), C-7 (B), C-11 (A), C-11 (B)), 38.82 (C-3), 44.68, 44.70 (C-2 (A), C-2 (B)), 53.61 (t, *J* = 3.8 Hz, -N(**C**H_3_)_3_ (A), -N(**C**H_3_)_3_ (B)), 58.62 (d, *J* = 5.1 Hz, C-α (A), C-α (B)), 64.29, 64.30 (C-1' (A), C-1’ (B)), 66.00 (m, C-β (A), C-β (B)), 66.52 (d, *J* = 5.7 Hz, C-3' (A), C-3' (B)), 68.28 (d, *J* = 7.0 Hz, C-2' (A), 68.29 (d, *J* = 6.8 Hz, C-2' (B)), 111.55 (C-18 (A), C-18 (B)), 145.10 (C-17 (A), C-17 (B)), 171.85 (C-1 (A), C-1 (B)); ^**31**^**P NMR** (243 MHz, CDCl_3_/CD_3_OD 2:1 (v/v)) δ: 0.04; HRMS (ESI): *m/z* calcd. for C_30_H_60_NO_7_P [M + H]^+^ 578.4186; found 578.4198

**1-isoprenoyl-2-palmitoyl-*sn*-glycero-3’-phosphatidylcholines**

**(2’*R*)-1’-[(3*R*)-3,7-dimethyl-3-vinylocta-6-enyl]*-*2’-palmitoyl-*sn*-glycero-3’-phosphocholine + (2’*R*)-1’-[(3*S*)-3,7-dimethyl-3-vinylocta-6-enyl]*-*2’-palmitoyl-*sn*-glycero-3’-phosphocholine (9a)**

mixture of two diasteroisomers, colourless greasy solid (68% yield, 98% purity (according to HPLC);^**1**^**H NMR** (600 MHz, CDCl_3_/CD_3_OD 2:1 (v/v)), δ: 0.68 (t, *J* = 6.6 Hz, 6H, C**H**_**3**_(CH_2_)_14_C(O) (A), C**H**_**3**_(CH_2_)_14_C(O) (B)), 0.92 (s, 6H, CH_3_-11 (A), CH_3_-11 (B)), 1.06–1.09 (m, 48H, CH_3_(C**H**_**2**_)_**12**_CH_2_CH_2_C(O) (A), CH_3_(C**H**_**2**_)_**12**_CH_2_CH_2_C(O) (B)), 1.14–1.22 (m, 4H, CH_2_-4 (A), CH_2_-4 (B)), 1.38, 1.47 (two s, 12H, CH_3_-8 (A), CH_3_-8 (B), CH_3_-12 (A), CH_3_-12 (B)), 1.40–1.42 (m, 4H, CH_3_(CH_2_)_13_C**H**_**2**_CH_2_C(O) (A), CH_3_(CH_2_)_13_C**H**_**2**_CH_2_C(O) (B)), 1.70 (m, 4H, CH_2_-5 (A), CH_2_-5 (B)), 2.10–2.18 (m, 8H, CH_3_(CH_2_)_13_C**H**_**2**_C(O) (A), CH_3_(CH_2_)_13_C**H**_**2**_C(O) (B), CH_2_-2 (A), CH_2_-2 (B)), 3.02 (s, 18H, -N(CH_3_)_3_ (A), -N(CH_3_)_3_ (B)), 3.43 (m, 4H, CH_2_-β (A), CH_2_-β (B)), 3.80 (m, 4H, CH_2_-3' (A), CH_2_-3' (B)), 3.93 (dd, *J* = 12.0, 6.6 Hz, 2H, one of CH_2_-1' (A), one of CH_2_-1' (B)), 4.06–4.08 (broad s, 4H, CH_2_-α (A), CH_2_-α (B)), 4.18 (m, 2H, one of CH_2_-1' (A), one of CH_2_-1' (B)), 4.76 (d, *J* = 17.4, 2H, one of CH_2_-10 (A), one of CH_2_-10 (B)), 4.83 (d, *J* = 10.8 Hz, 2H, one of CH_2_-10 (A), one of CH_2_-10 (B)), 4.86 (t, *J* = 6.6 Hz, 2H, H-6 (A), H-6 (B)), 4.98–5.03 (m, 2H, H-2' (A), H-2' (B)), 5.59 (dd, *J* = 17.4, 10.8 Hz, 2H, H-9 (A), H-9 (B)); ^**13**^**C NMR** (151 MHz, CDCl_3_/CD_3_OD 2:1 (v/v)) δ: 13.54 (**C**H_3_(CH_2_)_14_C(O) (A), (**C**H_3_(CH_2_)_14_C(O) (B)), 17.01, 25.09 (C-8 (A), C-8 (B), C-12 (A), C-12 (B)), 22.26 (CH_3_**C**H_2_(CH_2_)_13_C(O) (A), CH_3_**C**H_2_(CH_2_)_13_C(O) (B)), 22.44 (C-5 (A), C-5 (B)), 22.51, 22.53 (C-11 (A), C-11 (B)), 24.47 (CH_3_(CH_2_)_12_**C**H_2_CH_2_C(O) (A), CH_3_(CH_2_)_12_**C**H_2_CH_2_C(O) (B)), 28.74, 28.93, 28.95, 29.12, 29.25, 29.27, 29.29, 31.52 (CH_3_CH_2_(**C**H_2_)_11_CH_2_CH_2_C(O) (A), CH_3_CH_2_(**C**H_2_)_11_CH_2_CH_2_C(O) (B)), 33.83 (CH_3_(CH_2_)_13_**C**H_2_C(O) (A), CH_3_(CH_2_)_13_**C**H_2_C(O) (B)), 38.79 (C-3 (A), C-3 (B)), 40.37, 40.39 (C-4 (A), C-4 (B)), 44.50, 44.52 (C-2 (A), C-2 (B)), 53.74 (t, *J* = 3.6 Hz, -N(**C**H_3_)_3_ (A), -N(**C**H_3_)_3_ (B)), 58.84 (d, *J* = 4.9 Hz, C-α (A), C-α (B)), 63.00 (C-1' (A), C-1' (B)), 63.41 (d, *J* = 5.3 Hz, C-3' (A), C-3' (B)), 66.03 (m, C-β (A), C-β (B)), 69.99 (d, *J* = 7.5 Hz, C-2' (A)), 70.00 (d, *J* = 7.8 Hz, C-2' (B)), 111.90 (C-10 (A), C-10 (B)), 123.89 (C-6 (A), C-6 (B)), 131.09 (C-7 (A), C-7 (B)), 144.81 (C-9 (A), C-9 (B)), 171.47 (C-1 (A), C-1 (B)), 173.27 (CH_3_(CH_2_)_13_CH_2_**C**(O) (A), CH_3_(CH_2_)_13_CH_2_**C**(O) (B)); ^**31**^**P NMR** (243 MHz, CDCl_3_/CD_3_OD 2:1 (v/v)) δ: -0.83; HRMS (ESI): *m/z* calcd. for C_36_H_68_NO_8_P [M + H]^+^ 674.4761; found 674.4769

**(2'*R*)-1’-[(3*R*,6*E*)-3,7,11-trimethyl-3-vinyldodeca-6,10-dienyl]*-*2’-palmitoyl-*sn*-glycero-3’-phosphocholine + (2'*R*)-1’-[(3*S*,6*E*)-3,7,11-trimethyl-3-vinyldodeca-6,10-dienyl]-2’-palmitoyl-*sn*-glycero-3’-phosphocholine (9b)**

mixture of two diasteroisomers, colourless greasy solid (64% yield, 99% purity (according to HPLC); ^**1**^**H NMR** (600 MHz, CDCl_3_/CD_3_OD 2:1 (v/v)), δ: 0.64 (t, *J* = 6.6 Hz, 6H, C**H**_**3**_(CH_2_)_13_CH_2_C(O) (A), C**H**_**3**_(CH_2_)_13_CH_2_C(O) (B)), 0.89 (s, 6H, CH_3_-15 (A), CH_3_-15 (B)), 1.02–1.05 (m, 48H, CH_3_(C**H**_**2**_)_12_CH_2_CH_2_C(O) (A), CH_3_(C**H**_**2**_)_12_CH_2_CH_2_C(O) (B)), 1.16–1.19 (m, 4H, CH_2_-4 (A), CH_2_-4 (B)), 1.34, 1.43 (two s, 12H, CH_3_-12 (A), CH_3_-12 (B), CH_3_-17 (A), CH_3_-17 (B)), 1.35–1.40 (m, 4H, CH_3_(CH_2_)_12_C**H**_**2**_CH_2_C(O) (A), CH_3_(CH_2_)_12_C**H**_**2**_CH_2_C(O) (B)), 1.36 (s, 6H, CH_3_-16 (A), CH_3_-16 (B)), 1.66–1.73 (two m, 8H, CH_2_-5 (A), CH_2_-5 (B), CH_2_-8 (A), CH_2_-8 (B)), 1.80–1.83 (m, 4H, CH_2_-9 (A), CH_2_-9 (B)), 2.06–2.14 (m, 4H, CH_2_-2 (A), CH_2_-2 (B)), 2.09 (t, *J* = 7.8 Hz, 4H, CH_3_(CH_2_)_13_C**H**_**2**_C(O) (A), CH_3_(CH_2_)_13_C**H**_**2**_C(O) (B)), 2.99 (s, 18H, -N(CH_3_)_3_ (A), -N(CH_3_)_3_ (B)), 3.37–3.40 (broad s, 4H, CH_2_-β (A), CH_2_-β (B)), 3.76–3.78 (m, 4H, CH_2_-3' (A), CH_2_-3' (B)), 3.88–3.91 (dd, *J* = 12.0, 6.6 Hz, 2H, one of CH_2_-1' (A), one of CH_2_-1' (B)), 4.00–4.05 (broad s, 4H, CH_2_-α (A), CH_2_-α (B)), 4.15 (m, 2H, one of CH_2_-1' (A), one of CH_2_-1' (B)), 4.72 (d, *J* = 17.4, 2H, one of CH_2_-14 (A), one of CH_2_-14 (B)), 4.79 (d, *J* = 10.8 Hz, 2H, one of CH_2_-14 (A), one of CH_2_-14 (B)), 4.83–4.85 (m, 4H, H-6 (A), H-6 (B), H-10 (A), H-10 (B)), 4.95–4.98 (m, 2H, H-2' (A), H-2' (B)), 5.56 (dd, *J* = 17.4, 10.8 Hz, 2H, H-13 (A), H-13 (B)); ^**13**^**C NMR** (151 MHz, CDCl_3_/CD_3_OD 2:1 (v/v)) δ: 13.58 (**C**H_3_(CH_2_)_13_CH_2_C(O) (A), **C**H_3_(CH_2_)_13_CH_2_C(O) (B)), 15.41, 25.15 ((C-12 (A), C-12 (B), C-17 (A), C-17 (B)), 17.13 (C-16 (A), C-16 (B)), 22.30 (CH_3_**C**H_2_(CH_2_)_12_CH_2_C(O) (A), CH_3_**C**H_2_(CH_2_)_12_CH_2_C(O) (B)), 22.37 (C-5 (A), C-5 (B)), 22.50, 22.52 (C-15 (A), C-15 (B)), 24.51 (CH_3_(CH_2_)_12_**C**H_2_CH_2_C(O) (A), CH_3_(CH_2_)_12_**C**H_2_CH_2_C(O) (B)), 26.33 (C-9 (A), C-9 (B)), 28.77, 28.97, 28.98, 29.15, 29.28, 29.30, 29.32, 31.56 (CH_3_CH_2_(**C**H_2_)_11_CH_2_CH_2_C(O) (A), CH_3_CH_2_(**C**H_2_)_11_CH_2_CH_2_C(O) (B)), 33.87 CH_3_(CH_2_)_13_**C**H_2_C(O) (A), CH_3_(CH_2_)_13_**C**H_2_C(O) (B)), 38.84 (C-3 (A), C-3 (B)), 39.34 (C-8 (A), C-8 (B)), 40.49, 40.51 (C-4 (A), C-4 (B)), 44.55, 44.58 (C-2 (A), C-2 (B)), 53.79 (t, *J* = 3.6 Hz, -N(**C**H_3_)_3_ (A), -N(**C**H_3_)_3_ (B)), 58.78 (d, *J* = 4.9 Hz, C-α (A), C-α (B)), 62.12 (C-1' (A), C-1' (B)), 63.42 (d, *J* = 5.1 Hz, C-3' (A), C-3' (B)) 66.10 (m, C-β (A), C-β (B)), 70.02 (d, *J* = 7.7 Hz, C-2' (A)), 70.03 (d, *J* = 8.0 Hz, C-2' (B)), 111.94 (C-14 (A), C-14 (B)), 123.79 (C-10 (A), C-10 (B)), 123.93 (C-6 (A), C-6 (B)), 130.94 (C-11 (A), C-11 (B)), 134.79 (C-7 (A), C-7 (B)), 144.84 (C-13 (A), C-13 (B)), 171.43 (C-1 (A), C-1 (B)), 173.22 (CH_3_(CH_2_)_13_CH_2_**C**(O) (A), CH_3_(CH_2_)_13_CH_2_**C**(O) (B)); ^**31**^**P NMR** (243 MHz, CDCl_3_/CD_3_OD 2:1 (v/v)) δ: - 0.82; HRMS (ESI): *m/z* calcd. for C_41_H_76_NO_8_P [M + H]^+^ 742.5387; found 742.5392

**(2’*R*)-1’-{2-[(3*R*,2"*E*)-2"-butylidene-1",3",3"-trimethyl]cyclohexyl}acetyl*-*2’-palmitoyl-*sn*-glycero-3’-phospocholine + (2’*R*)-1’-{2-[(3*S*,2"*E*)-2"-butylidene-1",3",3"-trimethyl]cyclohexyl}acetyl*-*2’-palmitoyl-*sn*-glycero-3’-phospocholine (9c)**

mixture of two diasteroisomers, colourless greasy solid (47% yield, 99% purity (according to HPLC); ^**1**^**H NMR** (600 MHz, CDCl_3_/CD_3_OD 2:1 (v/v)), δ: 0.64 (t, *J* = 6.6 Hz, 6H, C**H**_**3**_(CH_2_)_13_CH_2_C(O) (A), C**H**_**3**_(CH_2_)_13_CH_2_C(O) (B)), 0.67 (t, *J* = 7.2 Hz, 6H, CH_3_-10 (A), CH_3_-10 (B)), 0.96 (s, 12H, CH_3_-11 (A), CH_3_-11 (B), CH_3_-12 (A), CH_3_-12 (B), 1.02–1.05 (m, 54H, CH_3_(C**H**_**2**_)_12_CH_2_CH_2_C(O) (A), CH_3_(C**H**_**2**_)_12_CH_2_CH_2_C(O) (B), CH_3_-13 (A), CH_3_-13 (B)), 1.09–1.16 (m, 8H, CH_2_-9 (A), CH_2_-9 (B), one of CH_2_-5 (A), one of CH_2_-5 (B), one of CH_2_-6 (A), one of CH_2_-6 (B)), 1.31–1.37 (m, 10H, CH_2_-4 (A), CH_2_-4 (B), CH_3_(CH_2_)_12_C**H**_**2**_CH_2_C(O) (A), CH_3_(CH_2_)_12_C**H**_**2**_CH_2_C(O) (B), one of CH_2_-5 (A), one of CH_2_-5 (B)), 1.47–1.51 (m, 2H, one of CH_2_-6 (A), one of CH_2_-6 (B)), 1.91–1.95 (m, 4H, CH_2_-8 (A), CH_2_-8 (B)), 2.09 (t, *J* = 7.8 Hz, 4H, CH_3_(CH_2_)_13_C**H**_**2**_C(O) (A), CH_3_(CH_2_)_13_C**H**_**2**_C(O) (B)), 2.08–2.34 (four d, *J* = 13.8 Hz, two AB systems, 4H, CH_2_-14 (A), CH_2_-14 (B)), 2.99 (s, 18H, -N(CH_3_)_3_ (A), -N(CH_3_)_3_ (B)), 3.39–3.43 (m, 4H, CH_2_-β (A), CH_2_-β (B)), 3.78–3.80 (m, 4H, CH_2_-3' (A), CH_2_-3' (B)), 3.84–3.91 (two dd, *J* = 12.0, 6.6 Hz, 2H, one of CH_2_-1' (A), one of CH_2_-1' (B)), 4.03–4.08 (broad s, 4H, CH_2_-α (A), CH_2_-α (B)), 4.08–4.13 (two m, 2H, one of CH_2_-1' (A), one of CH_2_-1' (B)), 4.97–4.99 (m, 4H, H-2' (A), H-2' (B), H-7 (A), H-7 (B)); ^**13**^**C NMR** (151 MHz, CDCl_3_/CD_3_OD 2:1 (v/v)) δ: 13.39 (**C**H_3_(CH_2_)_13_CH_2_C(O) (A), **C**H_3_(CH_2_)_13_CH_2_C(O) (B)), 13.55 (C-10 (A), C-10 (B)), 17.11, 17.13 (C-4 (A), C-4 (B)), 22.27 (CH_3_**C**H_2_(CH_2_)_12_CH_2_C(O) (A), CH_3_**C**H_2_(CH_2_)_12_CH_2_C(O) (B)), 23.36 (C-9 (A), C-9 (B)), 24.48 (CH_3_(CH_2_)_12_**C**H_2_CH_2_C(O) (A), CH_3_(CH_2_)_12_**C**H_2_CH_2_C(O) (B)), 28.74, 28.93, 28.96 29.11, 29.26, 29.23, 29.30 (CH_3_CH_2_(**C**H_2_)_11_CH_2_CH_2_C(O) (A), CH_3_CH_2_(**C**H_2_)_11_CH_2_CH_2_C(O) (B)), 29.72, 30.07, 30.23, 30.92, 31.01 (C-11 (A), C-11 (B), C-12 (A), C-12 (B), C-13 (A), C-13 (B)), 31.53 (C-8 (A), C-8 (B)), 33.84 (CH_3_(CH_2_)_13_**C**H_2_C(O) (A), CH_3_(CH_2_)_13_**C**H_2_C(O) (B)), 34.93 (C-3 (A), C-3 (B)), 35.67, 35.70 (C-6 (A), C-6 (B)), 39.18 (C-1 (A), C-1 (B)), 40.80 (C-5 (A), C-5 (B)), 47.14, 47.17 (C-14 (A), C-14 (B)), 53.75 (t, *J* = 3.5 Hz, -N(**C**H_3_)_3_ (A), -N(**C**H_3_)_3_ (B)), 59.02 (d, *J* = 4.8 Hz, C-α (A), C-α (B)), 61.87, 61.92 (C-1' (A), C-1' (B)), 63.71 (d, *J* = 4.8 Hz, C-3' (A), C-3' (B)), 65.97 (m, C-β (A), C-β (B)), 69.97 (m, C-2' (A), C-2' (B)), 126.58, 126.61 (C-7 (A), C-7 (B)), 148.84, 148.87 (C-2 (A), C-2 (B)), 171.79, 171.80 (C-15 (A), C-15 (B)), 173.23 (CH_3_(CH_2_)_13_CH_2_**C**(O) (A), CH_3_(CH_2_)_13_CH_2_**C**(O) (B)); ^**31**^**P NMR** (243 MHz, CDCl_3_/CD_3_OD 2:1 (v/v)) δ: -1.04; HRMS (ESI): *m/z* calcd. for C_39_H_74_NO_8_P [M + H]^+^ 716.5230; found 716.5234

**(2'*R*)-1’-[(3*R*,7*R*,11*R*)-3,7,11,15-tetramethyl-3-vinylhexadecyl]-2’-palmitoyl-*sn*-glycero-3’-phosphocholine + (2'*R*)-1’-[(3*S*,7*R*,11*R*)-3,7,11,15-tetramethyl-3-vinylhexadecyl]-2’-palmitoyl-*sn*-glycero-3’-phosphocholine (9d)**

mixture of two diasteroisomers, colourless greasy solid (65% yield, 99% purity (according to HPLC); ^**1**^**H NMR** (600 MHz, CDCl_3_/CD_3_OD 2:1 (v/v)), δ: 0.60–0.64 (m, 18H, CH_3_-20 (A), CH_3_-20 (B), CH_3_-21 (A), CH_3_-21 (B), C**H**_**3**_(CH_2_)_13_CH_2_C(O) (A), C**H**_**3**_(CH_2_)_13_CH_2_C(O) (B)), 0.63 (d, *J* = 6.0 Hz, 12H, CH_3_-16 (A), CH_3_-16 (B), CH_3_-22 (A), CH_3_-22 (B)), 0.86 (s, 6H, CH_3_-19 (A), CH_3_-19 (B)), 0.82–1.15 (four m, 88H, CH_2_-4 (A), CH_2_-4 (B), CH_2_-5 (A), CH_2_-5 (B), CH_2_-6 (A), CH_2_-6 (B), CH_2_-8 (A), CH_2_-8 (B), CH_2_-9 (A), CH_2_-9 (B), CH_2_-10 (A), CH_2_-10 (B), CH_2_-12 (A), CH_2_-12 (B), CH_2_-13 (A), CH_2_-13 (B), CH_2_-14 (A), CH_2_-14 (B), H-7 (A), H-7 (B), H-11 (A), H-11 (B), CH_3_(C**H**_**2**_)_12_CH_2_CH_2_C(O) (A), CH_3_(C**H**_**2**_)_12_CH_2_CH_2_C(O) (B)), 1.28 (m, 2H, H-15 (A), H-15 (B)), 1.35–1.37 (m, 2H, CH_3_(CH_2_)_12_C**H**_**2**_CH_2_C(O) (A), CH_3_(CH_2_)_12_C**H**_**2**_CH_2_C(O) (B)), 2.06–2.12 (two m, 8H, CH_2_-2 (A), CH_2_-2 (B), CH_3_(CH_2_)_13_C**H**_**2**_C(O) (A), CH_3_(CH_2_)_13_C**H**_**2**_C(O) (B)), 2.98 (s, 18H, -N(CH_3_)_3_ (A), -N(CH_3_)_3_ (B)), 3.39 (m, 4H, CH_2_-β (A), CH_2_-β (B)), 3.77 (m, 4H, CH_2_-3' (A), CH_2_-3' (B)), 3.88 (m, 2H, one of CH_2_-1' (A), one of CH_2_-1' (B), 4.02–4.07 (broad s, 4H, CH_2_-α (A), CH_2_-α (B)), 4.13 (m, 2H, one of CH_2_-1' (A), one of CH_2_-1' (B)), 4.69 (d, *J* = 17.4 Hz, 2H, one of CH_2_-18 (A), one of CH_2_-18 (B)), 4.76 (d, *J* = 10.8 Hz, 2H, H, one of CH_2_-18 (A), one of CH_2_-18 (B)), 4.96 (m, 2H, H-2' (A), H-2' (B)), 5.54 (dd, *J* = 17.4, 7.8 Hz, 2H, H-17 (A), H-17 (B)); ^**13**^**C NMR** (151 MHz, CDCl_3_/CD_3_OD 2:1 (v/v)), δ: 13.98 (**C**H_3_(CH_2_)_13_CH_2_C(O) (A), **C**H_3_(CH_2_)_13_CH_2_C(O) (B)), 18.98, 19.04, 19.11, 21.94, 22.04 ((C-16 (A), C-16 (B), C-22 (A), C-22 (B), C-20 (A), C-20 (B), C-21 (A), C-21 (B), C-19 (A), C-19 (B)), 21.03, 22.17, 22.38, 22.40, 23.97, 24.31, 24.32, 24.39, 28.65, 28.85, 28.87, 29.04, 29.17, 29.19, 29.21, 31.44, 36.80, 36.90, 36.96, 36.97, 37.18, 37.27, 38.91, 40.82. 40.85 (C-4 (A), C-4 (B), C-5 (A), C-5 (B), C-6 (A), C-6 (B), C-8 (A), C-8 (B), C-9 (A), C-9 (B), C-10 (A), C-10 (B), C-12 (A), C-12 (B), C-13 (A), C-13 (B), C-14 (A), C-14 (B), CH_3_(**C**H_2_)_12_CH_2_CH_2_C(O) (A), CH_3_(**C**H_2_)_12_CH_2_CH_2_C(O) (B), 27.49 (C-15 (A), C-15 (B)), 32.25, 32.27, 32.29, 32.31 (C-7 (A), C-7 (B), C-11 (A), C-11 (B)), 33.75 (CH_3_(CH_2_)_13_**C**H_2_C(O) (A), CH_3_(CH_2_)_13_**C**H_2_C(O) (B)), 38.76, 38.77 (C-3 (A), C-3 (B)), 44.60, 44.64 (C-2 (A), C-2 (B)), 53.58 (t, *J* = 3.5 Hz, -N(**C**H_3_)_3_ (A), -N(**C**H_3_)_3_ (B)), 58.90 (d, *J* = 4.9 Hz, C-α (A), C-α (B)), 61.89 (C-1' (A), C-1’ (B)), 63.52 (d, *J* = 5.4 Hz, C-3' (A), C-3' (B)), 65.87 (m, C-β (A), C-β (B)), 69.87 (d, *J* = 8.1 Hz, C-2'(A)), 69.89 (d, *J* = 8.0 Hz, C-2'(B)), 111.60 (C-18 (A), C-18 (B)), 145.00 (C-17 (A), C-17 (B)), 171.42 (C-1 (A), C-1 (B)), 173.10 (CH_3_(CH_2_)_13_CH_2_**C**(O) (A), CH_3_(CH_2_)_13_CH_2_**C**(O) (B)); ^**31**^**P NMR** (243 MHz, CDCl_3_/CD_3_OD 2:1 (v/v)), δ: -0.97; HRMS (ESI): *m/z* calcd. for C_46_H_90_NO_8_P [M + H]^+^ 816.6483; found 816.6492.

### Biological studies

The biological studies were performed *in vitro* using human cancer cell lines: MV4-11 (human biphenotypic B myelomonocytic leukaemia), A-549 (non-small cell lung cancer), MCF-7 (breast cancer), LoVo (colon cancer), LoVo/DX (colon cancer drug resistant), HepG2 (liver cancer) and BALB/3T3 (normal mice fibroblasts). These lines were obtained from American Type Culture Collection (Rockville, Maryland, U.S.A.). The cell line is being maintained in the Institute of Immunology and Experimental Therapy, Wroclaw, Poland. All cell lines were grown at 37°C with 5% CO_2_ humidified atmosphere and were cultured in cultured medium according to the method described before [25].

Prior to usage, the compounds were dissolved in DMSO or in mixture of 99.8% ethanol and DMSO (1:1) to the concentration of 25 or 50 mM, and subsequently diluted in culture medium to reach the required concentrations (ranging from 5 to 625 μM).

Twenty-four hours prior to the addition of tested compounds, the cells were plated in 96-well plates (Sarstedt, Germany) at a density of 1 × 10^4^ cells per well in 100 μL of culture medium. The assay was performed after 72 h of exposure to varying concentrations of the tested agents. The *in vitro* cytotoxic effect of tested agents was examined using the MTT (for MV4-11 cells) or SRB assay which were described previously [25]. Each compound in each concentration was tested in triplicate in a single experiment, which was repeated 3–5 times.

The results of antiproliferative activity were given as IC_50_ values. Using this parameter we calculated also the resistance indexes (RI) dividing the IC_50_ values of selected compounds estimated for drug resistant cell LOVO/DX line by respective values designated for drug sensitive LoVo line. According to Harker et al. [26] three categories of the cells could be distinguished: (a) the cells are drug-sensitive—if the ratio approaches 0–2; (b) the cells are moderately drug-resistant—if the ratio ranges from 2 to 10; (c) the cells are markedly drug-resistant—if the ratio is higher than 10.

#### Statistical analysis

Statistical analysis was performed using STATISTICA version 10 (StatSoft Inc., USA). Mann-Whitney U Test was used in the analysis and the results in Tables 1 and 2 are given with statistical significance in comparison to free isoprenoid acids p < 0.05.

**Table 1:** Antiproliferative activity of isoprenoid-phospholipid conjugates against human leukemia MV4-11 cell line.

Entry	Compounds	Acyl residue		Cell line
				IC50 [μM]
		*sn*-1	*sn*-2	MV4-11
**1**	**Palmitic acid**	-	-	161±78.9
**2**	**(DPPC)**	PA	PA	178.74±30.25
**3**	**(1-PA-LPC)**	PA	-	64±0.93
**4**	**1 (GERA)**	-	-	255.5±86.1
**5**	**6a**	GERA	GERA	50.1±3.5*
**6**	**7a**	PA	GERA	95±15.2*
**7**	**8a**	GERA	-	268.74±18
**8**	**9a**	GERA	PA	31.76±1.62*
**9**	**2 (FARA)**	-	-	152.57±20.05
**10**	**6b**	FARA	FARA	27.78 ± 7.52**
**11**	**7b**	PA	FARA	50.31±5.37**
**12**	**8b**	FARA	-	111.69±39.77
**13**	**9b**	FARA	PA	298.76±39.26
**14**	**3 (DAA)**	-	-	131.8±52.73
**15**	**6c**	DAA	DAA	38.65±11.08***
**16**	**7c**	PA	DAA	65.11±12.23
**17**	**8c**	DAA	-	202.84±15.04
**18**	**9c**	DAA	PA	49.97±9.0***
**19**	**4 (PHYTA)**	-	-	**39.28±5.85**
**20**	**6d**	PHYTA	PHYTA	42.38±9.52
**21**	**7d**	PA	PHYTA	259.13±32.5
**22**	**8d**	PHYTA	-	47.38±6.35
**23**	**9d**	PHYT	PA	258.63±36.97
**24**	**Cisplatin**			1.3±0.47

IC50 –compound concentration leading to 50% inhibition of cell proliferation. Data are presented as mean ± SD of 3–5 independent experiments.

*—results within column which are significantly different in comparison to GERA, p<0.05.

**—results within column which are significantly different in comparison to FARA, p<0.05.

***—results within column which are significantly different in comparison to DAA, p<0.05.

Statistical analysis was performed using STATISTICA version 10 (StatSoft Inc., USA). Mann-Whitney U Test was used in the analysis.

**Table 2:** Antiproliferative activity of terpene-phospholipids against human cancer cell lines.

Compounds	Acyl residue		Cell lines					
			IC_50_ [μM]					
	*sn*-1	*sn*-2	A-549	MCF-7	LOVO	LOVO/DX	HepG2	BALB/3T3
**Palmitic acid**	-	-	56.4±1.6	122.6±63.4	42.1±4.6	86.3±21.3	275.1±85.2	60.4±4
**DPPC**	PA	PA	229.7±31.3	398.2±193.9	274.2±17.1	n.a.	n.a.	48.8±2.1
**1-PA-LPC**	PA	-	62.3±6.5	73±4.6	52.4±4.3	58±3.7	282.7±12.5	176.2±11.3
**1 (GERA)**	-	-	301±66.9	310.4±84	329.3±54.5	281.4±13.6	517.4±98.3	n.a.
**6a**	GERA	GERA	262.8±19.7	208.6±12.9	230.4±6.9 *	245.4±19.7 *	286.4±1.3 *	279.8±8.2
**7a**	PA	GERA	72.6±1.6*	66.6±4.2*	57.4±0.7*	154.3±7.9*	159.1±31.2*	246.2±13.9
**8a**	GERA	-	n.a.	372±28.1	245.1±68.5	469±3.2	n.a.	n.a.
**9a**	GERA	PA	38.3±6.7*	40±1.9*	32.8±5.1*	52.7±3.3*	59.2±4.6*	57.6±1
**2 (FARA)**	-	-	91±11.5	161.6±5.9	99.17±26	87±14.4	266.9±15.5	229.5±40.3
**6b**	FARA	FARA	30.4±1.5**	45±1.4**	13.6±0.7**	57.6±0.6**	53.6±5.1**	57.8±1.4**
**7b**	PA	FARA	110.9±12.9	98.7±15.6**	251.4±22.7**	486.4±17.4**	271.6±24.7	518±69.8**
**8b**	FARA	-	260.4±33.6**	249.6±45.2**	241.2±15.1**	280±44.4**	344.3±40.9**	296.9±18.6**
**9b**	FARA	PA	n.a.	489.4±57.7**	269.4±29.86	537.4±61.2**	n.a.	n.a.
**3 (DAA)**	-	-	149.5±16.8	230.8±43.2	172.6±38.7	109.2±16	273.6±12	296.6±14.4
**6c**	DAA	DAA	52.3±1.9***	57.8±2.6***	53.1±3.6***	56.9±0.2***	56.7±0.1***	57.3±0.3***
**7c**	PA	DAA	75.7±10.6***	89.7±11.3***	54.4±3.2***	111±19.4	134.5±51***	275.8±25.7
**8c**	DAA	-	n.a.	469.6±52.9***	283.5±9.7***	382.1±32.4***	n.a.	n.a.
**9c**	DAA	PA	68.3±1.5***	102.3±11.2***	61.5±4.84***	75.5±8.1***	303.2±77.6	291.9±41.5
**Cisplatin**			8.6 ± 0.7	8.1 ± 0.03	2.56±0.35	3.17±0.2	2.38±0.64	4.2 ± 1.1
**Doxorubicin**			-	-	0.117±0.012	6.53±0.93	-	-

n.a.—no activity in concentration of 5, 25, 125, 625 μM. IC50 –compound concentration leading to 50% inhibition of cell proliferation. Data are presented as mean ± SD of 3–5 independent experiments.

*—results within column which are significantly different in comparison to GERA, p<0.05.

**- results within column which are significantly different in comparison to FARA, p<0.05.

***—results within column which are significantly different in comparison to DAA, p<0.05.

Statistical analysis was performed using STATISTICA version 10 (StatSoft Inc., USA). Mann-Whitney U Test was used in the analysis.

## Results and discussion

Fundamental obstacle in pharmaceutical applications of therapeutic compounds are often their low bioavailability, incomplete absorption or too rapid absorption and too fast excretion as well as difficulties with preparing its appropriate formulations. Good strategy to solve these types of problems is designing the lipidic prodrugs. In this way the biologically active molecules can be covalently bound to the transport moiety and then release *in vivo* providing the effective treatment after oral administration. The transport molecules can be fatty acids, triglycerides or phospholipids. The simplest method of lipophilization is the esterification of carboxylate group of fatty acids by drug molecules or their attachment to the ω-position of the fatty chain. In the case of triglycerides one or more fatty acids joined to the skeleton of glycerol can be replaced by other molecules. These kinds of drug-lipid conjugates were prepared for example for non-steroidal anti-inflammatory compounds aspirin [27], indomethacin [28] or naproxen [29] and many other drugs including _L_-dopa [30], phenytoin [31] and GABA [32]. In drug delivery system (DDS) phospholipids play the most important role among lipids. Because of their amphiphilic structure in the last decade phospholipid-related formulations like Doxil^®^, Cleviprex^®^, Valium^®^ or Silybin Phytosome have been used in clinical trials [21]. Phospholipids as drug carriers have been applied for cytostatic nucleosides and nucleoside analogues, acyclovir and cytidine analogues [33]. The replacement of polar head of PC by steroids [34], phenylalkanols [35,36] and isoprenoid alcohols [37,38] have been also described in the literature. Yamamoto and coworkers observed the increase of biological activity of phosphatidylated-monoterpene alcohols [38]. It convinced us to explore the field of synthesis of new class of isoprenoid-phospholipid conjugates in order to extend application of isoprenoids as the health promoting molecules. Recently we synthesized novel phosphatidylcholines containing isoprenoid compounds in the *sn*-1 and *sn*-2 positions of PC [22]. This strategy based on the introducing a drug molecule instead of fatty acids has been investigated so far by only a few research groups [33,39,40].

In our previous paper we reported on the higher activities of geranic and citronellic acids introduced in apolar part of PC towards selected cancer cell lines [22]. For better understanding the effect of conjugation of phospholipids with isoprenoid acids on the antiproliferative activity in living cells we designed and prepared more terpene phospholipids derivatives.

**Fig 1:**
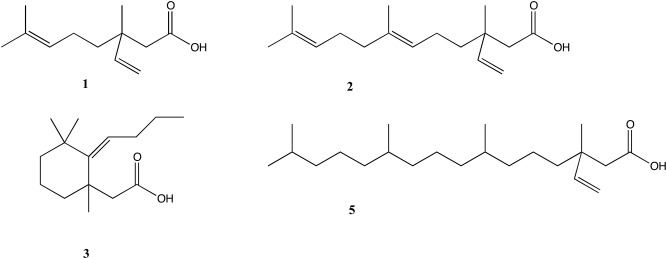
Prepared acids used in the synthesis of terpene phospholipids.

As a substrate of phospholipid modifications optically pure GPC with naturally *R*-configuration of chiral center was used. The synthetic sequence has started from preparing four synthetic isoprenoid acids: 3,7-dimethyl-3-vinyloct-6-enoic acid (**1**), (*E*)-3,7,11-trimethyl-3-vinyldodeca-6,10-dienoic acid (**2**), 2-(2-butylidene-1,3,3-trimethylcyclohexyl)acetic acid (**3**) and 3,7,11,15-tetramethyl-3-vinylhexadecanoic acid (**5**). Below we present (Fig 1) the structures of synthetized acids (**1–3, 5**).

**Fig 2:**
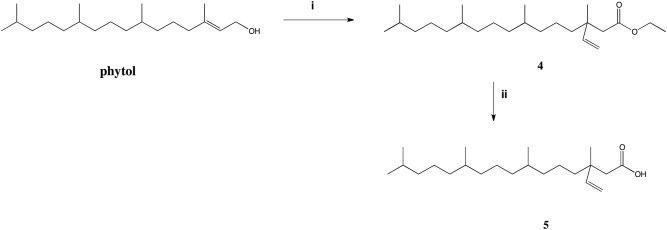
Synthesis of acid (5). Reagents (i) CH_3_C(OEt)_3_, CH_3_COOH, 138°C; (ii) KOH, EtOH.

Acids were obtained according to known two-step synthesis from the corresponding alcohols: geraniol, farnesol, β-dihydrodamascol and phytol. Synthesis of acids **2** and **3** from farnesol and β-dihydrodamascol was described in the literature [23, 24], the same procedure was applied for the synthesis of known [41] acid **1** from geraniol while the structure of ester (**4**) and acid (**5**) obtained from natural compound phytol is first time reported. The main step of synthesis of isoprenoid acids was the Johnson-Claisen rearrangement of allyl alcohols. Then formed esters were hydrolyzed in ethanolic solution of KOH to give corresponding acids (Fig 2).

**Fig 3:**
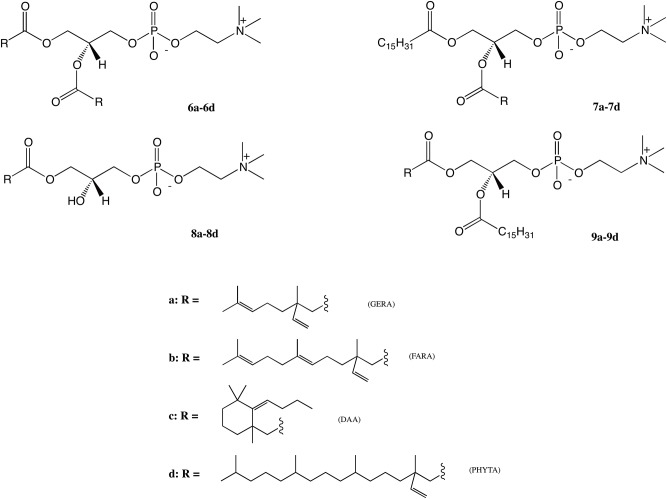
Structures of synthesized isoprenoid phospholipids.

Four groups of terpene-modified phosphatidylcholines were synthesized (Fig 3). We have started from the synthesis of symmetrical phosphatidylcholines with the same acyl residues in both *sn*-1 and *sn*-2 positions (**6a-6d**). For this purpose we used the cadmium salt of *sn*-glycero-3-phosphocholine (GPC×CdCl_2_) as a starting material and series of isoprenoid acids previously prepared previously, following the procedure published before [22]. Novel 1,2-diisoprenoyl-*sn*-glycero-3-phosphocholines (**6a-6d**) were obtained in good 62%-97% yields after 72 hours of reaction. The asymmetrically substituted terpene-phospholipids (**7a-7d**) were obtained by the esterification of the hydroxy group of 1-palmitoyl-2-hydroxy-*sn*-glycero-3-phosphocholine with isoprenoid acids (**1–3, 5**). A detailed description of this reaction conditions has been presented previously [22]. The 1-pamitoyl-2-isoprenoyl-*sn*-glycero-3-phosphocholines (**7a-7d**) has not been reported in the literature and was obtained in 41%-58% yields. Lysophosphatidylcholines with isoprenoid acids in the *sn*-1 position were synthesized from GPC, which was first transformed into cyclic stannylene ketal by treatment with DBTO and then selectively acylated with isoprenoyl chlorides. According to this methodology four new 1-isoprenoyl-2-hydroxy-*sn*-glycero-3-phosphocholines (**8a-8d**) in 24%-52% yields were obtained. The last synthetic path involved the key intermediates **8a-8d** in acylation of free hydroxy groups in the *sn*-2 position with palmitic acid (PA) in the presence of DCC and DMAP. The final products **9a-9d** were obtained in 47–68% yields. The structures of the final products were confirmed by their spectroscopic data and the detailed assignments of signals are given in the supplementary material.

All synthesized compounds **6a-6d**, **7a-7d**, **8a-8d** and **9a-9d** were evaluated for their antiproliferative activity. The preliminary biological studies were performed towards human leukemia cell line (MV4-11). The results are summarized in Table 1 and are expressed as the IC_50_-concentration of the compounds (in μM) that inhibits proliferation of the cells by 50% compared to the untreated control cells. The clinical chemotherapy drug cisplatin was used in this evaluation as a positive control. In order to observe the expected increase of activity of isoprenoids after their conjugation with phospholipid molecule free terpene acids were also tested. On the other hand trying to estimate the benefits resulting from the connection of two valuable groups of compounds the cytotoxic effect of palmitic acid and phosphatidylcholine with this fatty acid in both *sn*-1 and *sn*-2 positions as well as lysophosphatidylcholine with palmitic acid only in *sn*-1 position were also evaluated.

Analyzing the results presented in Table 1 significant increase of antiproliferative activity of monoterpene (**1**) and sesquiterpene (**2**, **3**) acids derivatives attached to phospholipid moiety can be observed. The activity of isoprenoid phospholipids **6a-6d**, **7a-7d**, **8a-8d** and **9a-9d** is generally from 3 to 8-fold higer towards MV4-11 compared to free terpene acids. These results suggest that the phospholipid moiety increases inhibition of the proliferation of cancer cells by isoprenoid acids. That anticancer effect is also related to the presence and position of isoprene moiety in the phospholipid molecule. The best results for sesquiterpene acyl derivatives are observed for diisoprenoyl-PC (entries 10, 15) whereas for derivatives of monoterpene acid 1-isoprenoyl-2-palmitoyl-PC (entry 5) exhibits the highest activity. The highest activity towards leukemia cells was achieved for derivative **6b** when 2,3-dihydro-3-vinylfarnesoic acids was directly connected to glycerol skeleton of PC in both *sn*-1 and *sn*-2 positions (IC_50_ = 27.78 μM). Diterpene phytol derivative **5** does not exhibit higher cytotoxic effect on leukemia cells after introduction to *sn*-glycero-3-phosphocholine and from that reason it was not selected to further studies.

In the second step of evaluation studies we tested the selectivity of phospholipid derivatives of mono- and sesquiterpene acids **6a-6c**, **7a-7c**, **8a-8c** and **9a-9c** towards human cancer cell lines with different origin. The experiments were also carried out towards normal mice fibroblasts BALB/3T3 and synthesized compounds were evaluated at four concentration levels 5, 25, 125 and 625 μM. As it is shown in the Table 2 most of investigated terpene acids are more active after their introduction into GPC. Consequently, diisoprenoyl-PC of sesquiterpene acid derivatives (**2**, **3**) and monoisoprenoyl-PC of monoterpene acid derivatives (**1**) turned out to be the most active compounds towards tested cancer cell lines. The compounds **6b** and **6c** that contained 2,3-dihydro-3-vinylfarnesoic and 2-(2-butylidene-1,3,3-trimethylcyclohexyl)acetic acids in both *sn*-1 and *sn*-2 positions of PC were more active than the corresponding derivatives substituted with other acyl donors. Compound **6b** showed the highest cytotoxic effect against lung (A549), colon (LoVo) and liver (HepG2) cancer cell lines among all tested derivatives (IC_50_ = 13.6–57.8 μM). It is worth noting that **6b** derivative the highest activity against colon cancer cell line (IC_50_ = 13.6 μM) is only 5-fold lower than those exhibited by cisplatin. Apart from that compound **6b** had a lower cytotoxicity against the normal mice fibroblasts cell BALB/3T3 (57.8 μM) than cisplatin. The cells of breast cancer (MCF-7) and drug resistant colon cancer (LoVo/DX) were the most sensitive on the phosphatidylcholine **9a** contained 2,3-dihydro-3-vinylgeranic acid in *sn*-1 position (IC_50_ = 40.0 and 52.7 μM respectively). No significant differences in antiproliferative activity against normal and cancer cell lines was found for tested derivatives however for the most active compounds **9a** and **6b** the doses active towards leukemia, lung, breast and colon cancer cell lines are lower in comparison to cytotoxic doses active against BALB/3T3.

IC_50_ values for newly synthesized terpene phospholipids were compared with the results previously reported for phospholipid derivatives of geranic and citronellic acids [22]. Based on these data we tried to determine the structure-activity relationship for prepared compounds. Particularly it can be noticed that the antitumor activity depends on the length of the isoprenoid moiety attached to PC. Whereas the mono- and sesquiterpene derivatives are active towards selected cancer cell lines, incorporation diterpene phytol derivative into PC does not increase its biological activity. In addition, it can be observed that linear terpene acids joined to PC are more active than the cyclic one. The number of unsaturated bounds in the structure of isoprenoid acyl donors also affects the activity of studied products. The derivatives of geranic and 2,3-dihydro-3-vinylgeranic acids are more active than derivatives of citronellic acid. However the differences in the antiproliferative activity between isoprenoids with three and four double bounds seem to have selective effect depending on the type of cancer cell line.

Additionally we calculated also the resisitance indexes (RI) for all tested compounds and we found that almost all of them are able to overcome the barrier of P-gp-dependent resistance with exception of three derivatives **7a**, **6b** and **7c** (Table 3).

**Table 3:** Resistance index (RI) values of terpene-phospholipids.

Compounds	Acyl residue		
	*sn*-1	*sn*-2	RI
**1 (GERA)**	-	-	0.85
**5a**	GERA	GERA	1.07
**6a**	PA	GERA	2.69
**7a**	GERA	-	1.91
**8a**	GERA	PA	1.61
**2 (FARA)**	-	-	0.88
**5b**	FARA	FARA	4.23
**6b**	PA	FARA	1.93
**7b**	FARA	-	1.16
**8b**	FARA	PA	1.99
**3 (DAA)**	-	-	0.63
**5c**	DAA	DAA	1.07
**6c**	PA	DAA	2.04
**7c**	DAA	-	1.35
**8c**	DAA	PA	1.23
**DOX**	-	-	55.81

DOX—doxorubicin

**RI** was calculated according to the formula RI = (IC_50_ estimated against resistant cell line)/(IC_50_ estimated against non-resistant cell line); values range: 0<RI<2-indicate that the tested compound is able to overcome drug resistance; 2<RI<10 –defines the moderate ability of the compound to overcome drug resistance; RI>10 –defines no influence on the drug resistance phenomenon.

In summary, a series of sixteen new isoprenoid phospholipid conjugates containing isoprenoid acids were synthesized and their antiproliferative activity towards selected cancer cell lines was investigated. Our results clearly showed that incorporation isoprenoids acids with phosphatidylcholine significantly improves its selective cytotoxic activity. Short chain isoprenoid acids like geranic, citrolellic or 3,7-dimethyl-3-vinyloct-6-enoic are more active in the form of monoisoprenoilo-PC whereas 2,3-dihydro-3-vinylfarnesoic and 2-(2-butylidene-1,3,3-trimethylcyclohexyl)acetic acids are active after their incorporation in both *sn*-1 and *sn*-2 position of PC. However, studying the structure-activity relationship for isoprenoid-phospholipids it is nessecery to prepare more derivatives. At this stage promising results of *in vitro* activity qualify **6b**, **6c** and **9a** as anticancer agents for further *in vivo* studies and enhance investigation of their mechanism of action.

## Supporting information

S1 Fig ^1^H NMR spectrum of 6a. (DOCX)

S2 Fig ^13^C NMR spectrum of 6a. (DOCX)

S3 Fig ^31^P NMR spectrum of 6a. (DOCX)

S4 Fig ^1^H—^1^H COSY spectrum of 6a. (DOCX)

S5 Fig HSQC spectrum of 6a. (DOCX)

S6 Fig ^1^H NMR spectrum of 6b. (DOCX)

S7 Fig ^13^C NMR spectrum of 6b. (DOCX)

S8 Fig ^31^P NMR spectrum of 6b. (DOCX)

S9 Fig ^1^H—^1^H COSY spectrum of 6b. (DOCX)

S10 Fig HSQC spectrum of 6b. (DOCX)

S11 Fig ^1^H NMR spectrum of 6c. (DOCX)

S12 Fig ^13^C NMR spectrum of 6c. (DOCX)

S13 Fig ^31^P NMR spectrum of 6c. (DOCX)

S14 Fig ^1^H—^1^H COSY spectrum of 6c. (DOCX)

S15 Fig HSQC spectrum of 6c. (DOCX)

S16 Fig ^1^H NMR spectrum of 6d. (DOCX)

S17 Fig ^13^C NMR spectrum of 6d. (DOCX)

S18 Fig ^31^P NMR spectrum of 6d. (DOCX)

S19 Fig ^1^H—^1^H COSY spectrum of 6d. (DOCX)

S20 Fig HSQC spectrum of 6d. (DOCX)

S21 Fig ^1^H NMR spectrum of 7a. (DOCX)

S22 Fig ^13^C NMR spectrum of 7a. (DOCX)

S23 Fig ^31^P NMR spectrum of 7a. (DOCX)

S24 Fig ^1^H—^1^H COSY spectrum of 7a. (DOCX)

S25 Fig HSQC spectrum of 7a. (DOCX)

S26 Fig ^1^H NMR spectrum of 7b. (DOCX)

S27 Fig ^13^C NMR spectrum of 7b. (DOCX)

S28 Fig ^31^P NMR spectrum of 7b. (DOCX)

S29 Fig ^1^H—^1^H COSY spectrum of 7b. (DOCX)

S30 Fig HSQC spectrum of 7b. (DOCX)

S31 Fig ^1^H NMR spectrum of 7c. (DOCX)

S32 Fig ^13^C NMR spectrum of 7c. (DOCX)

S33 Fig ^31^P NMR spectrum of 7c. (DOCX)

S34 Fig ^1^H—^1^H COSY spectrum of 7c. (DOCX)

S35 Fig HSQC spectrum of 7c. (DOCX)

S36 Fig ^1^H NMR spectrum of 7d. (DOCX)

S37 Fig ^13^C NMR spectrum of 7d. (DOCX)

S38 Fig ^31^P NMR spectrum of 7d. (DOCX)

S39 Fig ^1^H—^1^H COSY spectrum of 7d. (DOCX)

S40 Fig HSQC spectrum of 7d. (DOCX)

S41 Fig ^1^H NMR spectrum of 8a. (DOCX)

S42 Fig ^13^C NMR spectrum of 8a. (DOCX)

S43 Fig ^31^P NMR spectrum of 8a. (DOCX)

S44 Fig ^1^H—^1^H COSY spectrum of 8a. (DOCX)

S45 Fig HSQC spectrum of 8a. (DOCX)

S46 Fig ^1^H NMR spectrum of 8b. (DOCX)

S47 Fig ^13^C NMR spectrum of 8b. (DOCX)

S48 Fig ^31^P NMR spectrum of 8b. (DOCX)

S49 Fig ^1^H—^1^H COSY spectrum of 8b. (DOCX)

S50 Fig HSQC spectrum of 8b. (DOCX)

S51 Fig ^1^H NMR spectrum of 8c. (DOCX)

S52 Fig ^13^C NMR spectrum of 8c. (DOCX)

S53 Fig ^31^P NMR spectrum of 8c. (DOCX)

S54 Fig ^1^H—^1^H COSY spectrum of 8c. (DOCX)

S55 Fig HSQC spectrum of 8c. (DOCX)

S56 Fig ^1^H NMR spectrum of 8d. (DOCX)

S57 Fig ^13^C NMR spectrum of 8d. (DOCX)

S58 Fig ^31^P NMR spectrum of 8d. (DOCX)

S59 Fig ^1^H—^1^H COSY spectrum of 8d. (DOCX)

S60 Fig HSQC spectrum of 8d. (DOCX)

S61 Fig ^1^H NMR spectrum of 9a. (DOCX)

S62 Fig ^13^C NMR spectrum of 9a. (DOCX)

S63 Fig ^31^P NMR spectrum of 9a. (DOCX)

S64 Fig ^1^H—^1^H COSY spectrum of 9a. (DOCX)

S65 Fig HSQC spectrum of 9a. (DOCX)

S66 Fig ^1^H NMR spectrum of 9b. (DOCX)

S67 Fig ^13^C NMR spectrum of 9b. (DOCX)

S68 Fig ^31^P NMR spectrum of 9b. (DOCX)

S69 Fig ^1^H—^1^H COSY spectrum of 9b. (DOCX)

S70 Fig HSQC spectrum of 9b. (DOCX)

S71 Fig ^1^H NMR spectrum of 9c. (DOCX)

S72 Fig ^13^C NMR spectrum of 9c. (DOCX)

S73 Fig ^31^P NMR spectrum of 9c. (DOCX)

S74 Fig ^1^H—^1^H COSY spectrum of 9c. (DOCX)

S75 Fig HSQC spectrum of 9c. (DOCX)

S76 Fig ^1^H NMR spectrum of 9d. (DOCX)

S77 Fig ^13^C NMR spectrum of 9d. (DOCX)

S78 Fig ^31^P NMR spectrum of 9d. (DOCX)

S79 Fig ^1^H—^1^H COSY spectrum of 9d. (DOCX)

S80 Fig HSQC spectrum of 9d. (DOCX)
