# Supplementary figures and images for: Isoprenoid-phospholipid conjugates as potential therapeutic agents: Synthesis, characterization and antiproliferative studies
Source: PLoS One. 2017 Feb 14;12(2):e0172238. doi: 10.1371/journal.pone.0172238 (PMC5308787; doi:10.1371/journal.pone.0172238)

## S1 Fig. 1H NMR spectrum of 6a

##
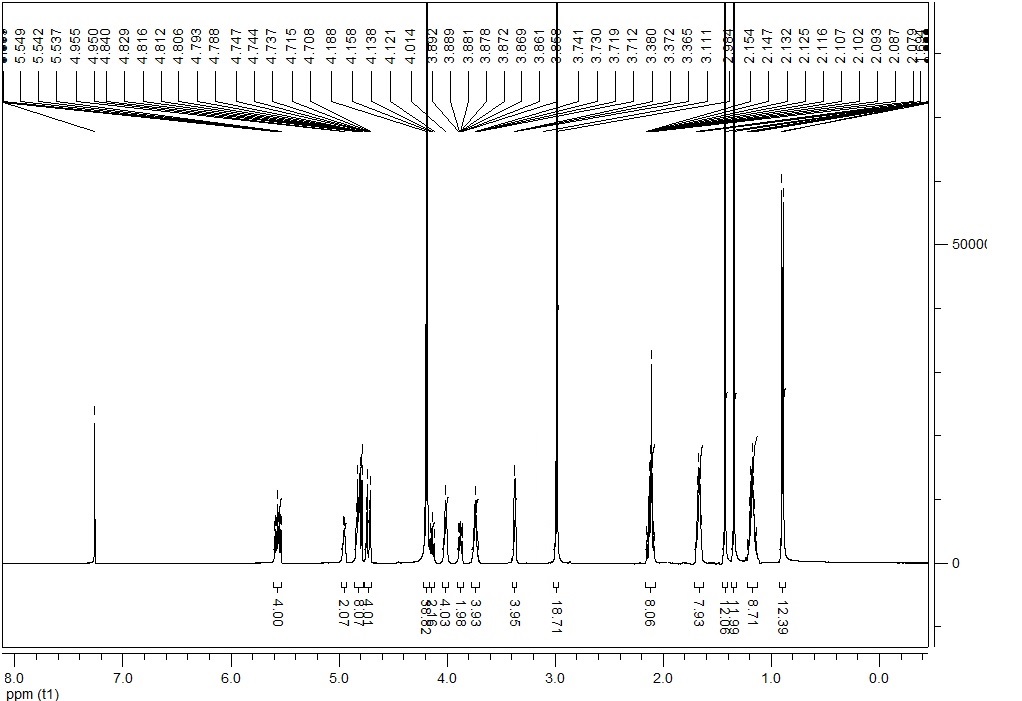

Supplement: S1 Fig — (DOCX) [file pone.0172238.s001.docx]

## S2 Fig. 13C NMR spectrum of 6a


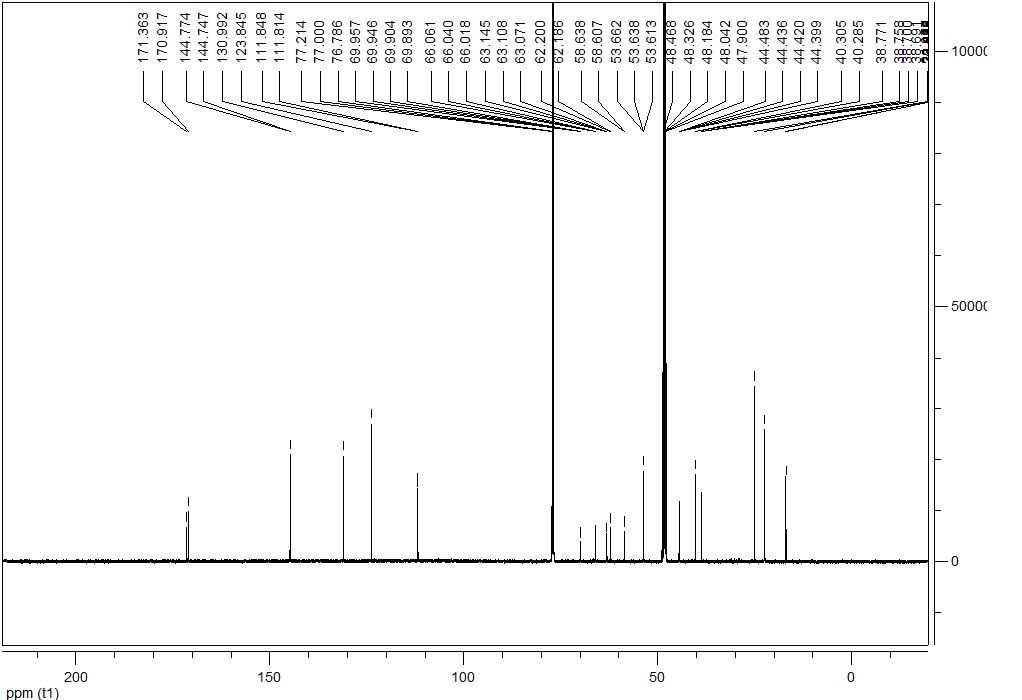

Supplement: S2 Fig — (DOCX) [file pone.0172238.s002.docx]

## S3 Fig. 31P NMR spectrum of 6a

**
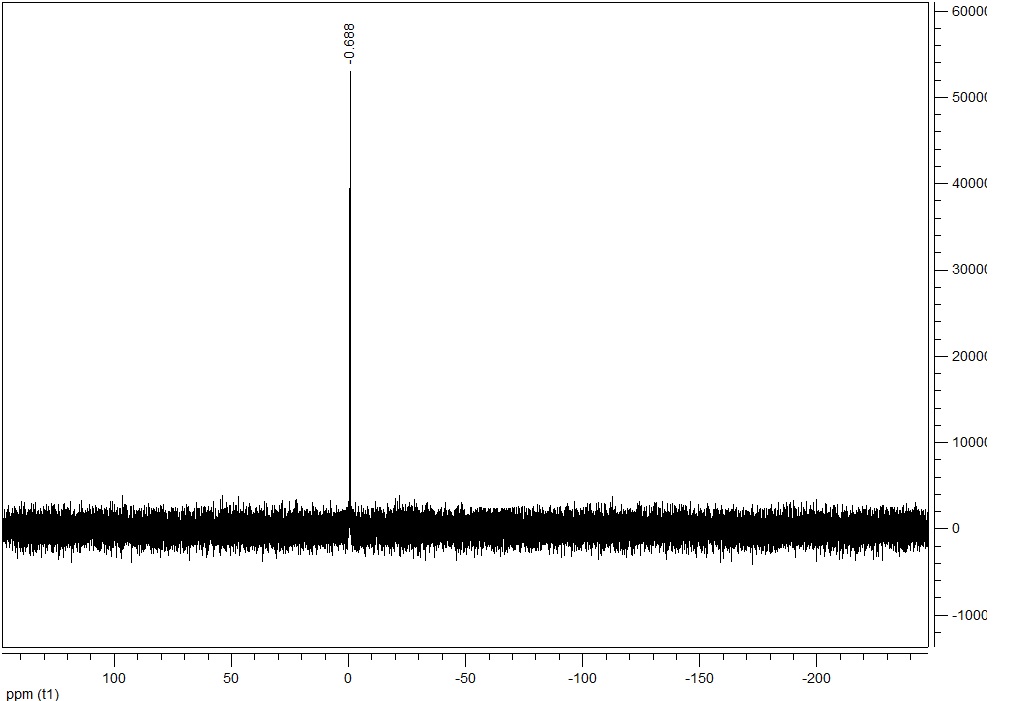
**

Supplement: S3 Fig — (DOCX) [file pone.0172238.s003.docx]

## S4 Fig. 1H – 1H COSY spectrum of 6a

**
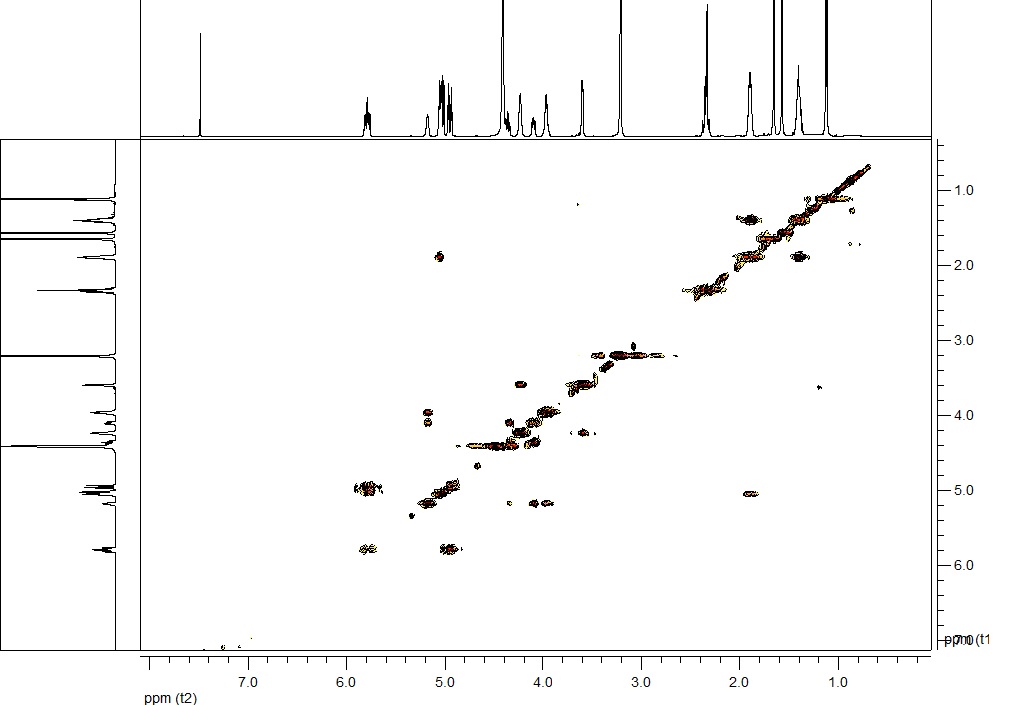
**

Supplement: S4 Fig — (DOCX) [file pone.0172238.s004.docx]

## S5 Fig. HSQC spectrum of 6a
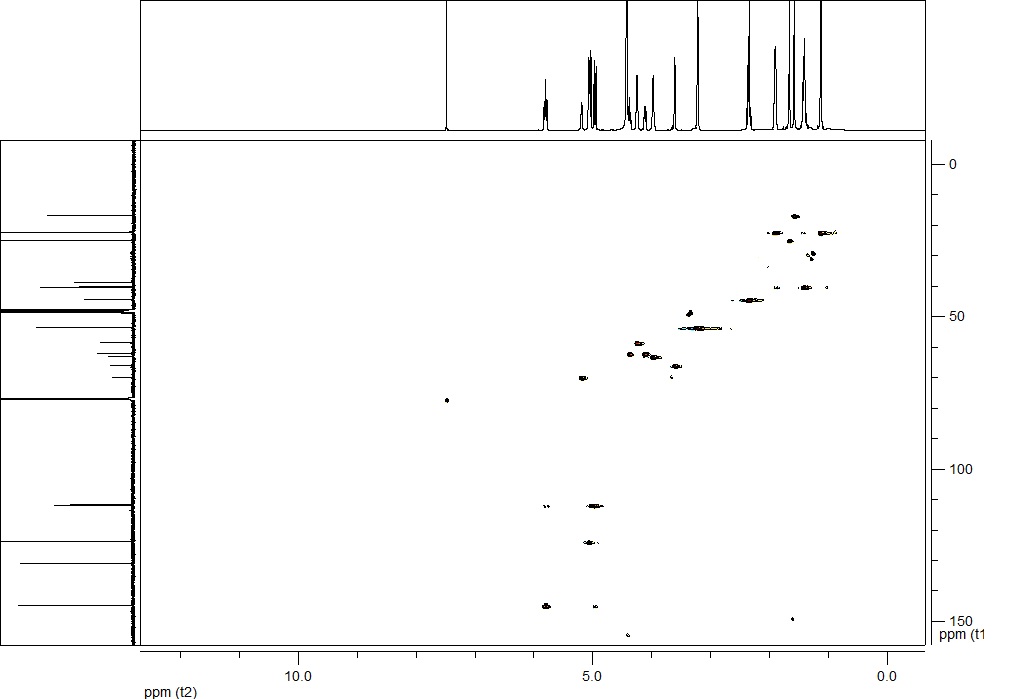

Supplement: S5 Fig — (DOCX) [file pone.0172238.s005.docx]

## S6 Fig. 1H NMR spectrum of 6b


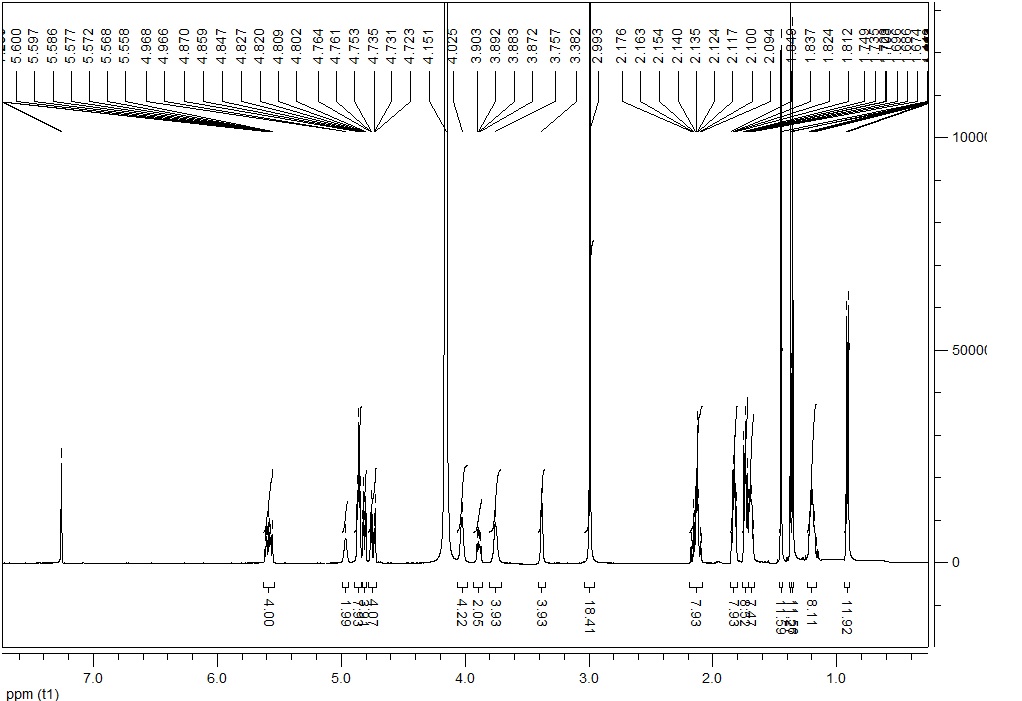

Supplement: S6 Fig — (DOCX) [file pone.0172238.s006.docx]

## S7 Fig. 13C NMR spectrum of 6b


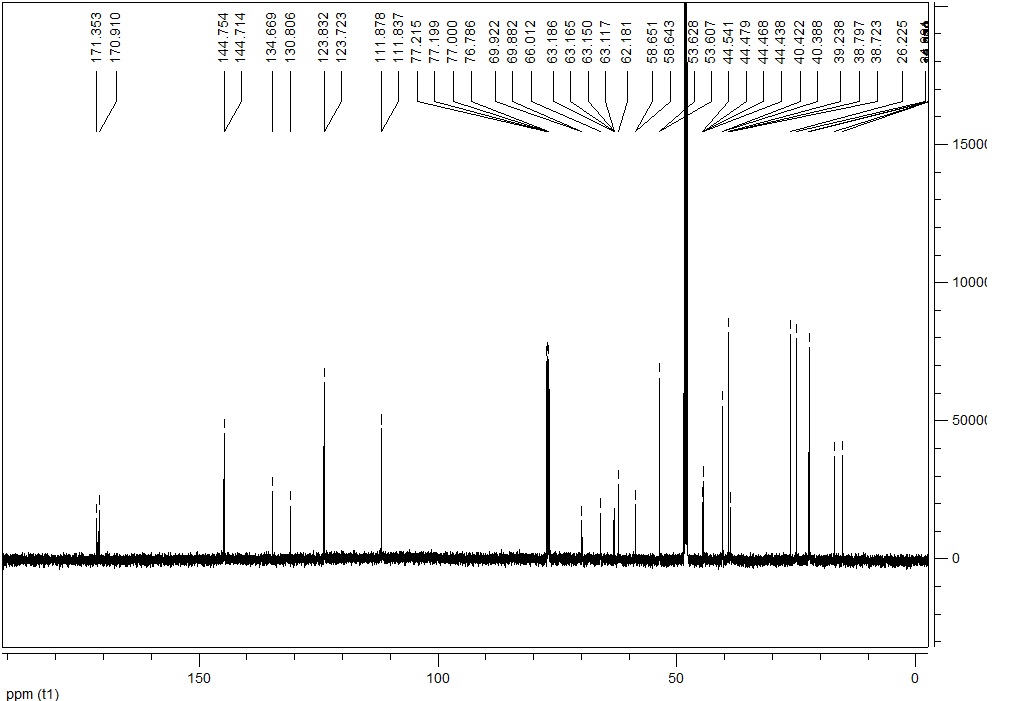

Supplement: S7 Fig — (DOCX) [file pone.0172238.s007.docx]

## S8 Fig. 31P NMR spectrum of 6b

**
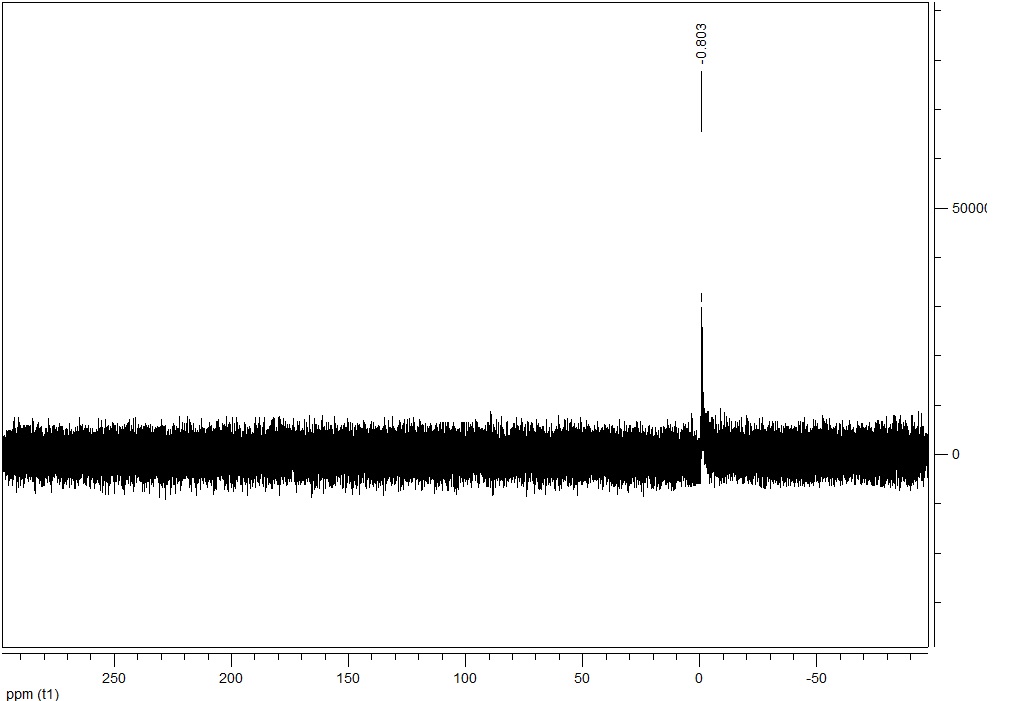
**

Supplement: S8 Fig — (DOCX) [file pone.0172238.s008.docx]

## S9 Fig. 1H – 1H COSY spectrum of 6b

**
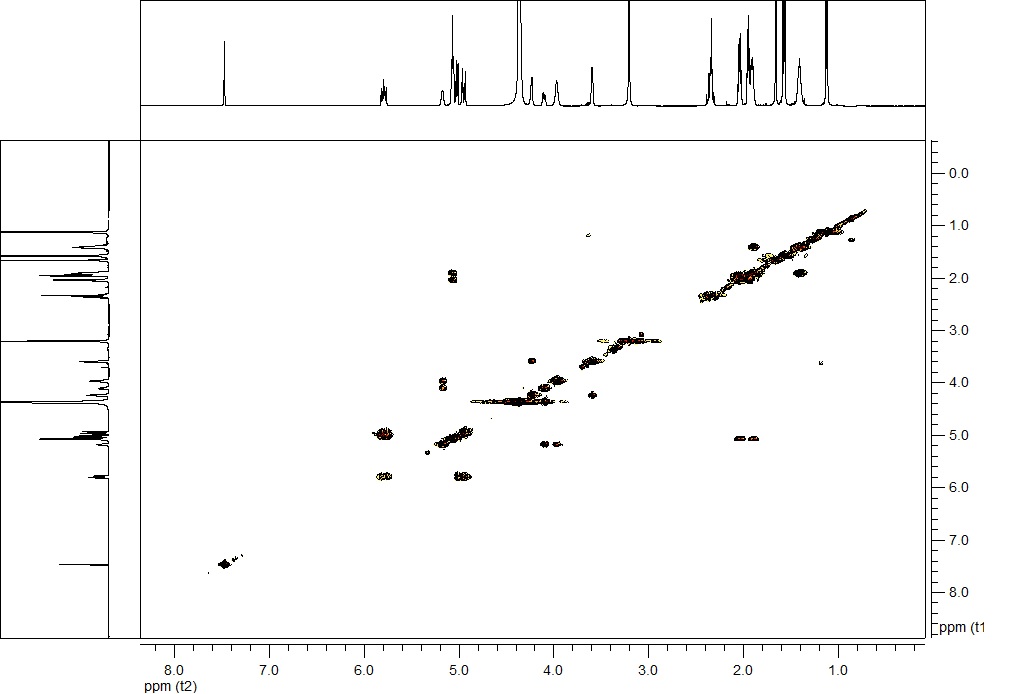
**

Supplement: S9 Fig — (DOCX) [file pone.0172238.s009.docx]

## S10 Fig. HSQC spectrum of 6b

**
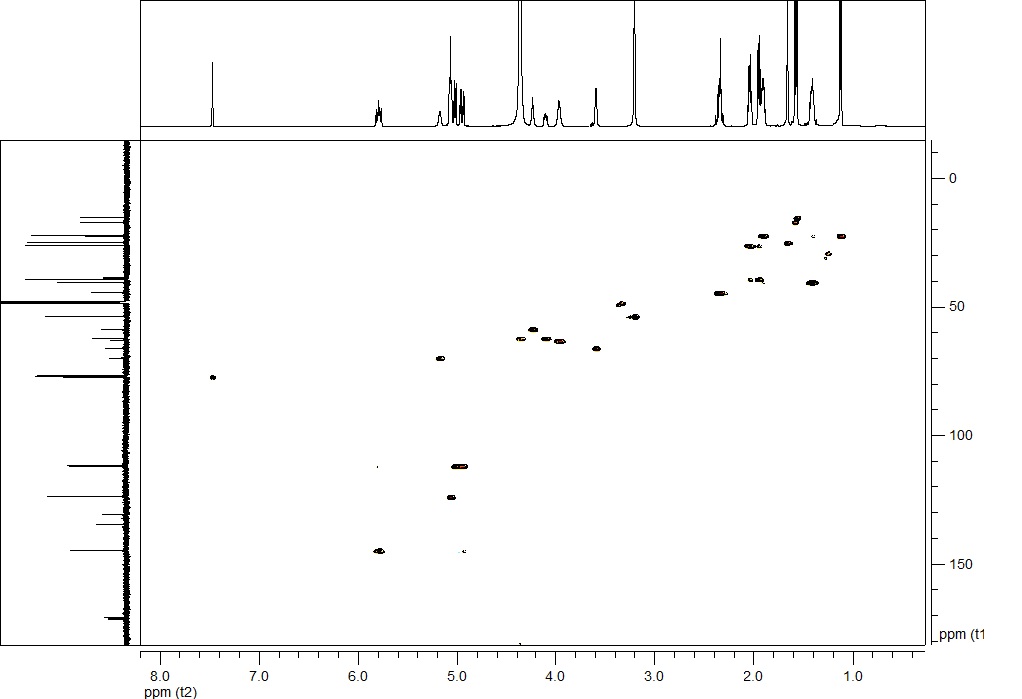
**

Supplement: S10 Fig — (DOCX) [file pone.0172238.s010.docx]

## S11 Fig. 1H NMR spectrum of 6c


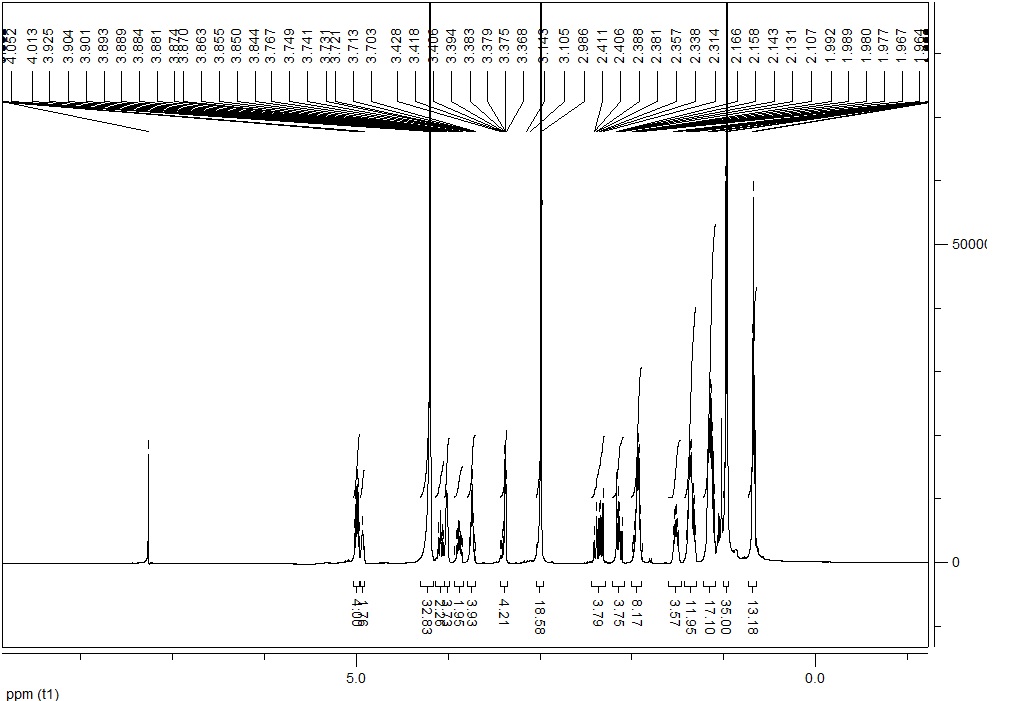

Supplement: S11 Fig — (DOCX) [file pone.0172238.s011.docx]

## S12 Fig. 13C NMR spectrum of 6c


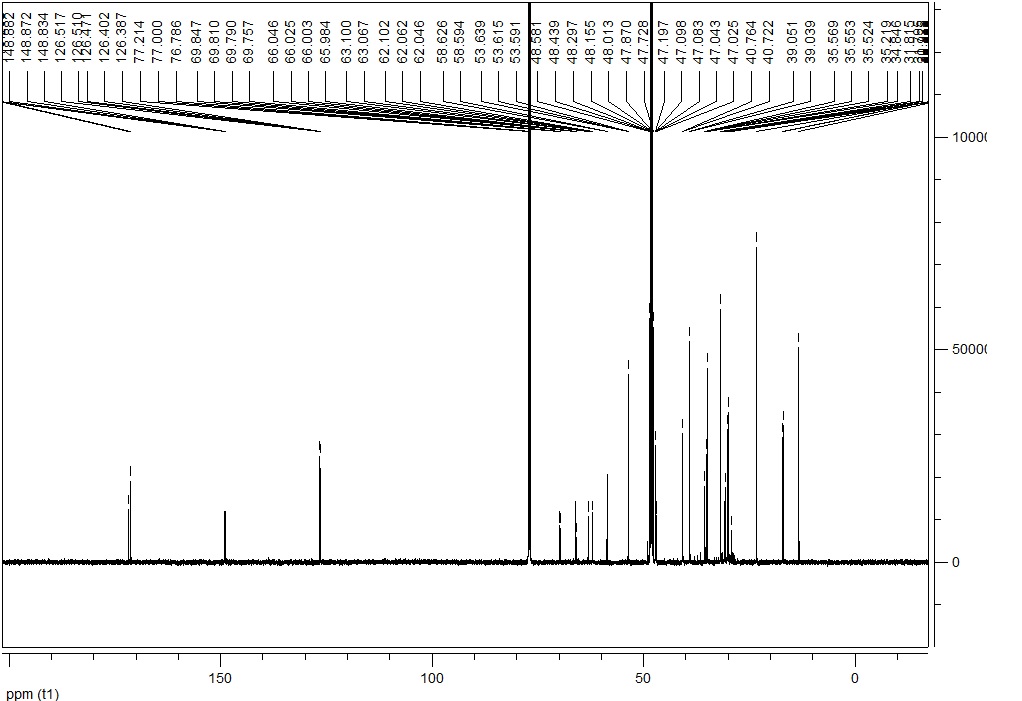

Supplement: S12 Fig — (DOCX) [file pone.0172238.s012.docx]

## S13 Fig. 31PNMR spectrum of 6c

**
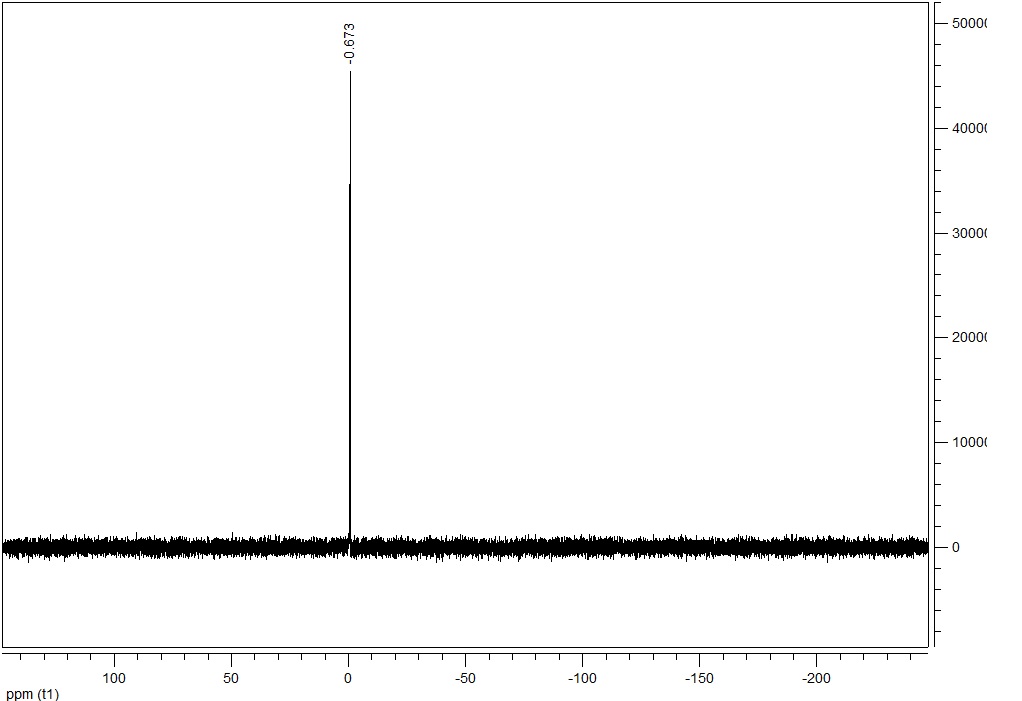
**

Supplement: S13 Fig — (DOCX) [file pone.0172238.s013.docx]

## S14 Fig. 1H – 1H COSY spectrum of 6c

##
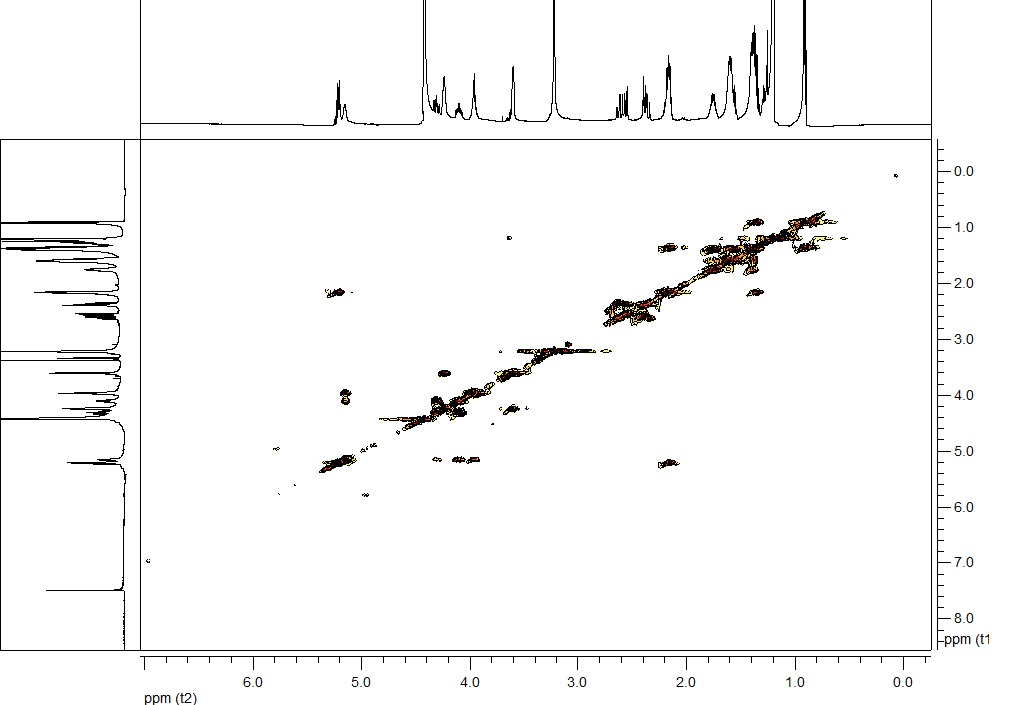

Supplement: S14 Fig — (DOCX) [file pone.0172238.s014.docx]

## S15 Fig. HSQC spectrum of 6c

**
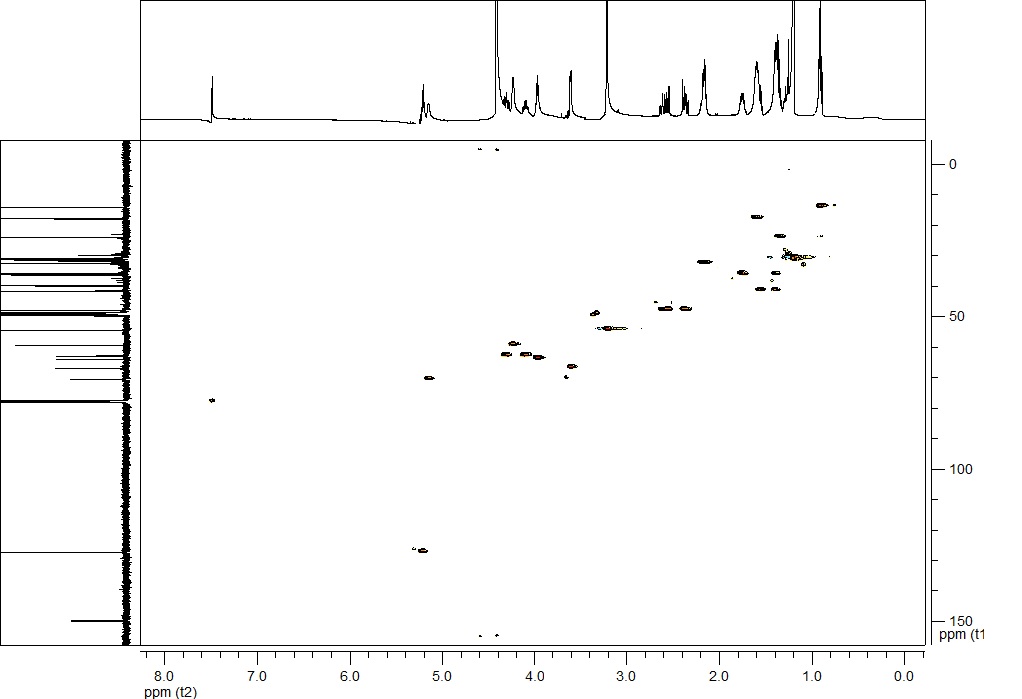
**

Supplement: S15 Fig — (DOCX) [file pone.0172238.s015.docx]

## S16 Fig. 1H NMR spectrum of 6d


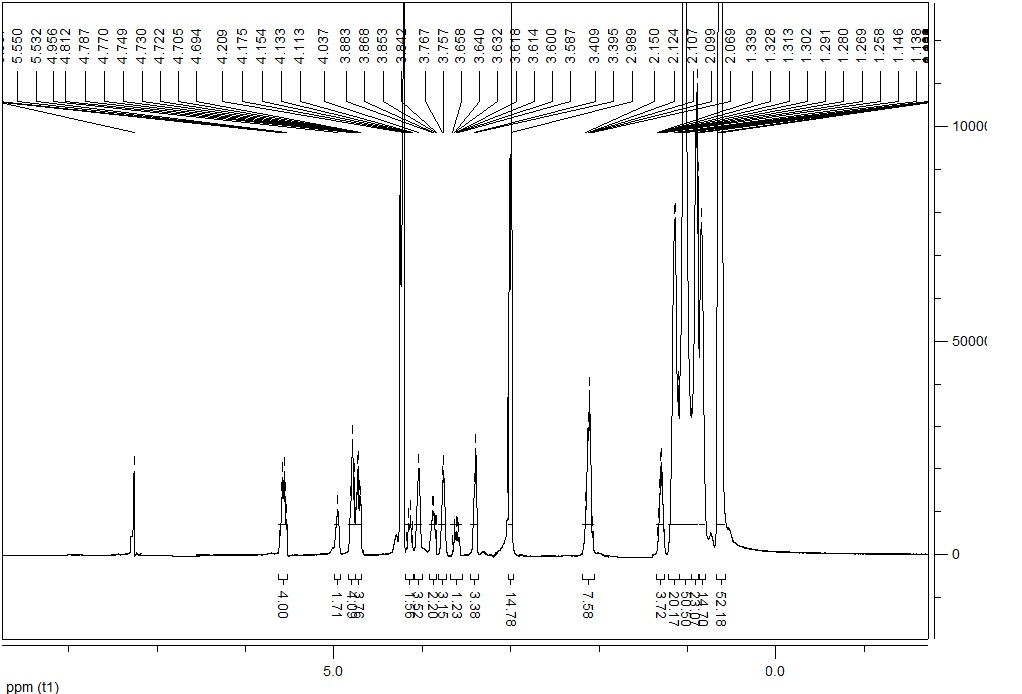

Supplement: S16 Fig — (DOCX) [file pone.0172238.s016.docx]

## S17 Fig. 13C NMR spectrum of 6d

##
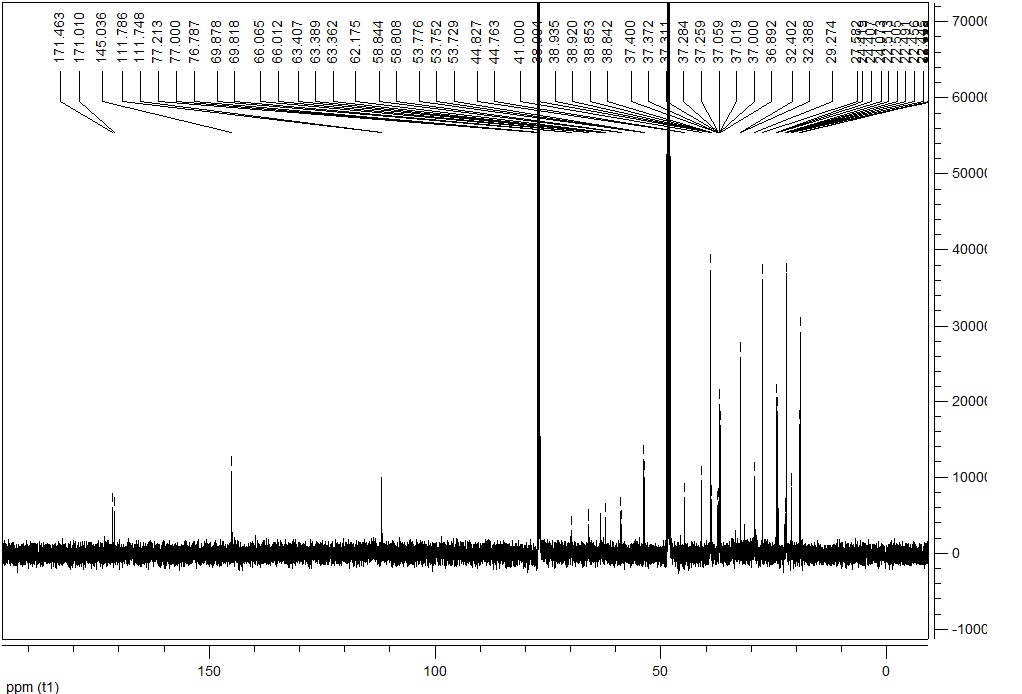

Supplement: S17 Fig — (DOCX) [file pone.0172238.s017.docx]

## S18 Fig. 31PNMR spectrum of 6d

**
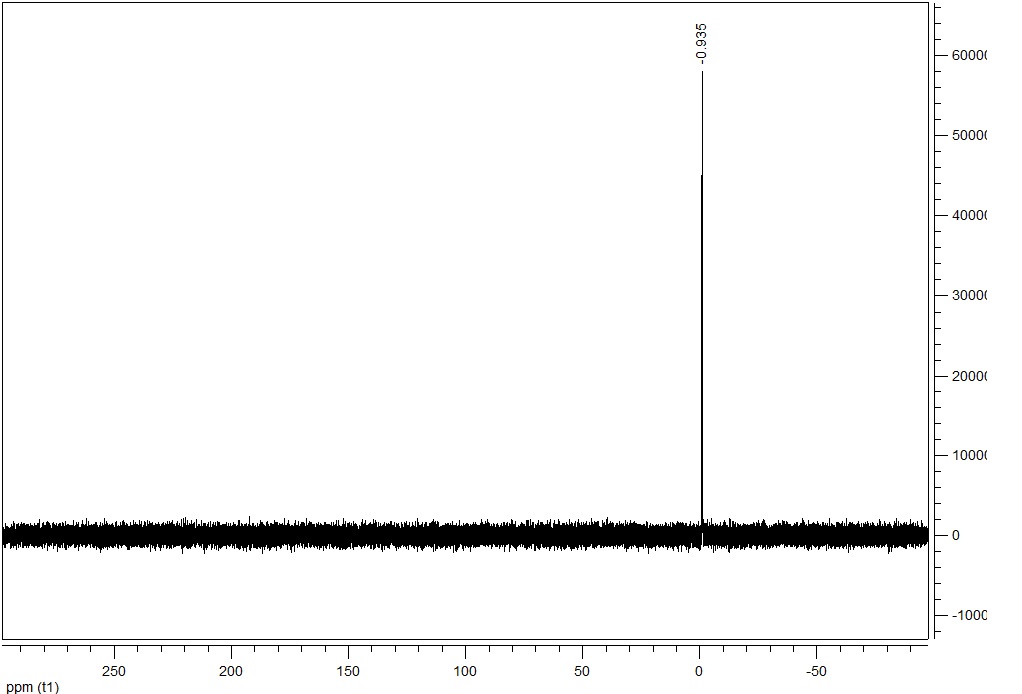
**

Supplement: S18 Fig — (DOCX) [file pone.0172238.s018.docx]

## S19 Fig. 1H – 1H COSY spectrum of 6d

**
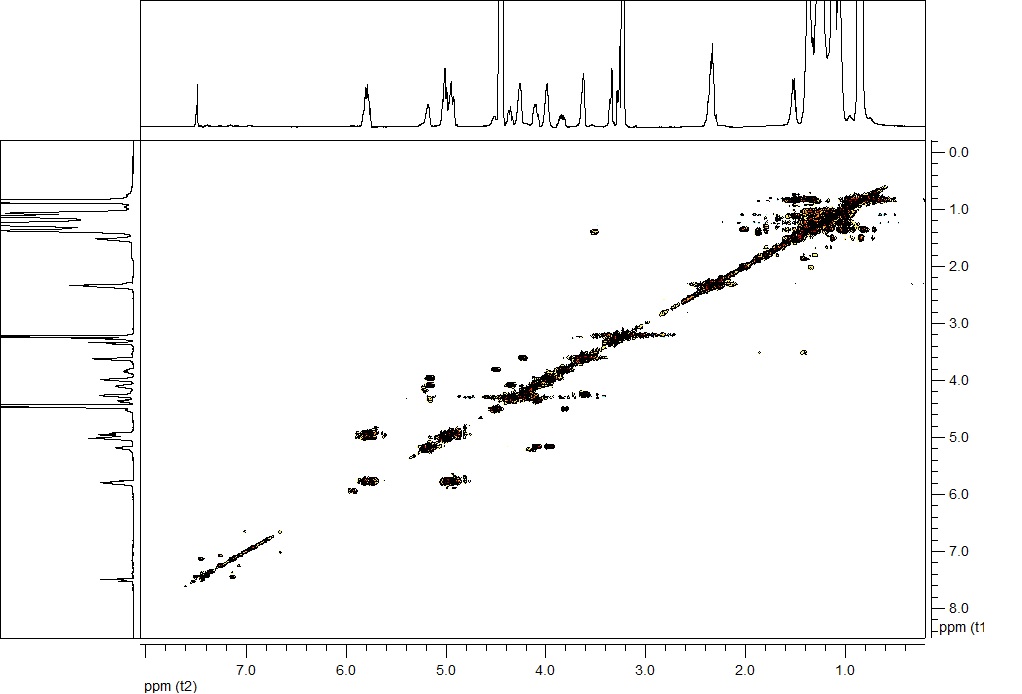
**

Supplement: S19 Fig — (DOCX) [file pone.0172238.s019.docx]

## S20 Fig. HSQC spectrum of 6d

**
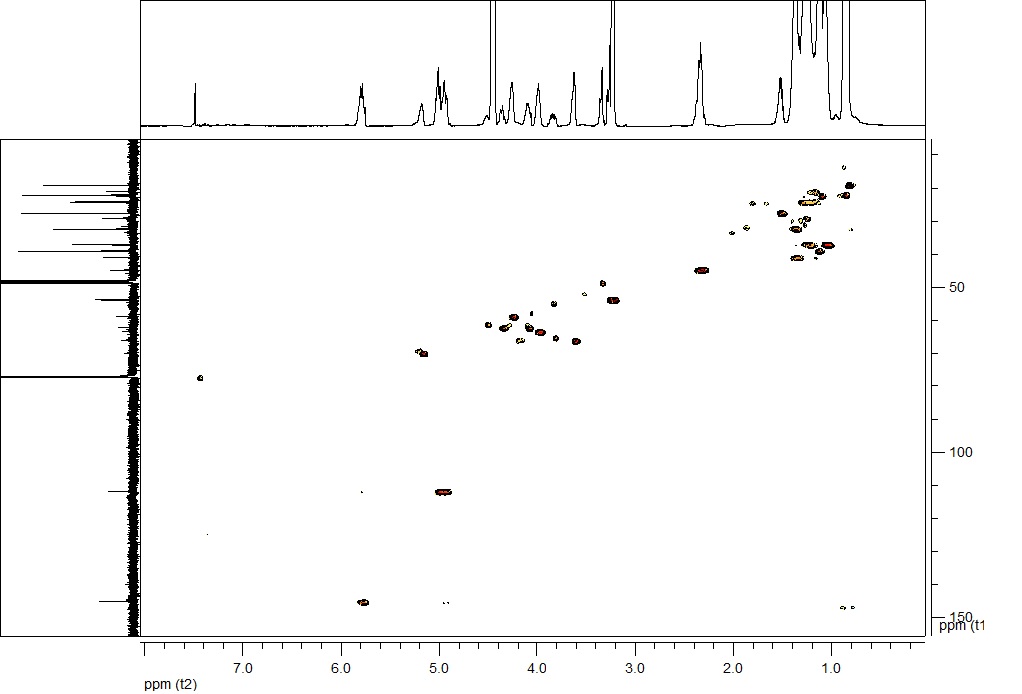
**

Supplement: S20 Fig — (DOCX) [file pone.0172238.s020.docx]

## S21 Fig. 1H NMR spectrum of 7a


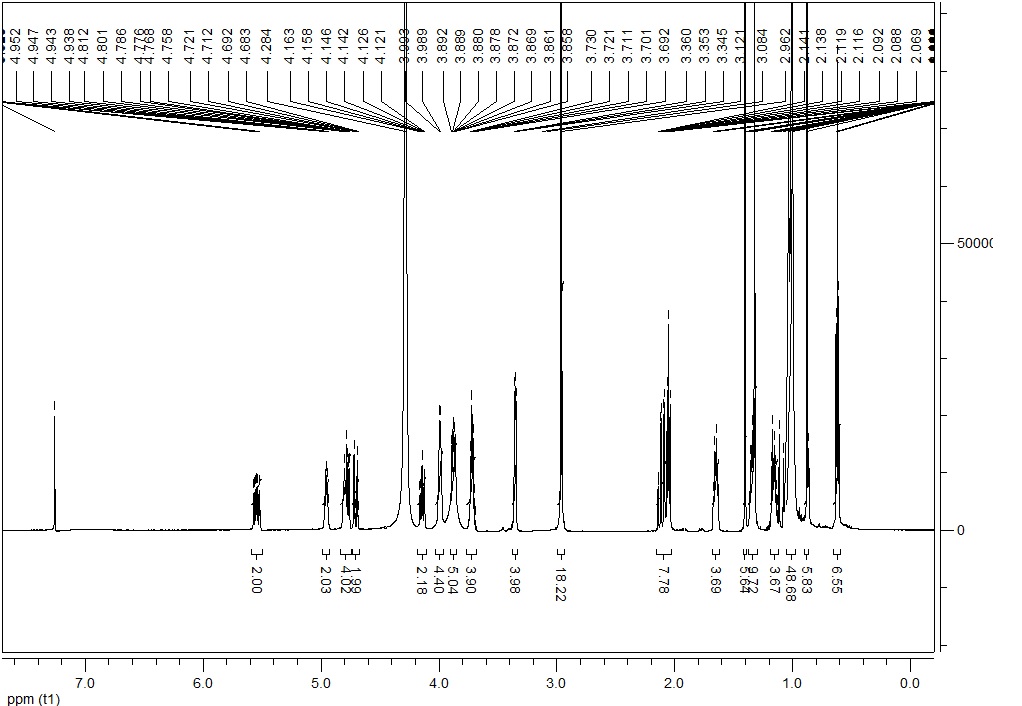

Supplement: S21 Fig — (DOCX) [file pone.0172238.s021.docx]

## S22 Fig. 13C NMR spectrum of 7a


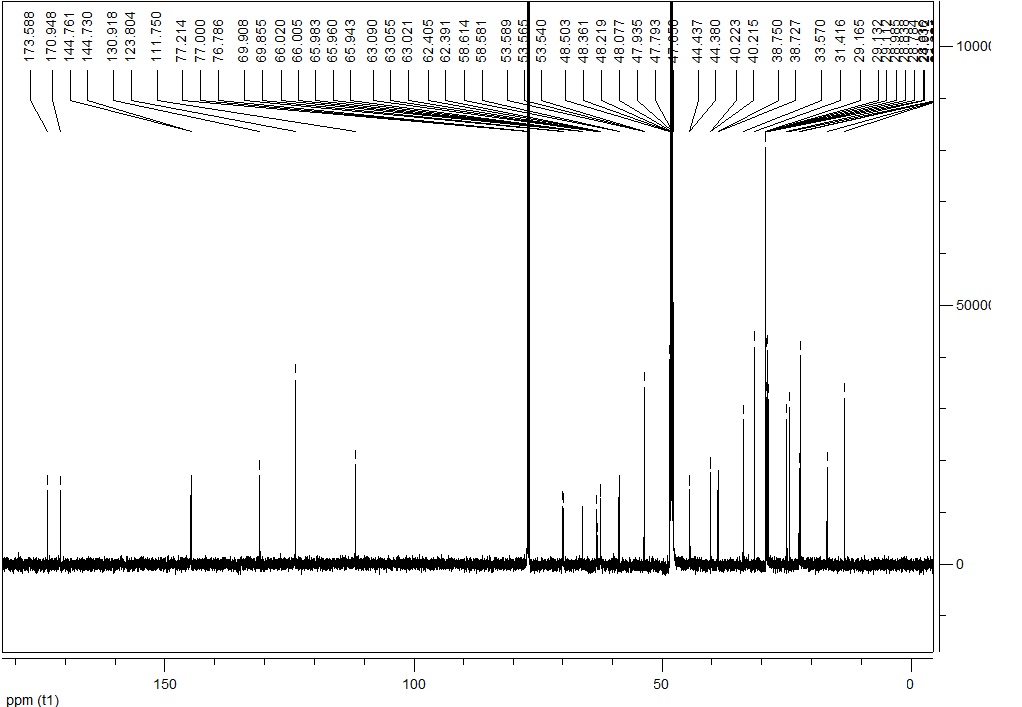

Supplement: S22 Fig — (DOCX) [file pone.0172238.s022.docx]

## S23 Fig. 31PNMR spectrum of 7a

**
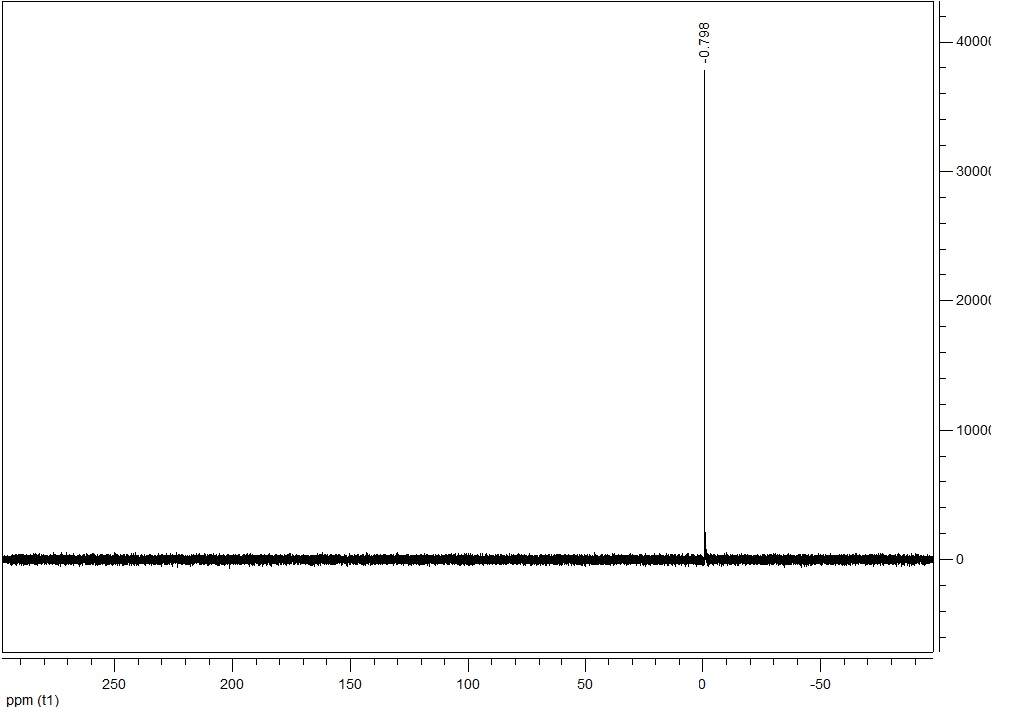
**

Supplement: S23 Fig — (DOCX) [file pone.0172238.s023.docx]

## S24 Fig. 1H – 1H COSY spectrum of 7a

**
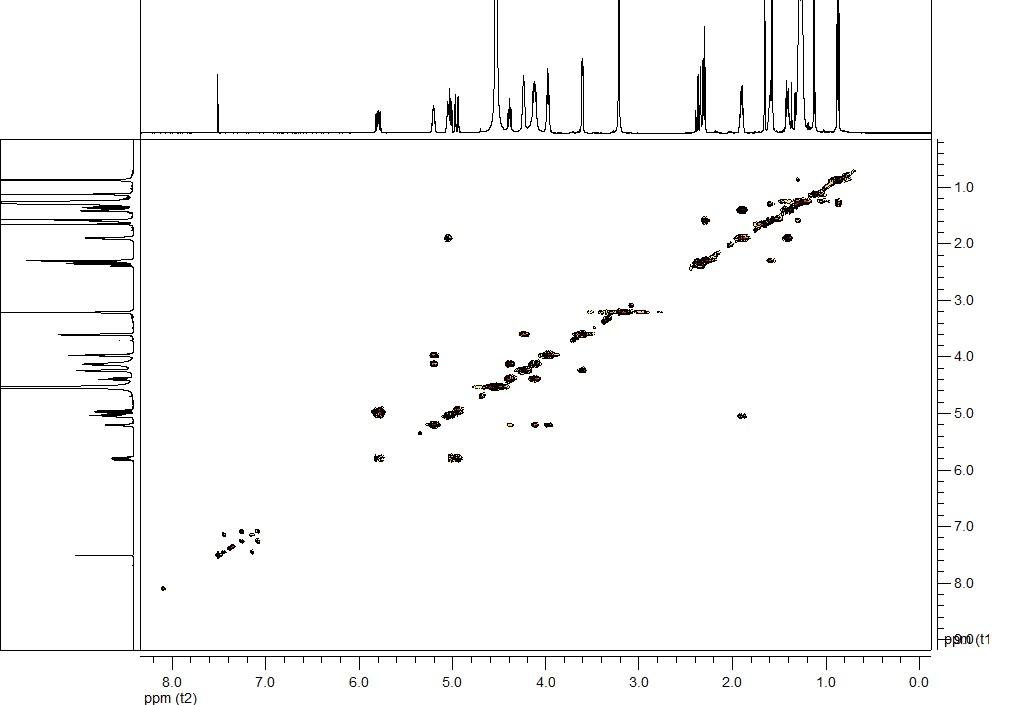
**

Supplement: S24 Fig — (DOCX) [file pone.0172238.s024.docx]

## S25 Fig. HSQC spectrum of 7a

**
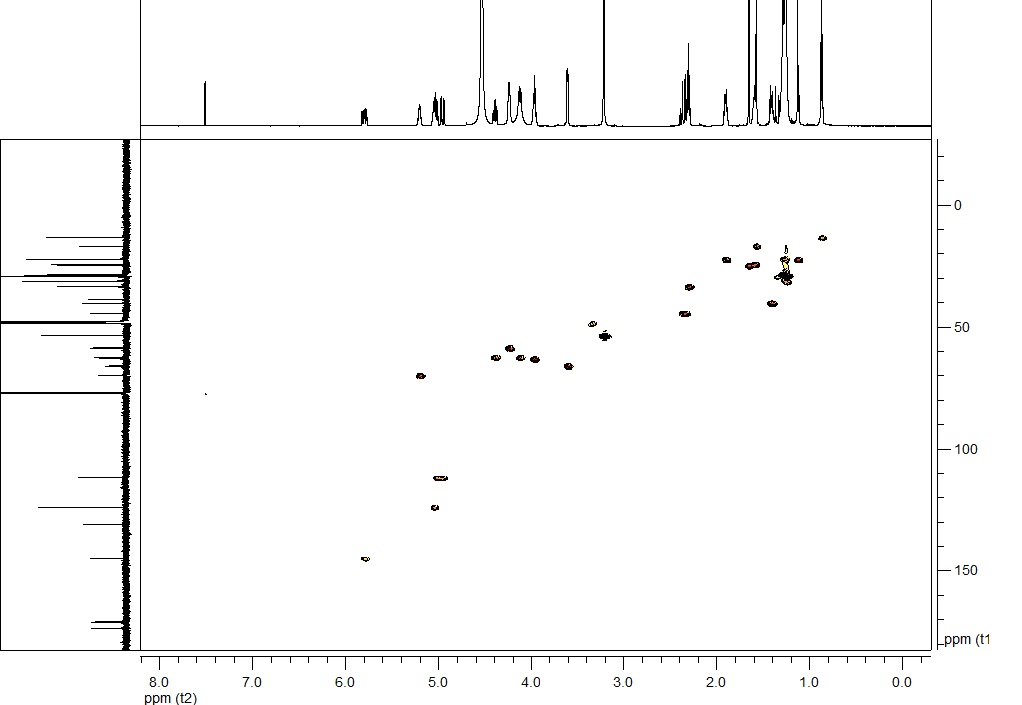
**

Supplement: S25 Fig — (DOCX) [file pone.0172238.s025.docx]

## S26 Fig. 1H NMR spectrum of 7b


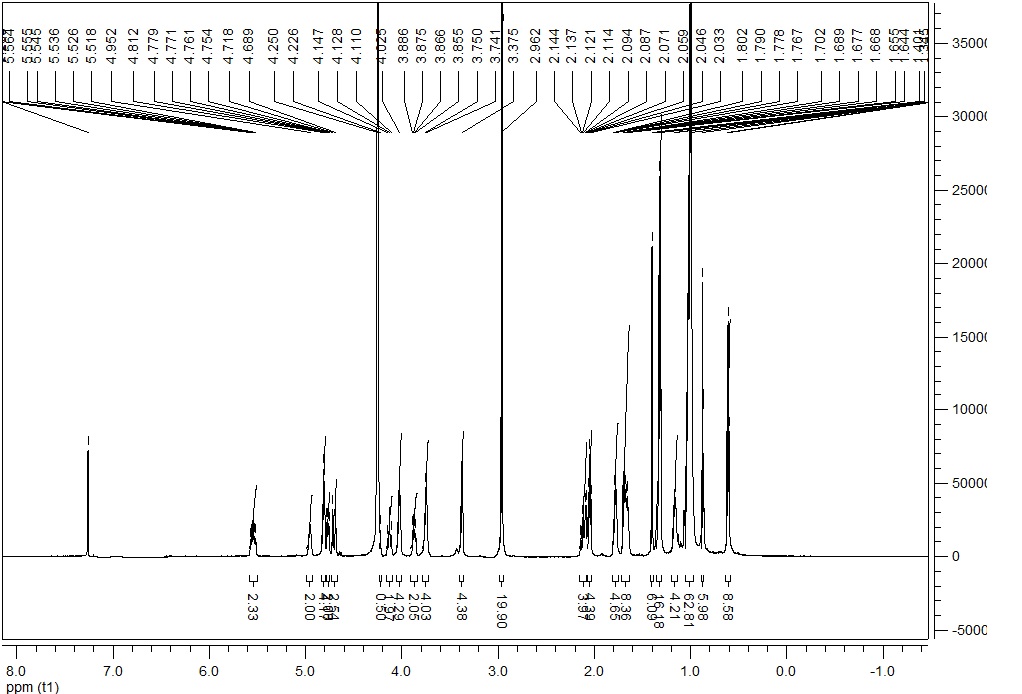

Supplement: S26 Fig — (DOCX) [file pone.0172238.s026.docx]

## S27 Fig. 13C NMR spectrum of 7b


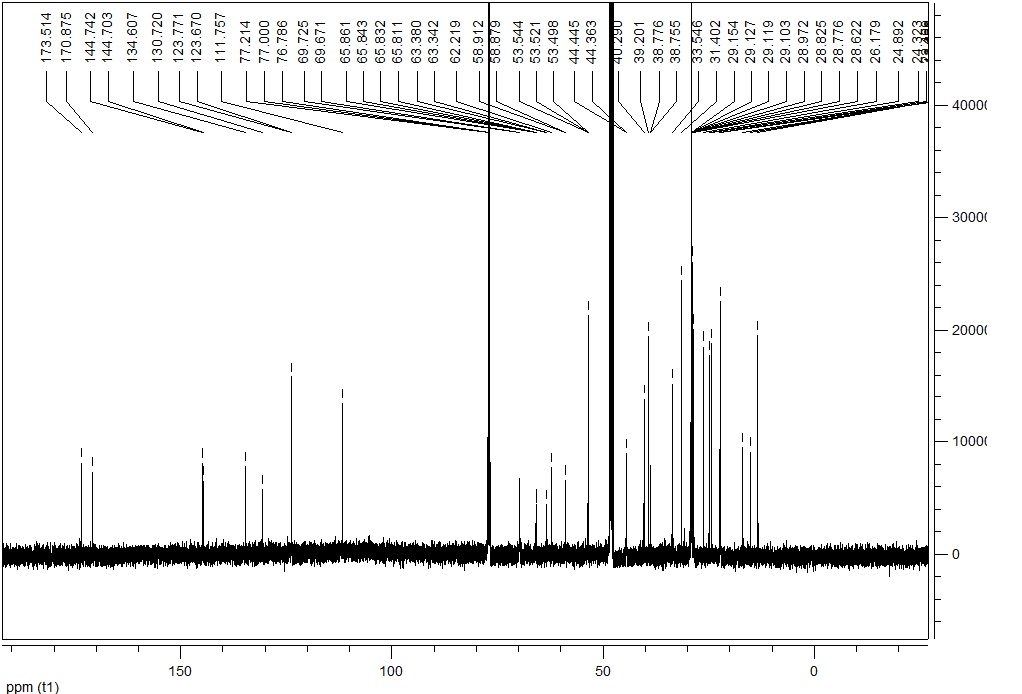

Supplement: S27 Fig — (DOCX) [file pone.0172238.s027.docx]

## S28 Fig. 31PNMR spectrum of 7b

##
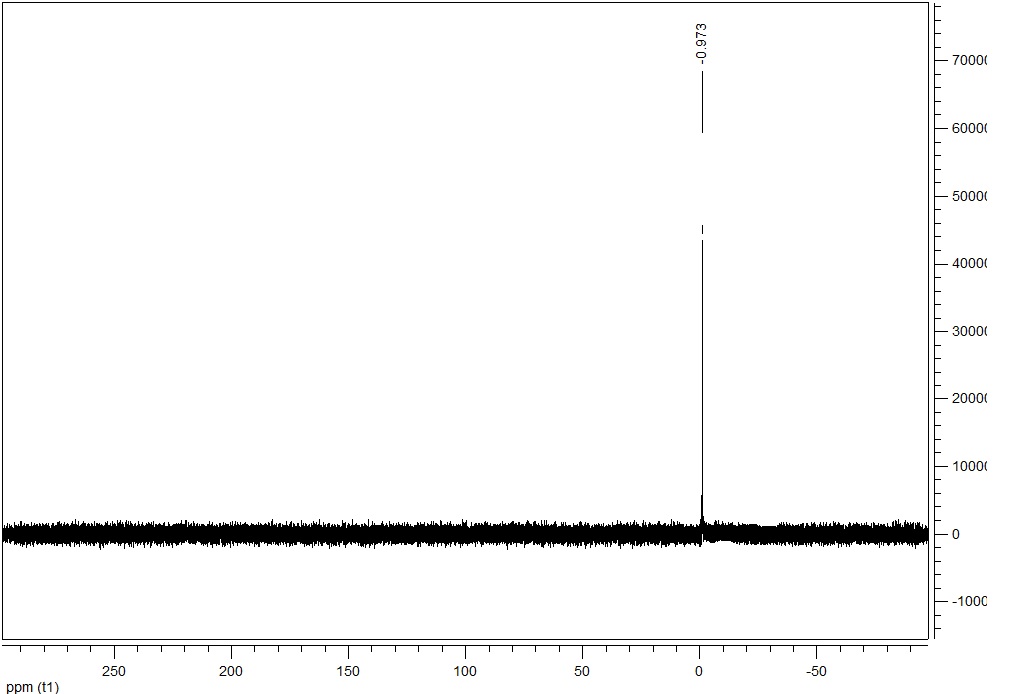

Supplement: S28 Fig — (DOCX) [file pone.0172238.s028.docx]

## S29 Fig. 1H – 1H COSY spectrum of 7b

##
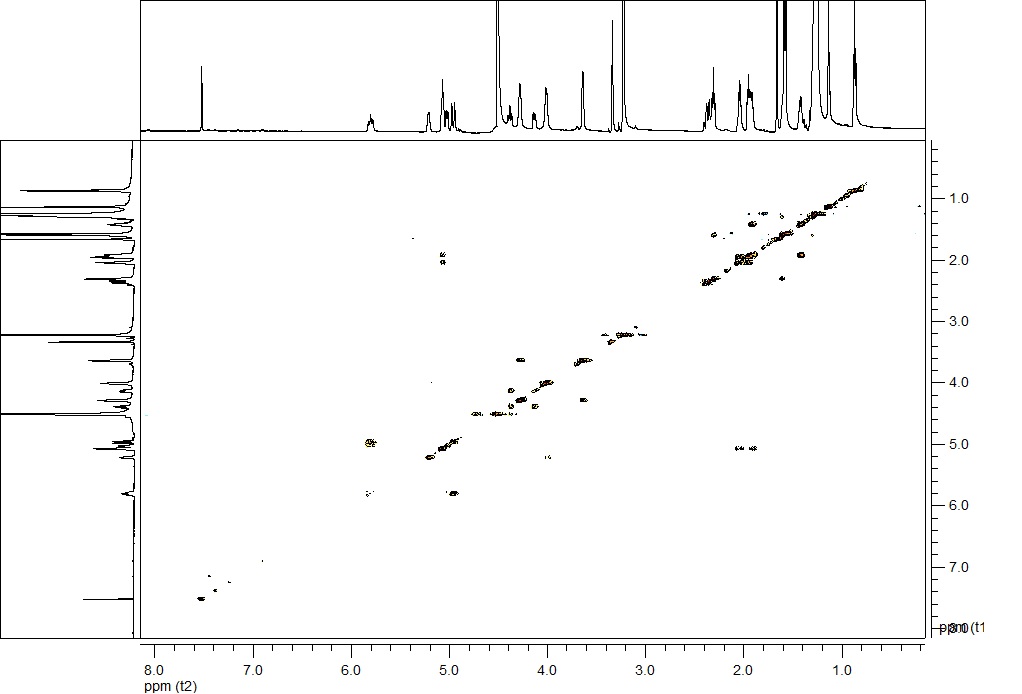

Supplement: S29 Fig — (DOCX) [file pone.0172238.s029.docx]

## S30 Fig. HSQC spectrum of 7b

**
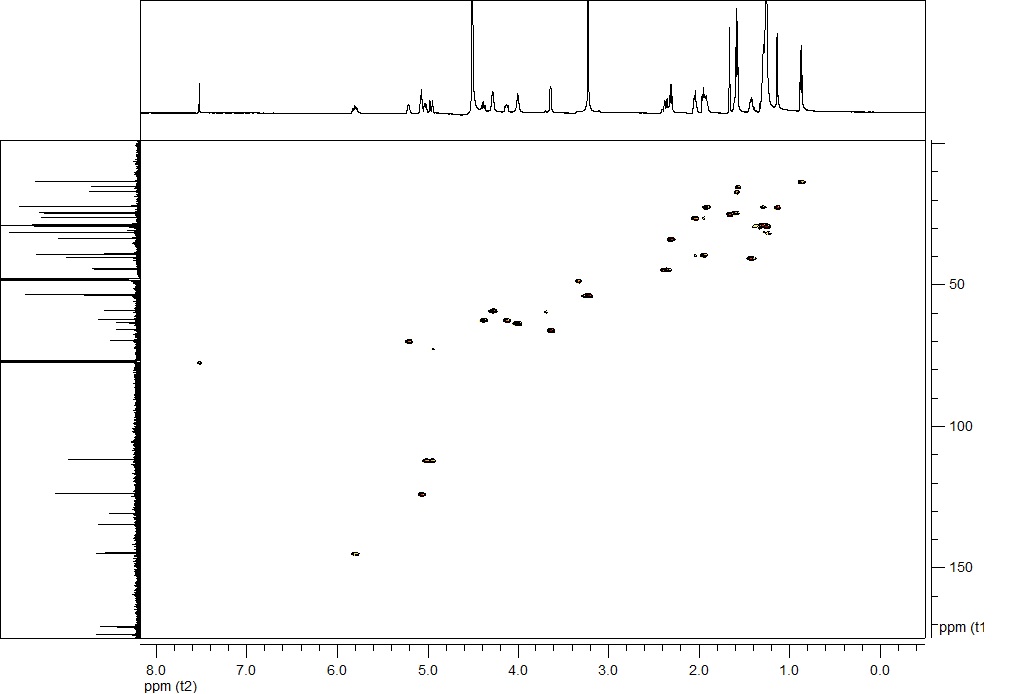
**

Supplement: S30 Fig — (DOCX) [file pone.0172238.s030.docx]

## S31 Fig. 1H NMR spectrum of 7c


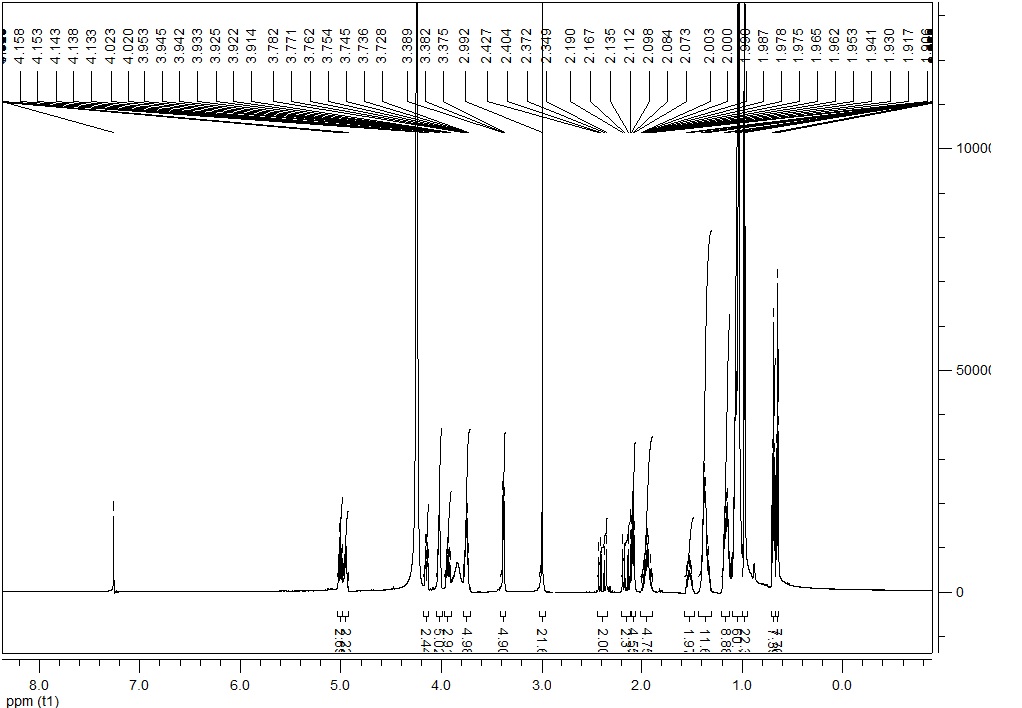

Supplement: S31 Fig — (DOCX) [file pone.0172238.s031.docx]

## S32 Fig. 13C NMR spectrum of 7c


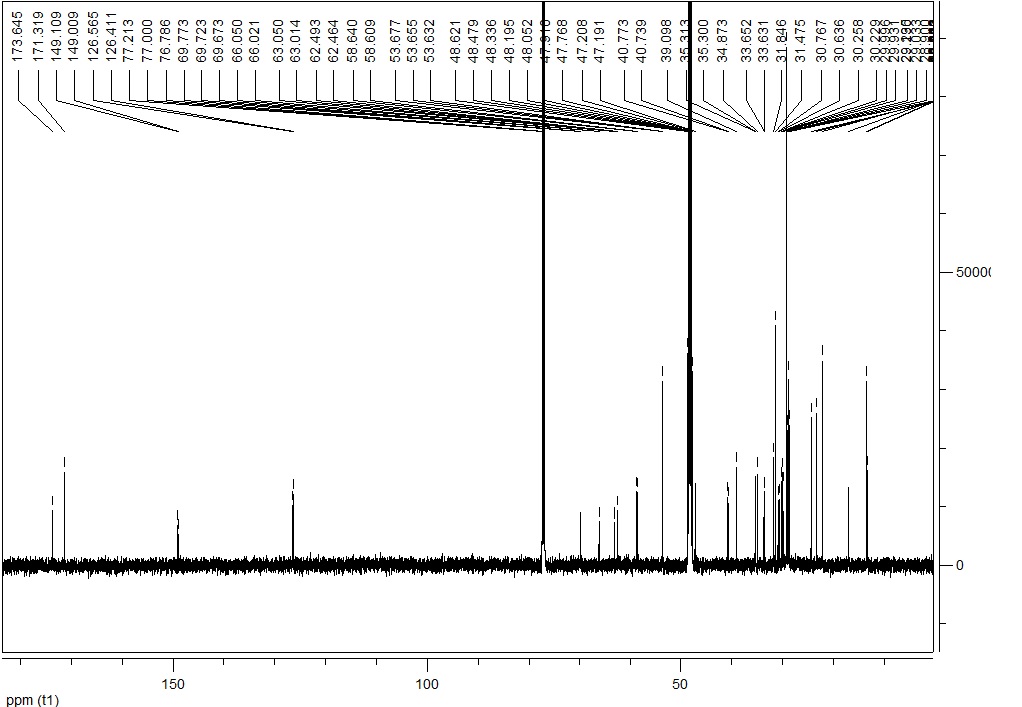

Supplement: S32 Fig — (DOCX) [file pone.0172238.s032.docx]

## S33 Fig. 31PNMR spectrum of 7c

**
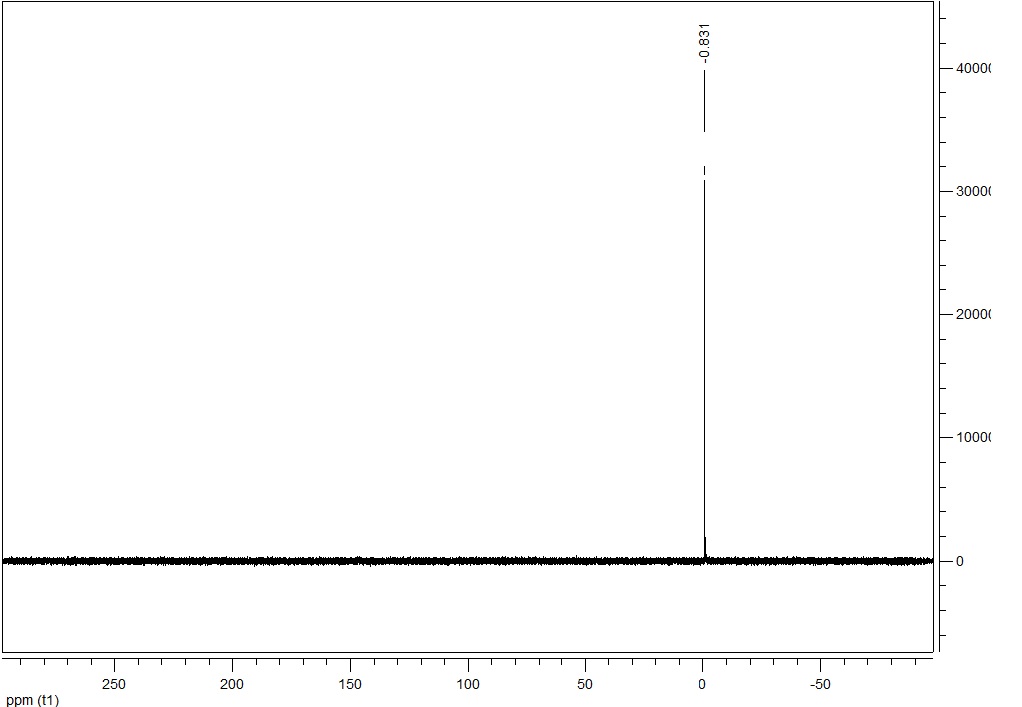
**

Supplement: S33 Fig — (DOCX) [file pone.0172238.s033.docx]

## S34 Fig. 1H – 1H COSY spectrum of 7c

**
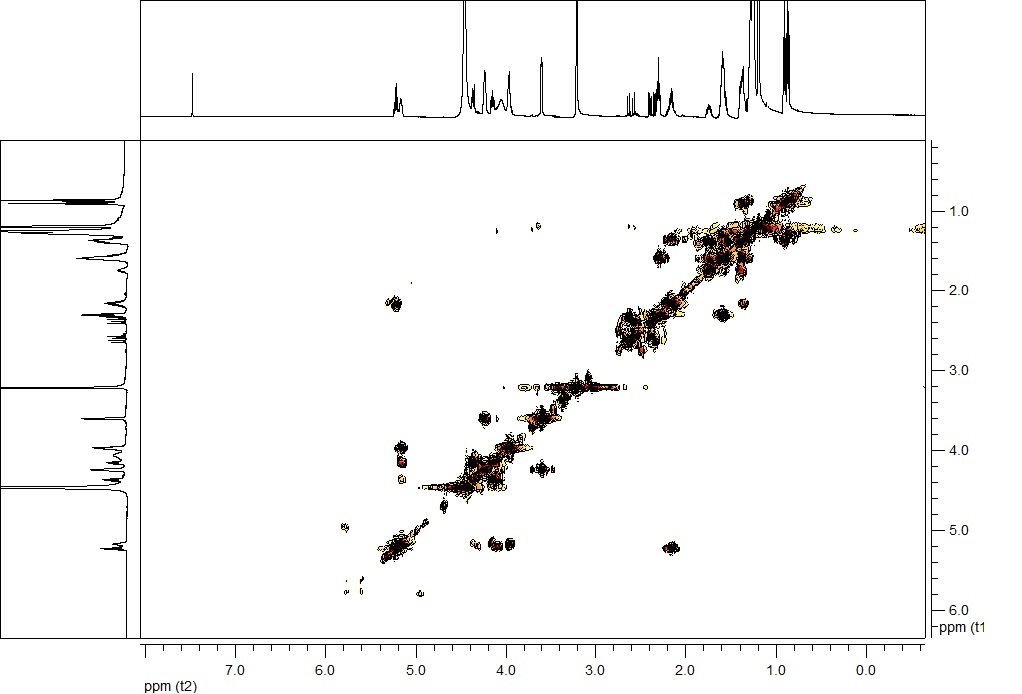
**

Supplement: S34 Fig — (DOCX) [file pone.0172238.s034.docx]

## S35 Fig. HSQC spectrum of 7c

**
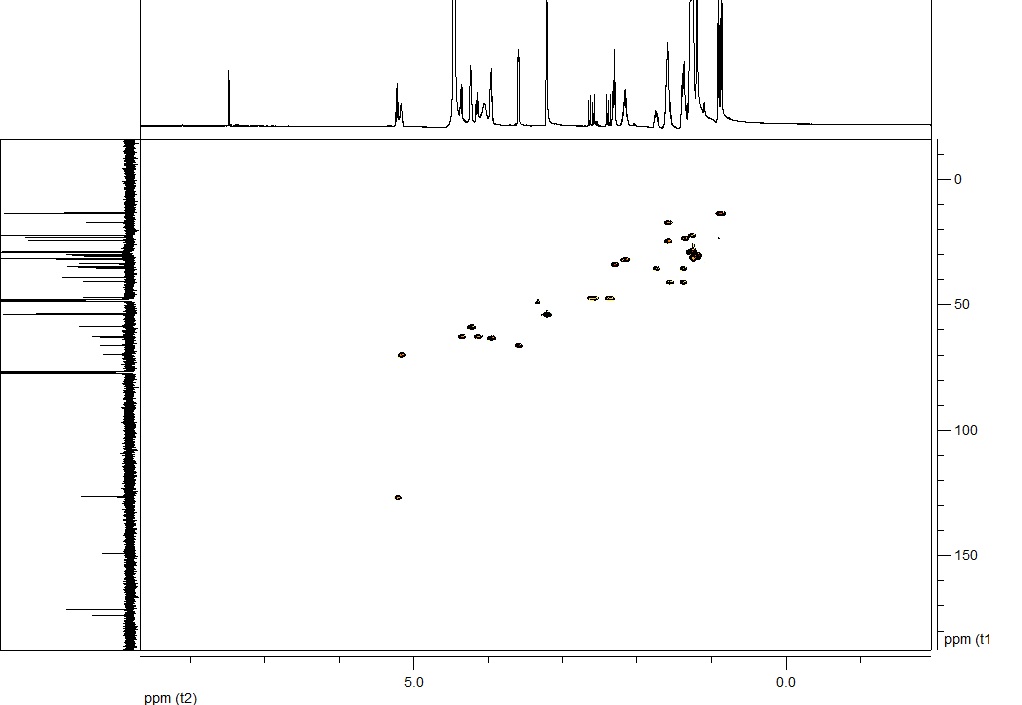
**

Supplement: S35 Fig — (DOCX) [file pone.0172238.s035.docx]

## S36 Fig. 1H NMR spectrum of 7d


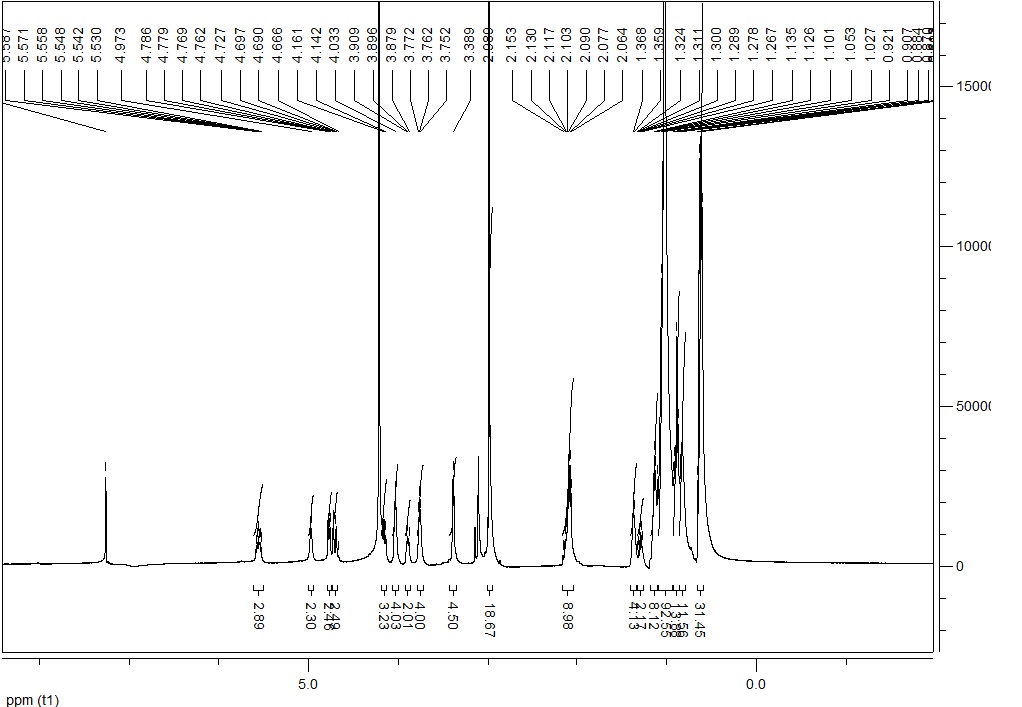

Supplement: S36 Fig — (DOCX) [file pone.0172238.s036.docx]

## S37 Fig. 13C NMR spectrum of 7d


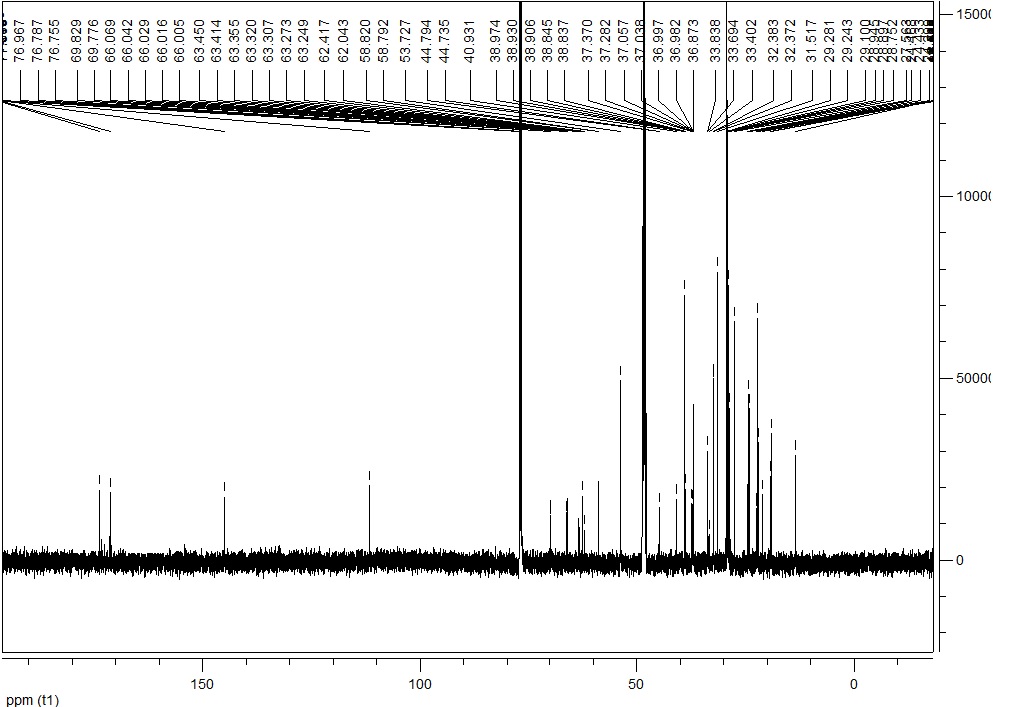

Supplement: S37 Fig — (DOCX) [file pone.0172238.s037.docx]

## S38 Fig. 31PNMR spectrum of 7d

**
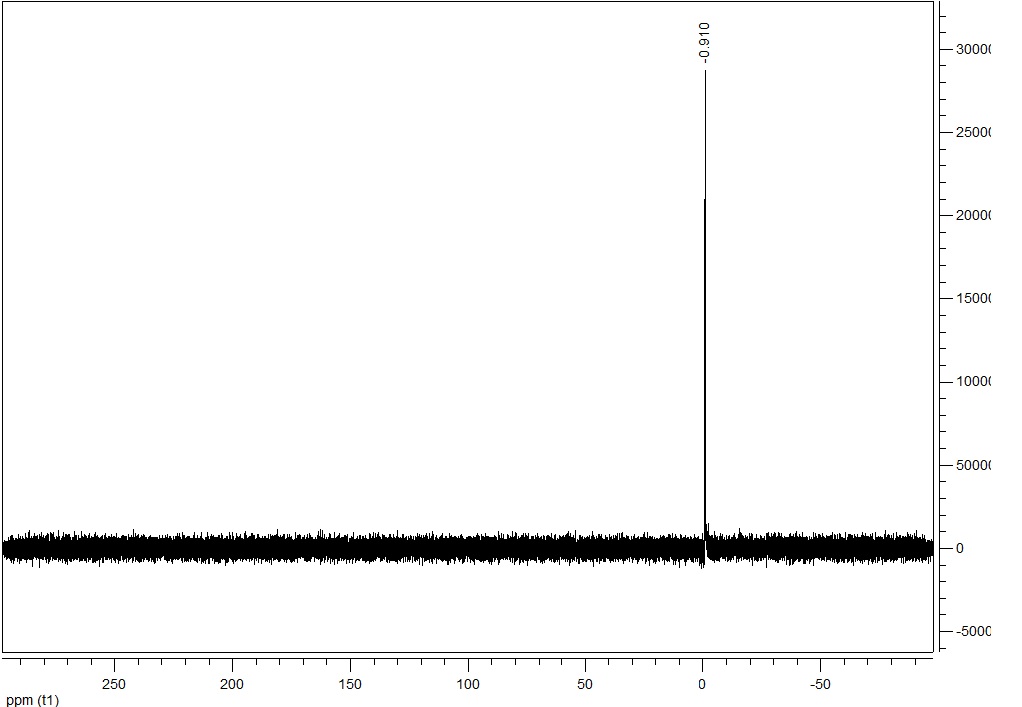
**

Supplement: S38 Fig — (DOCX) [file pone.0172238.s038.docx]

## S39 Fig. 1H – 1H COSY spectrum of 7d

**
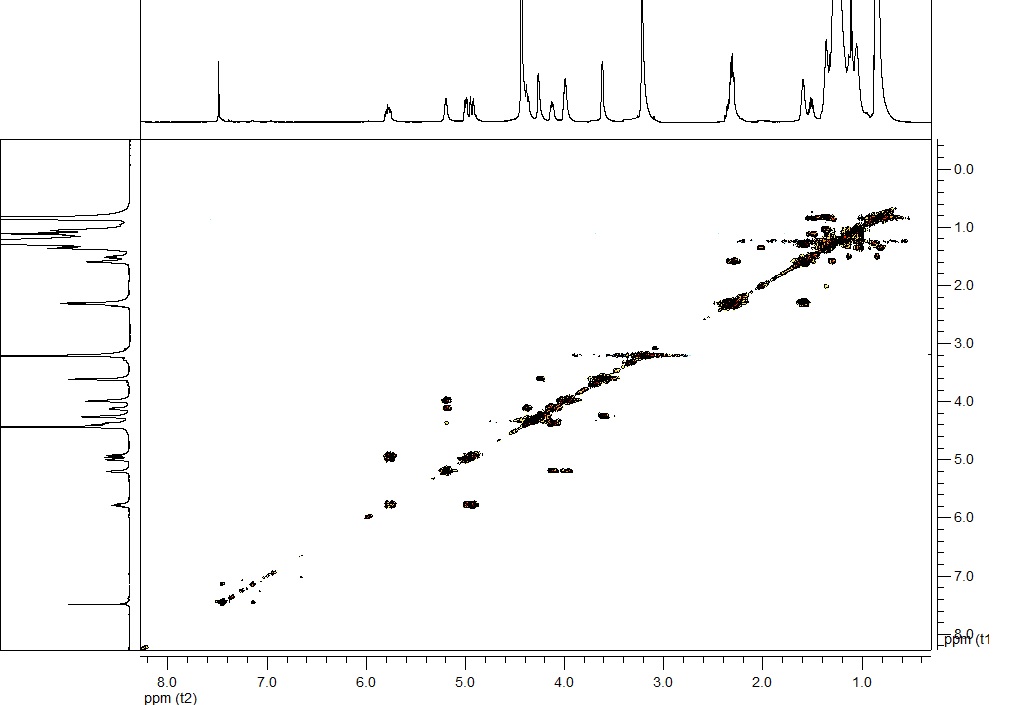
**

Supplement: S39 Fig — (DOCX) [file pone.0172238.s039.docx]

## S40 Fig. HSQC spectrum of 7d

**
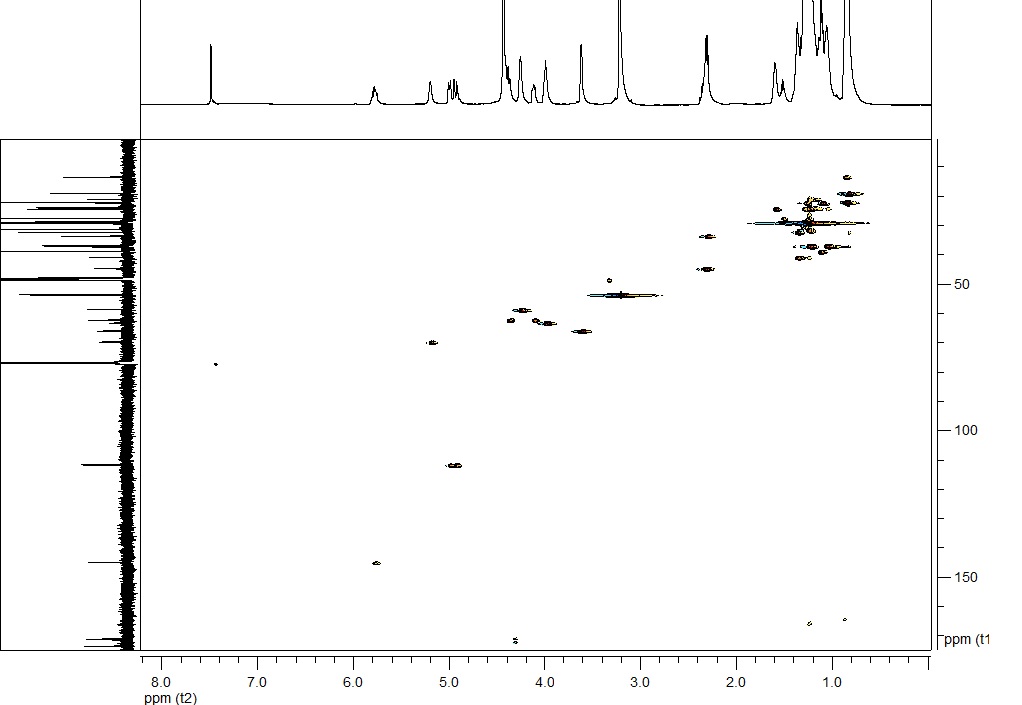
**

Supplement: S40 Fig — (DOCX) [file pone.0172238.s040.docx]

## S41 Fig. 1H NMR spectrum of 8a


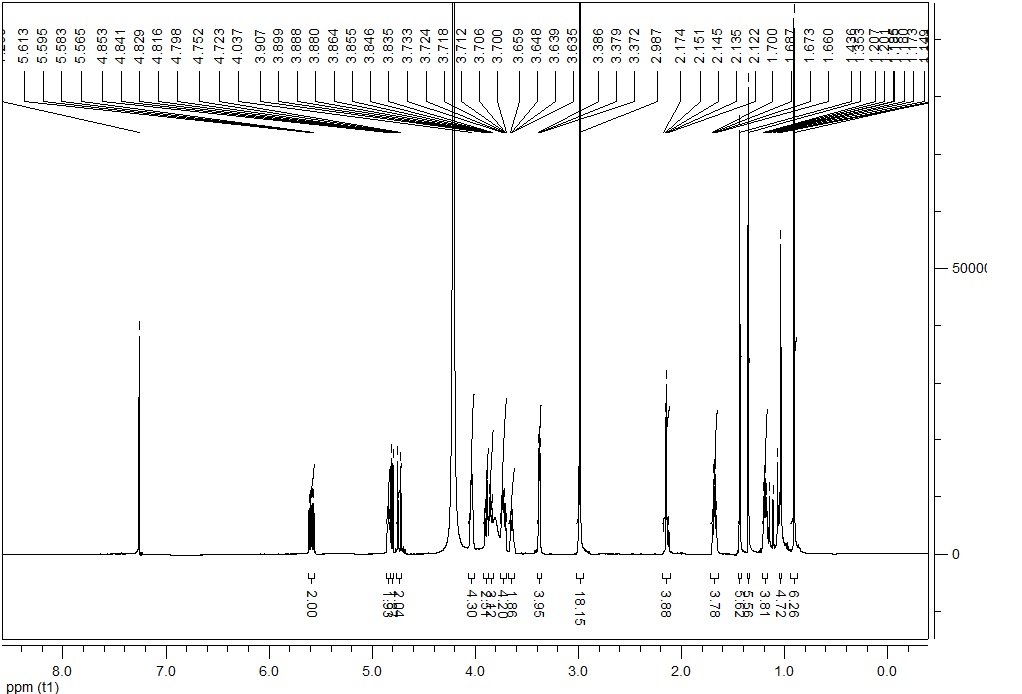

Supplement: S41 Fig — (DOCX) [file pone.0172238.s041.docx]

## S42 Fig. 13C NMR spectrum of 8a


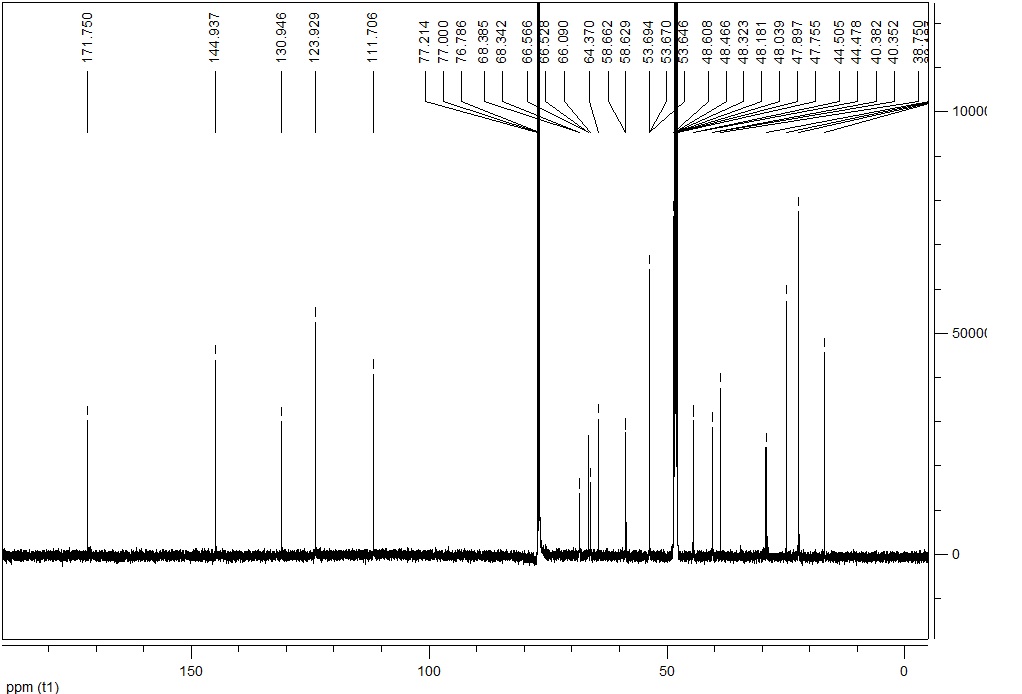

Supplement: S42 Fig — (DOCX) [file pone.0172238.s042.docx]

## S43 Fig. 31PNMR spectrum of 8a

**
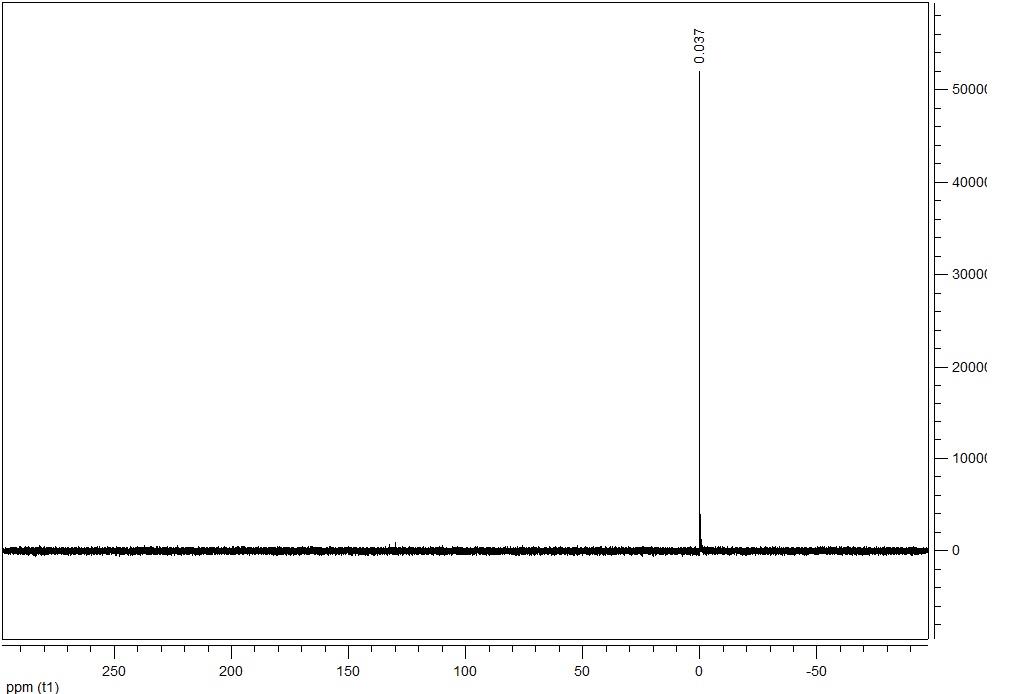
**

Supplement: S43 Fig — (DOCX) [file pone.0172238.s043.docx]

## S44 Fig. 1H – 1H COSY spectrum of 8a

**
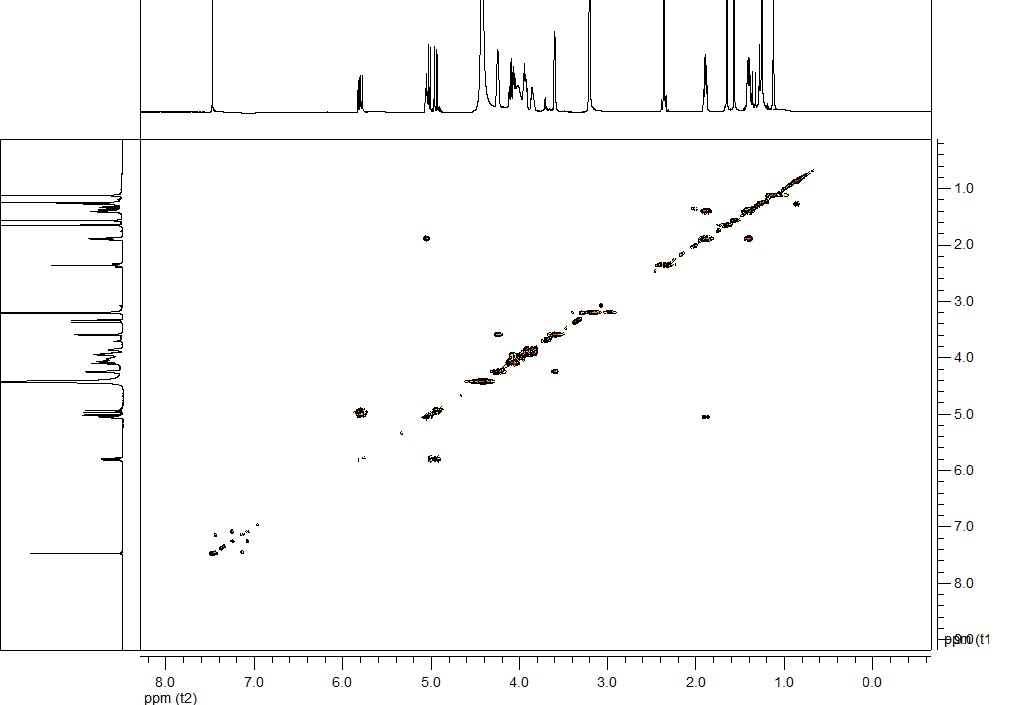
**

Supplement: S44 Fig — (DOCX) [file pone.0172238.s044.docx]

## S45 Fig. HSQC spectrum of 8a

**
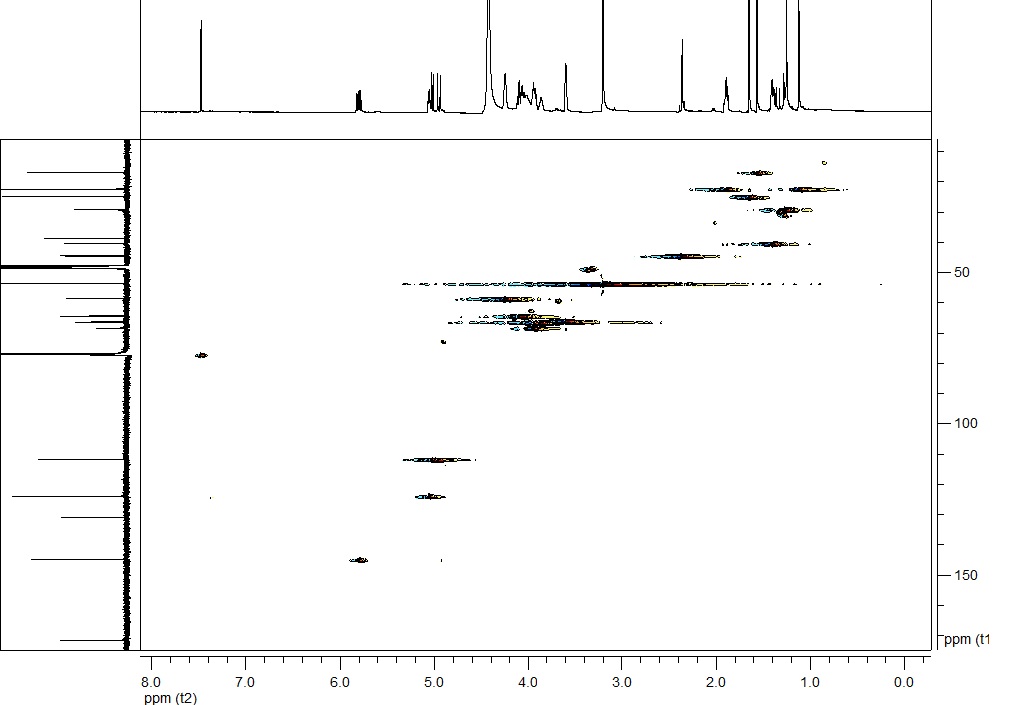
**

Supplement: S45 Fig — (DOCX) [file pone.0172238.s045.docx]

## S46 Fig. 1H NMR spectrum of 8b

##
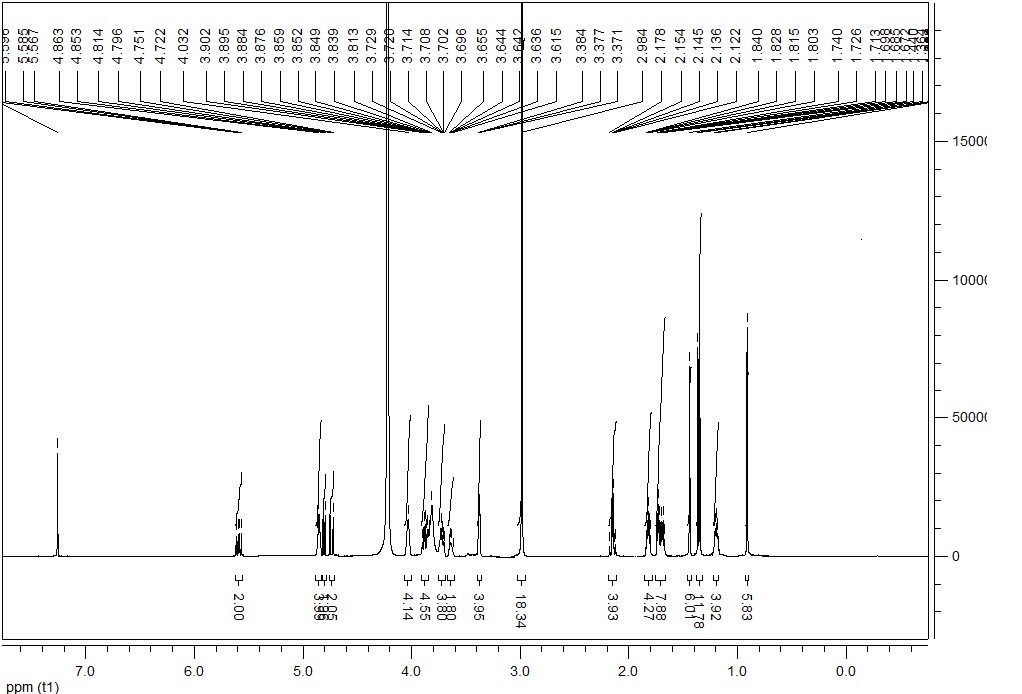

Supplement: S46 Fig — (DOCX) [file pone.0172238.s046.docx]

## S47 Fig. 13C NMR spectrum of 8b


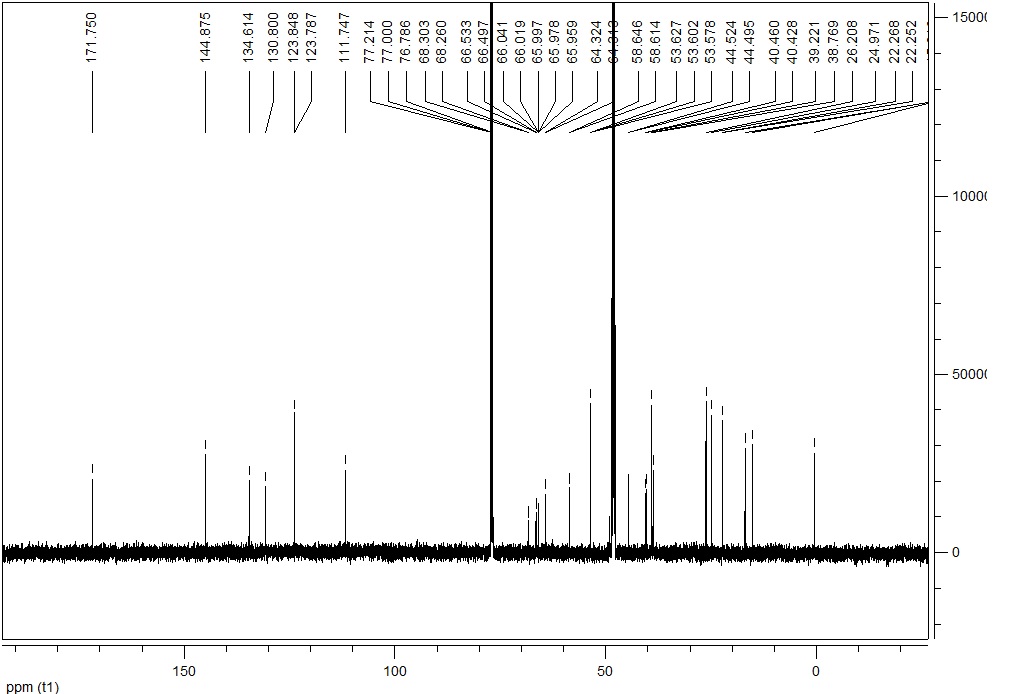

Supplement: S47 Fig — (DOCX) [file pone.0172238.s047.docx]

## S48 Fig. 31PNMR spectrum of 8b

**
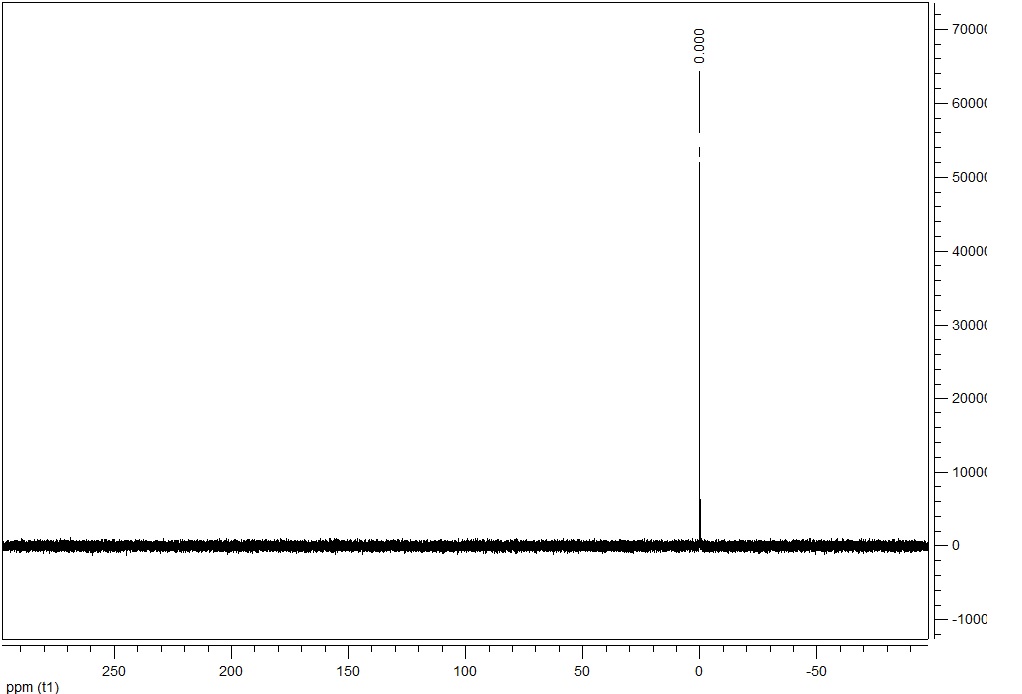
**

Supplement: S48 Fig — (DOCX) [file pone.0172238.s048.docx]

## S49 Fig. 1H – 1H COSY spectrum of 8b

##
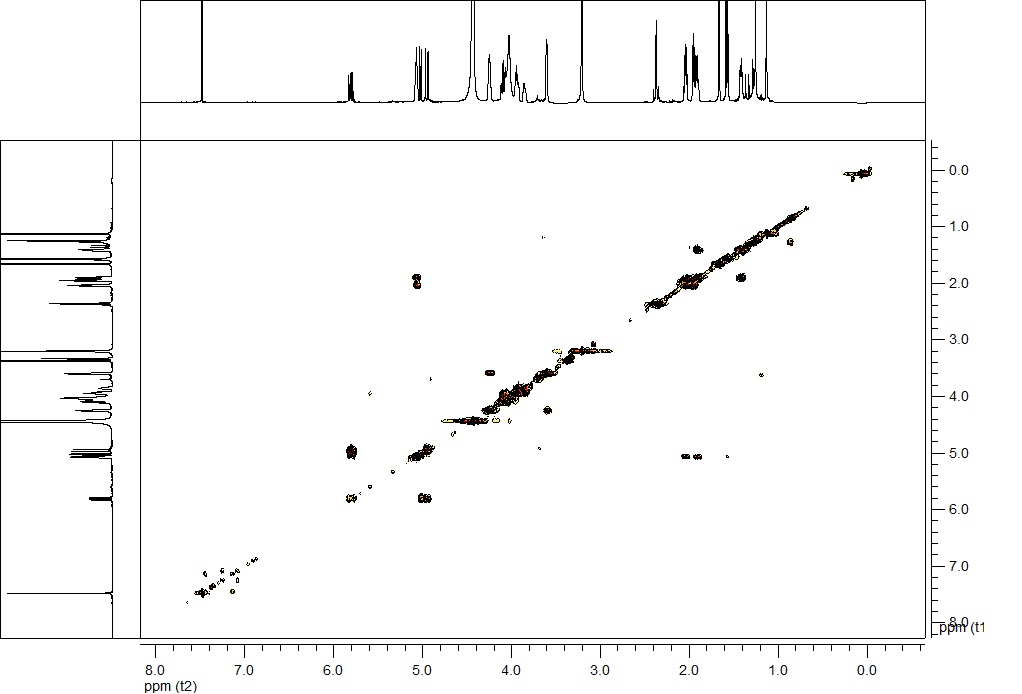

Supplement: S49 Fig — (DOCX) [file pone.0172238.s049.docx]

## S50 Fig. HSQC spectrum of 8b

**
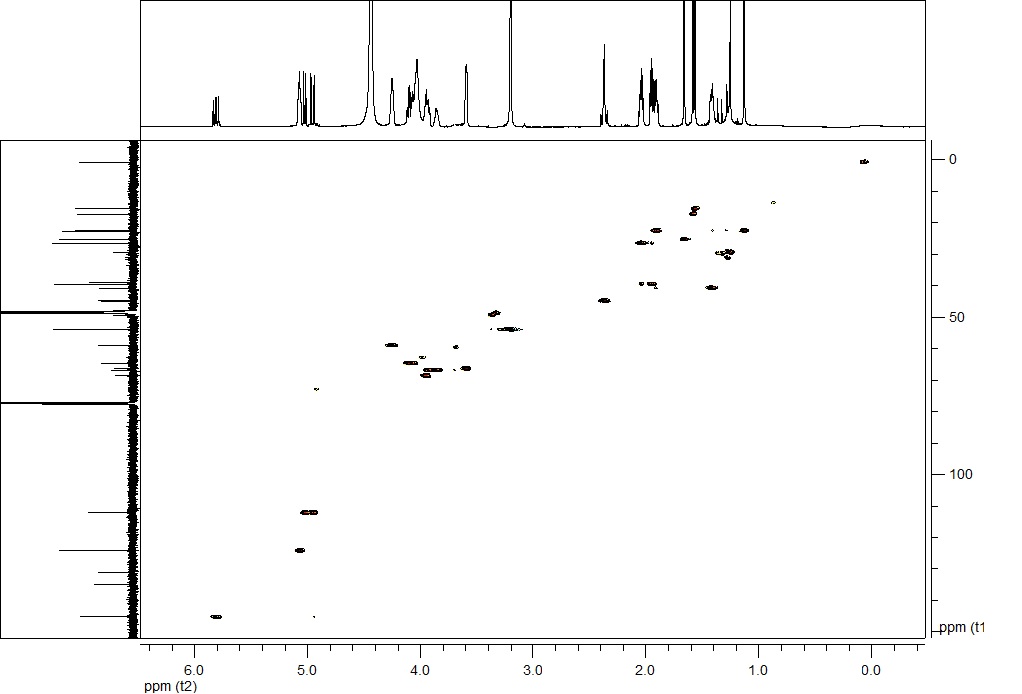
**

Supplement: S50 Fig — (DOCX) [file pone.0172238.s050.docx]

## S51 Fig. 1H NMR spectrum of 8c


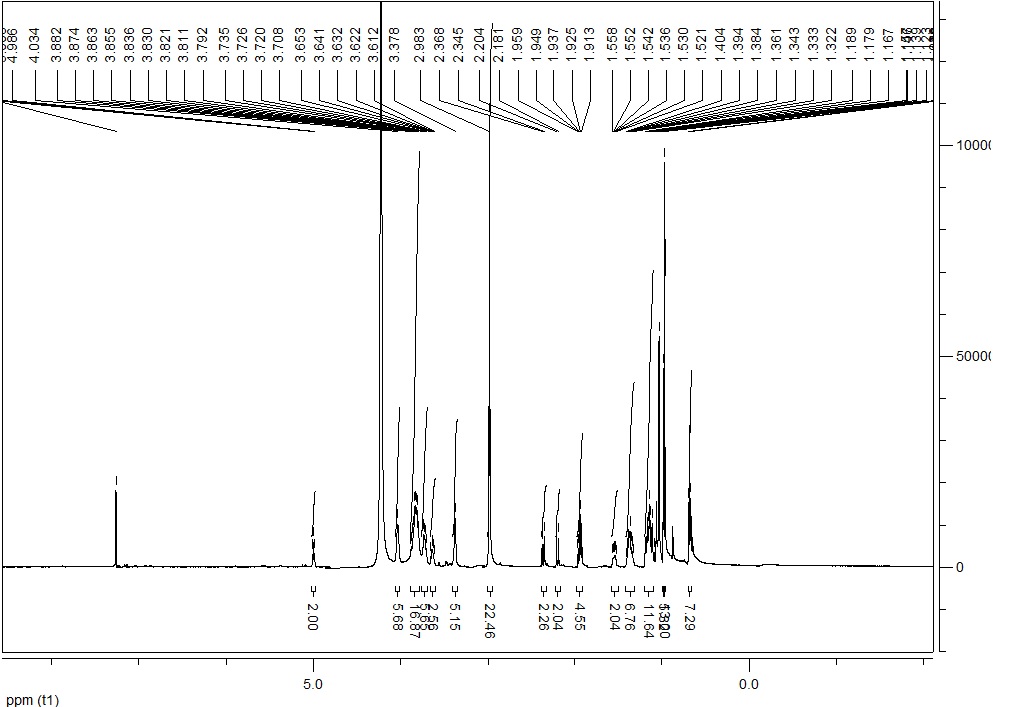

Supplement: S51 Fig — (DOCX) [file pone.0172238.s051.docx]

## S52 Fig. 13C NMR spectrum of 8c


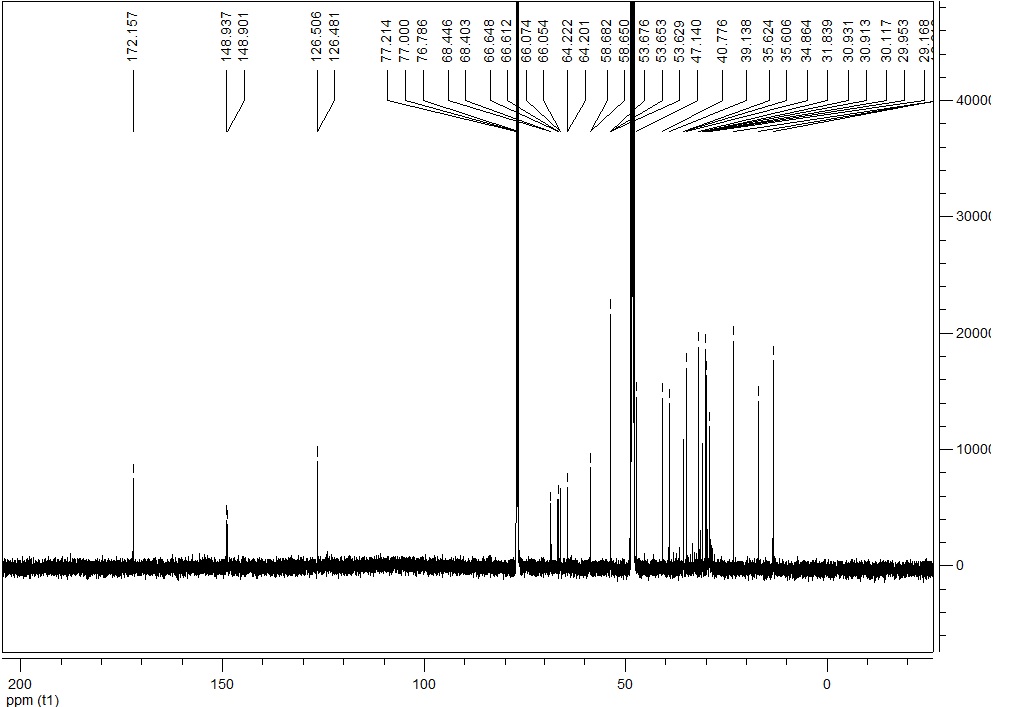

Supplement: S52 Fig — (DOCX) [file pone.0172238.s052.docx]

## S53 Fig. 31PNMR spectrum of 8c

**
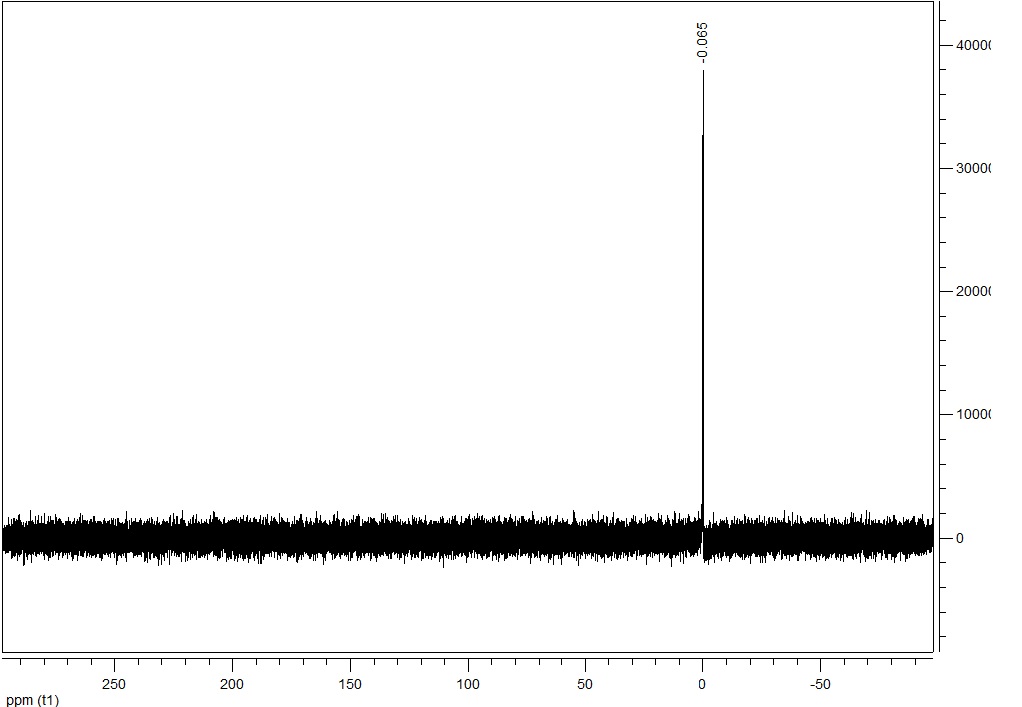
**

Supplement: S53 Fig — (DOCX) [file pone.0172238.s053.docx]

## S54 Fig. 1H – 1H COSY spectrum of 8c

##
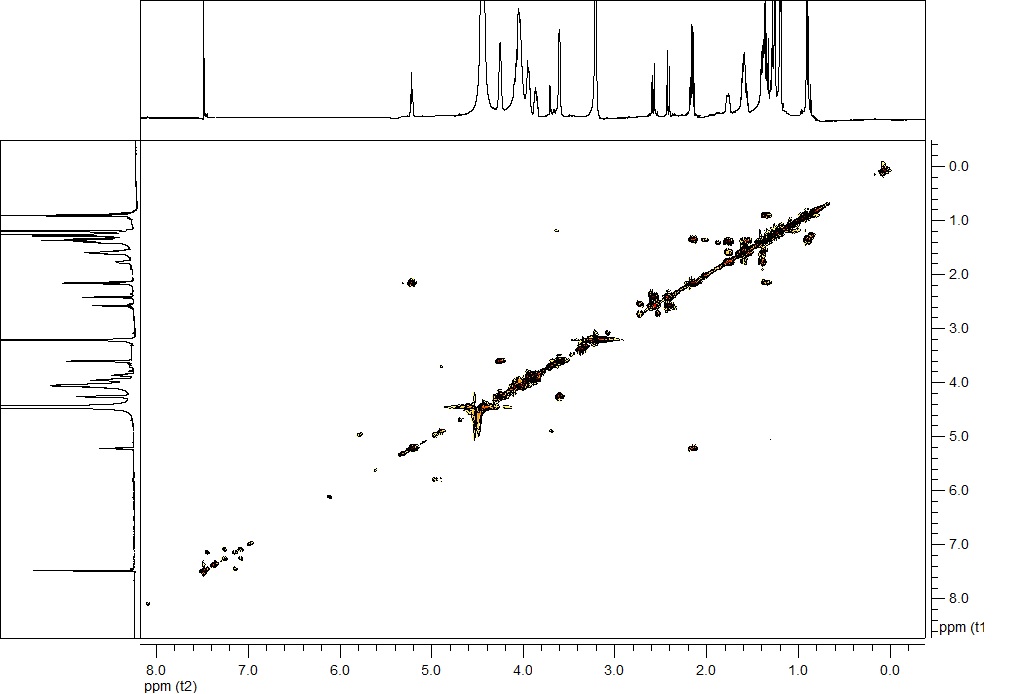

Supplement: S54 Fig — (DOCX) [file pone.0172238.s054.docx]

## S55 Fig. HSQC spectrum of 8c

**
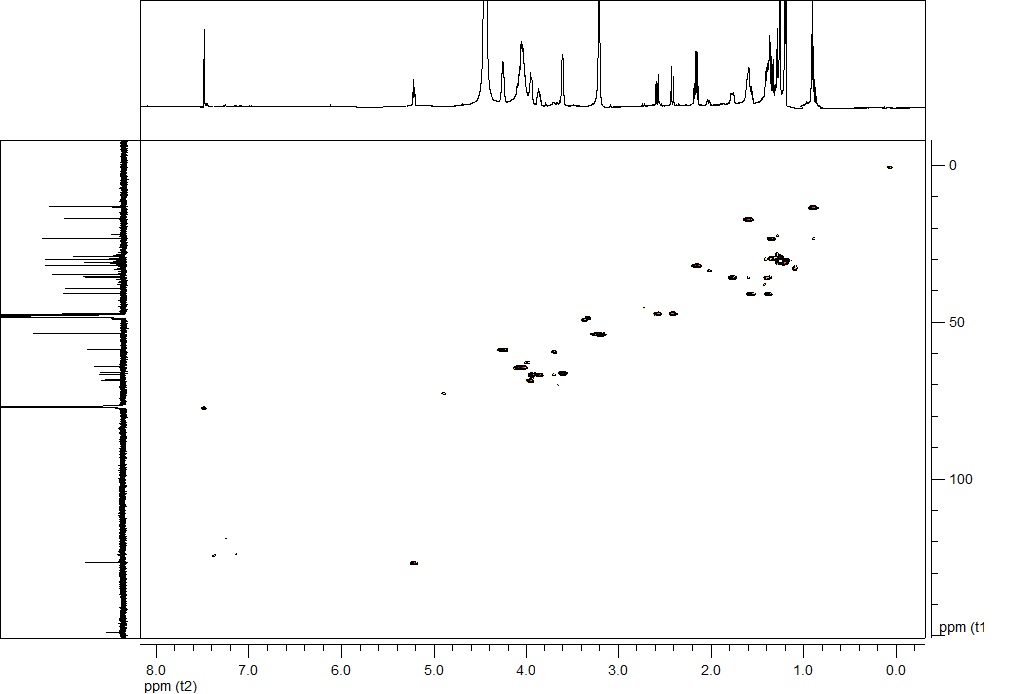
**

Supplement: S55 Fig — (DOCX) [file pone.0172238.s055.docx]

## S56 Fig. 1H NMR spectrum of 8d


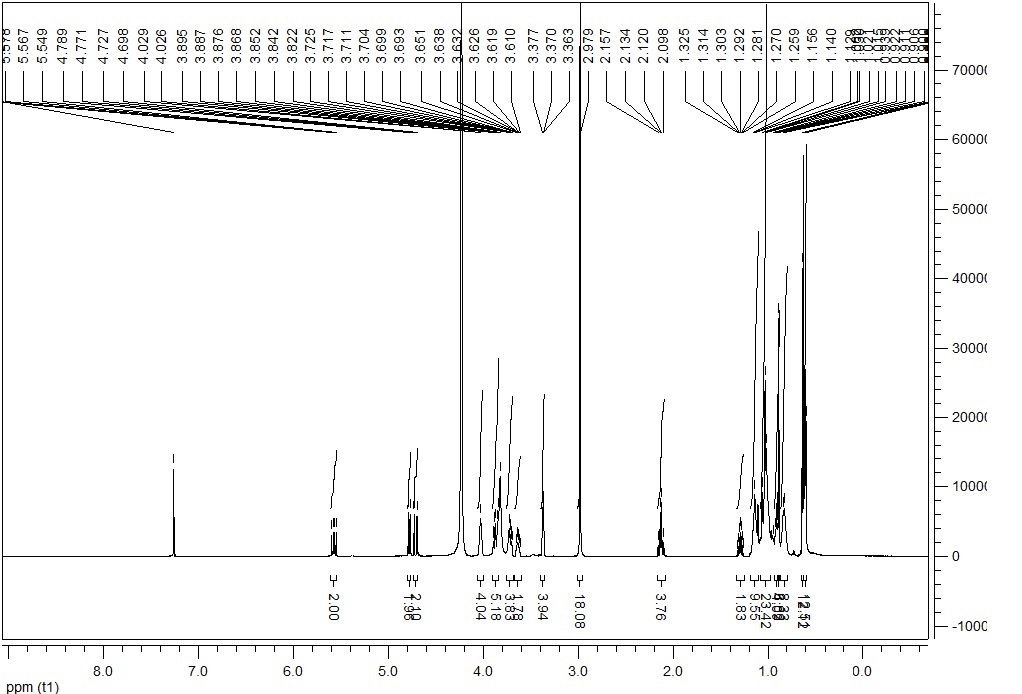

Supplement: S56 Fig — (DOCX) [file pone.0172238.s056.docx]

## S57 Fig. 13C NMR spectrum of 8d

##
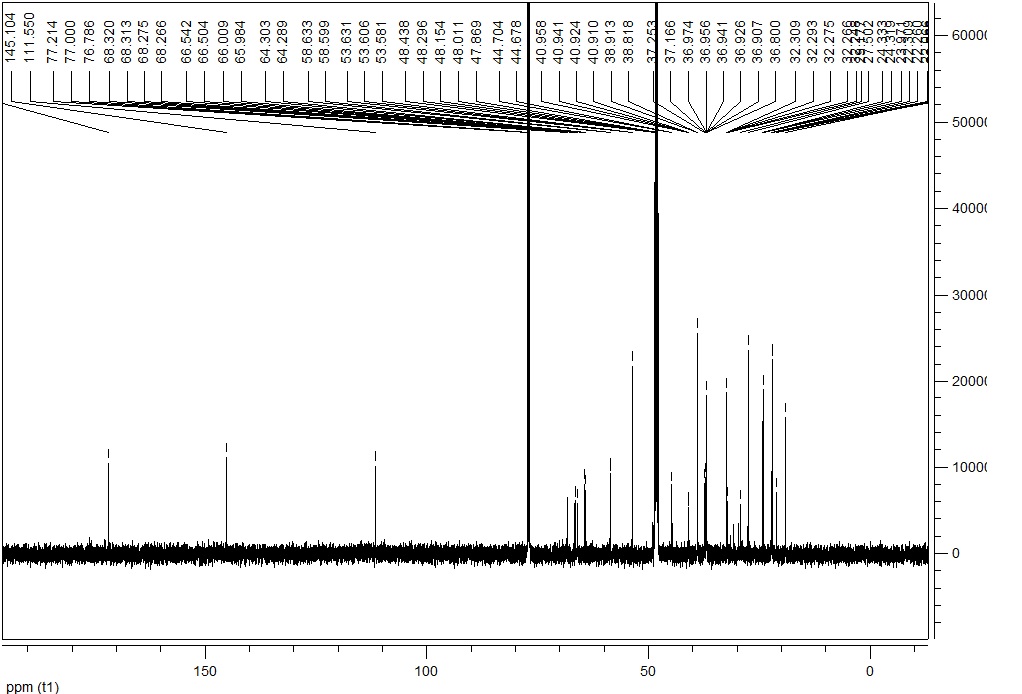

Supplement: S57 Fig — (DOCX) [file pone.0172238.s057.docx]

## S59 Fig. 1H – 1H COSY spectrum of 8d

**
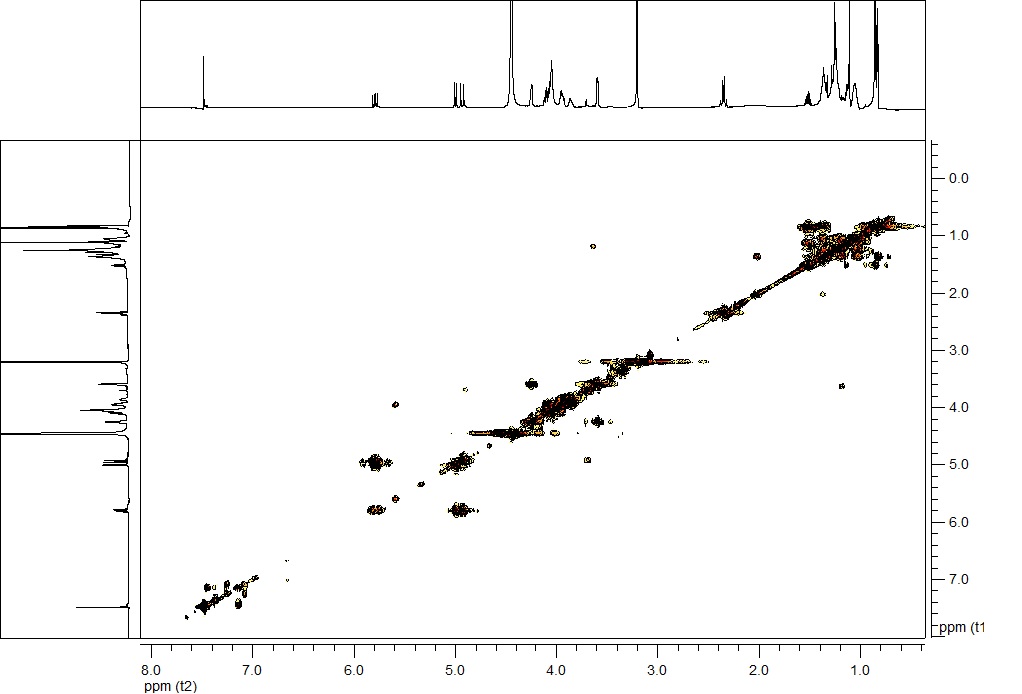
**

Supplement: S59 Fig — (DOCX) [file pone.0172238.s059.docx]

## S60 Fig. HSQC spectrum of 8d

**
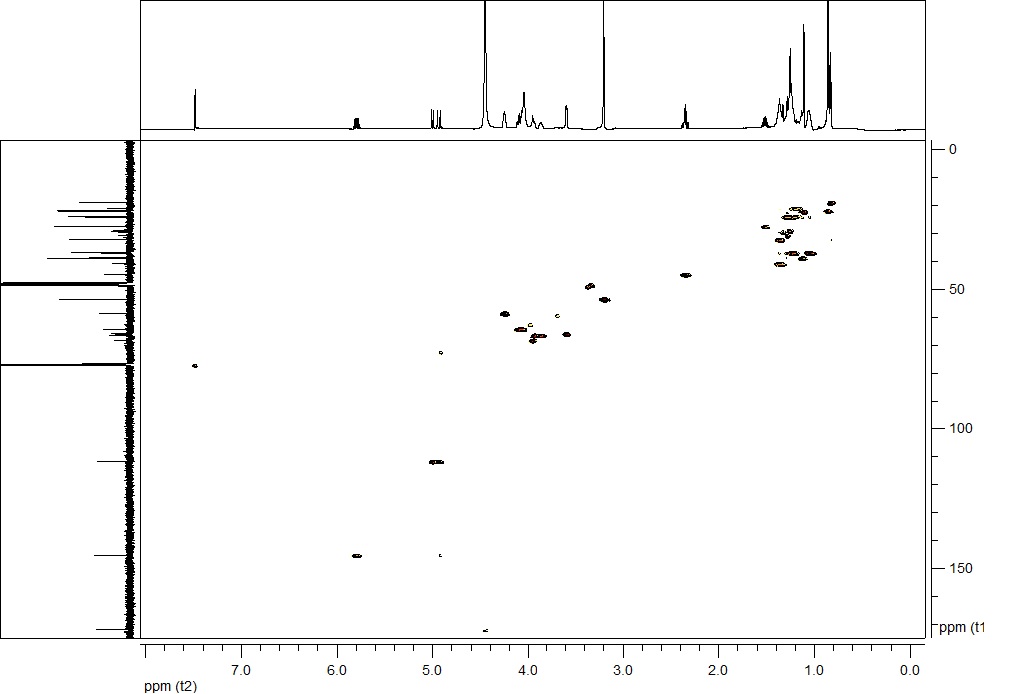
**

Supplement: S60 Fig — (DOCX) [file pone.0172238.s060.docx]

## S61 Fig. 1H NMR spectrum of 9a


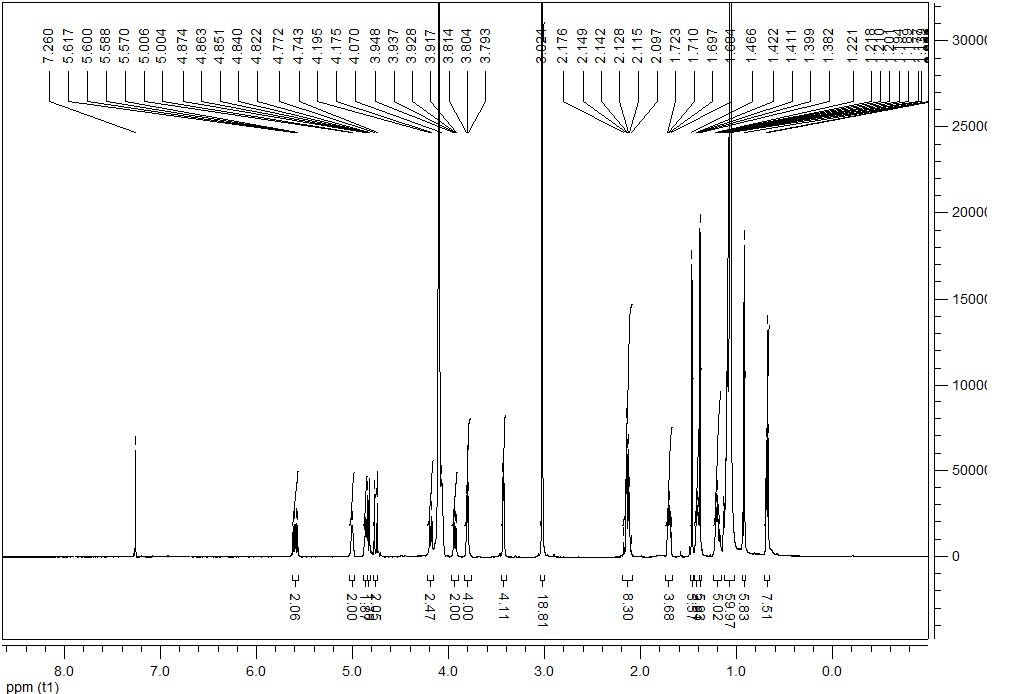

Supplement: S61 Fig — (DOCX) [file pone.0172238.s061.docx]

## S62 Fig. 13C NMR spectrum of 9a


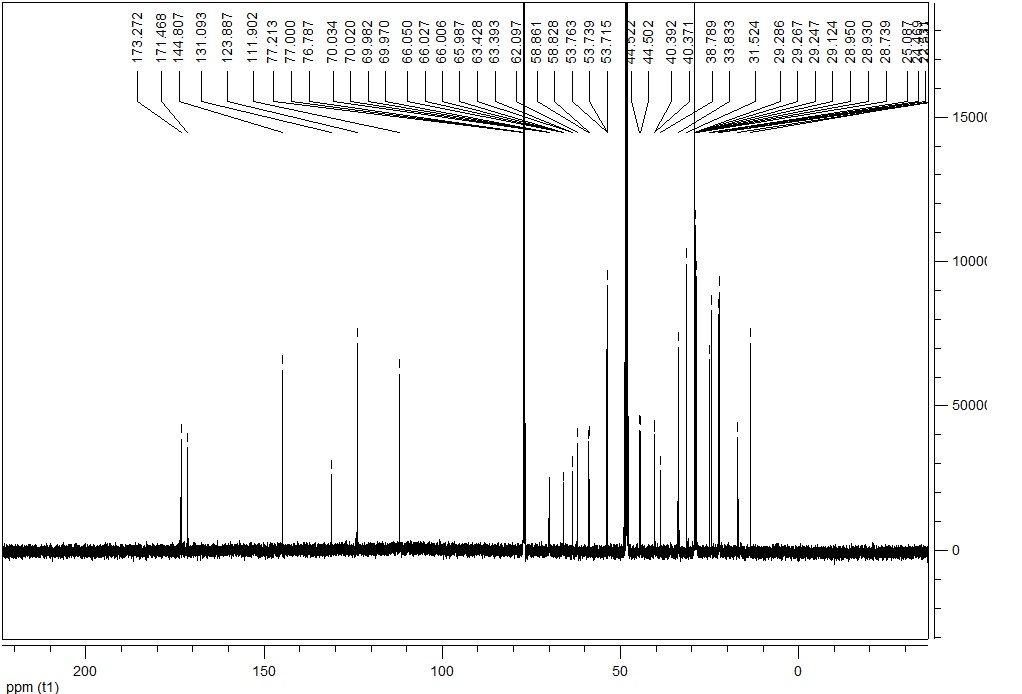

Supplement: S62 Fig — (DOCX) [file pone.0172238.s062.docx]

## S63 Fig. 31PNMR spectrum of 9a

##
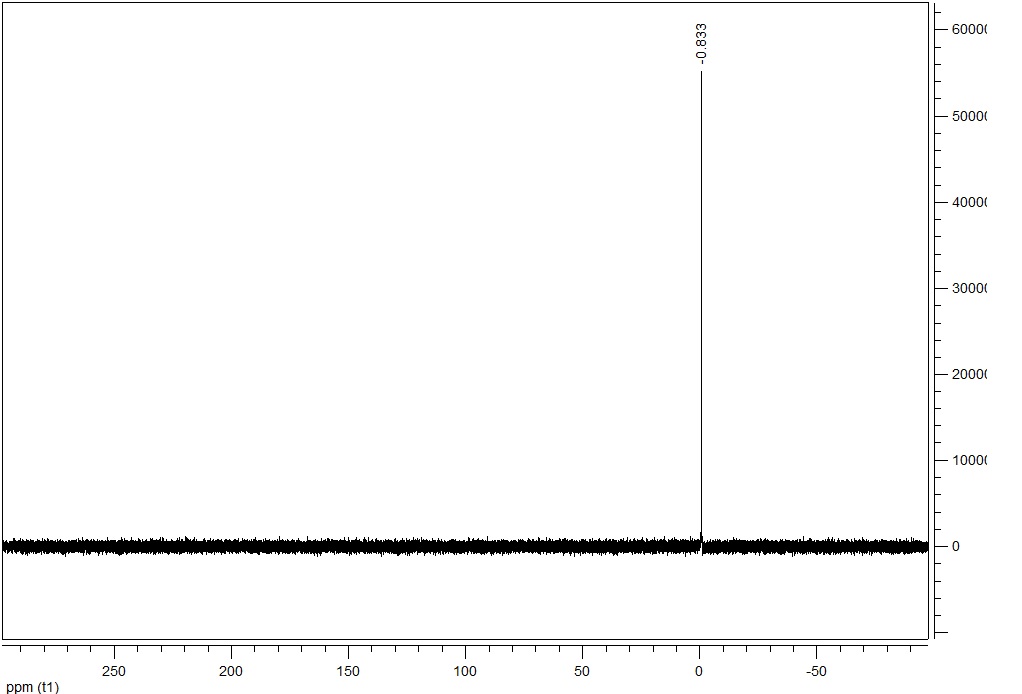

Supplement: S63 Fig — (DOCX) [file pone.0172238.s063.docx]

## S64 Fig. 1H – 1H COSY spectrum of 9a

**
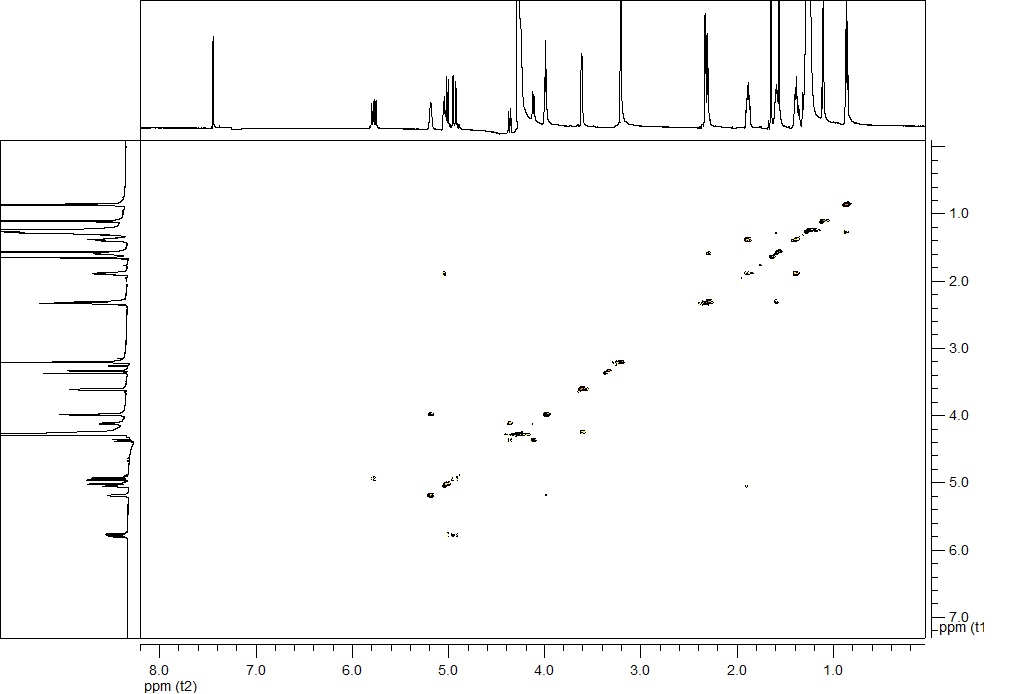
**

Supplement: S64 Fig — (DOCX) [file pone.0172238.s064.docx]

## S65 Fig. HSQC spectrum of 9a

**
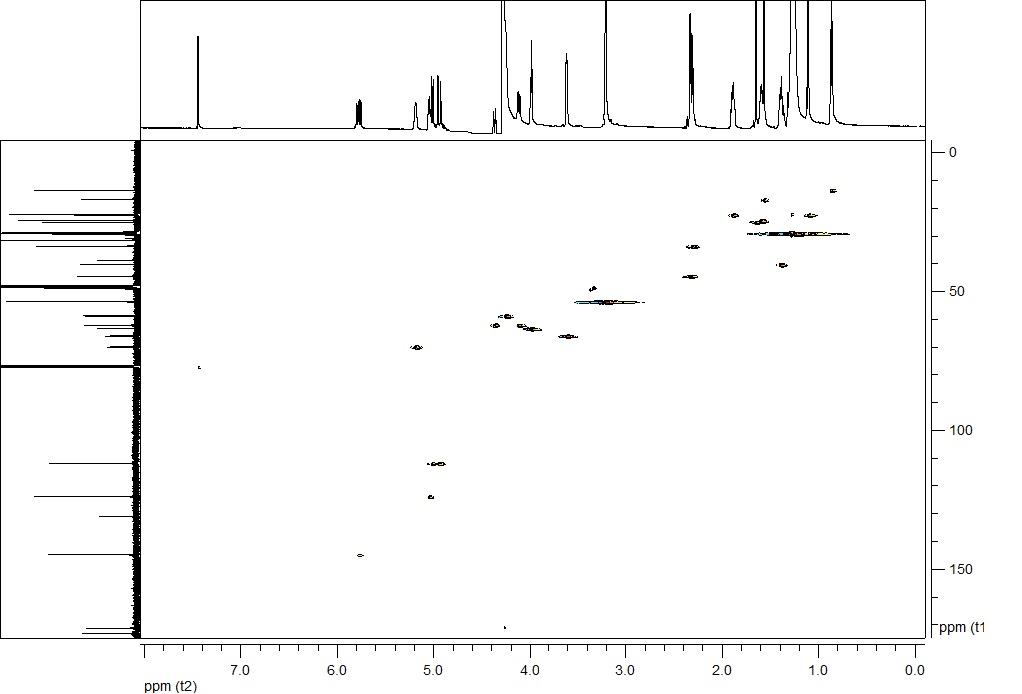
**

Supplement: S65 Fig — (DOCX) [file pone.0172238.s065.docx]

## S66 Fig. 1H NMR spectrum of 9b


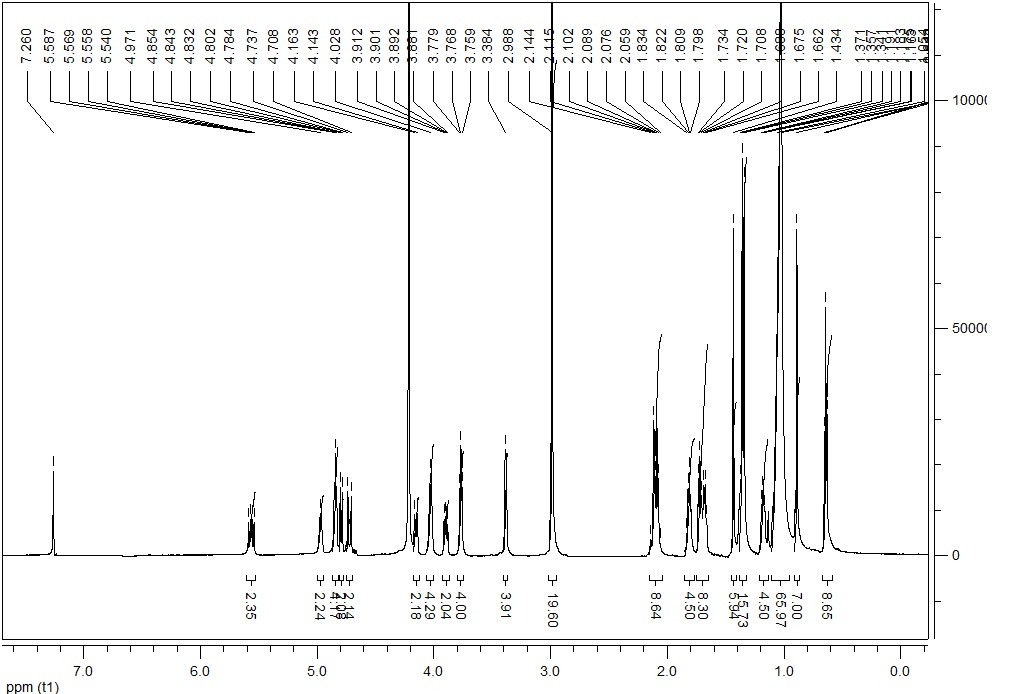

Supplement: S66 Fig — (DOCX) [file pone.0172238.s066.docx]

## S67 Fig. 13C NMR spectrum of 9b


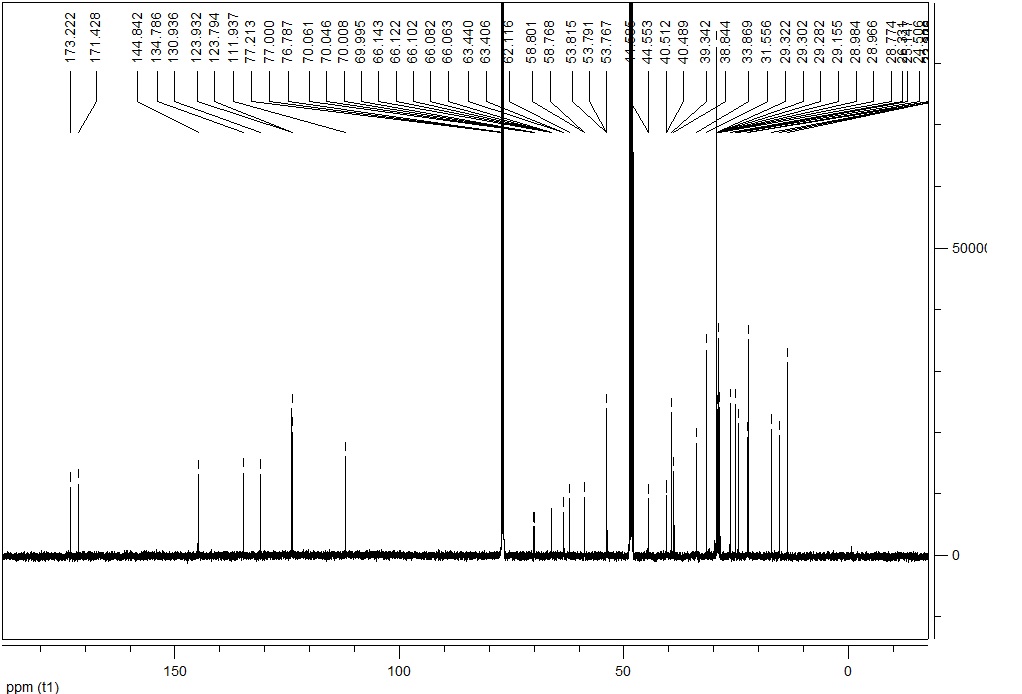

Supplement: S67 Fig — (DOCX) [file pone.0172238.s067.docx]

## S68 Fig. 31PNMR spectrum of 9b

**
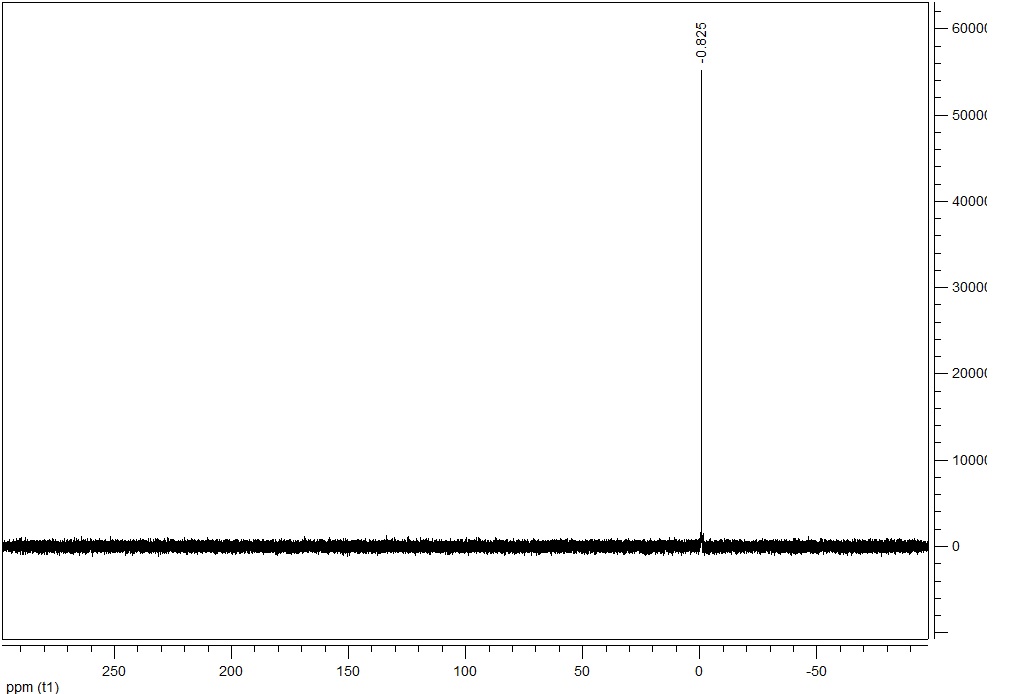
**

Supplement: S68 Fig — (DOCX) [file pone.0172238.s068.docx]

## S69 Fig. 1H – 1H COSY spectrum of 9b

**
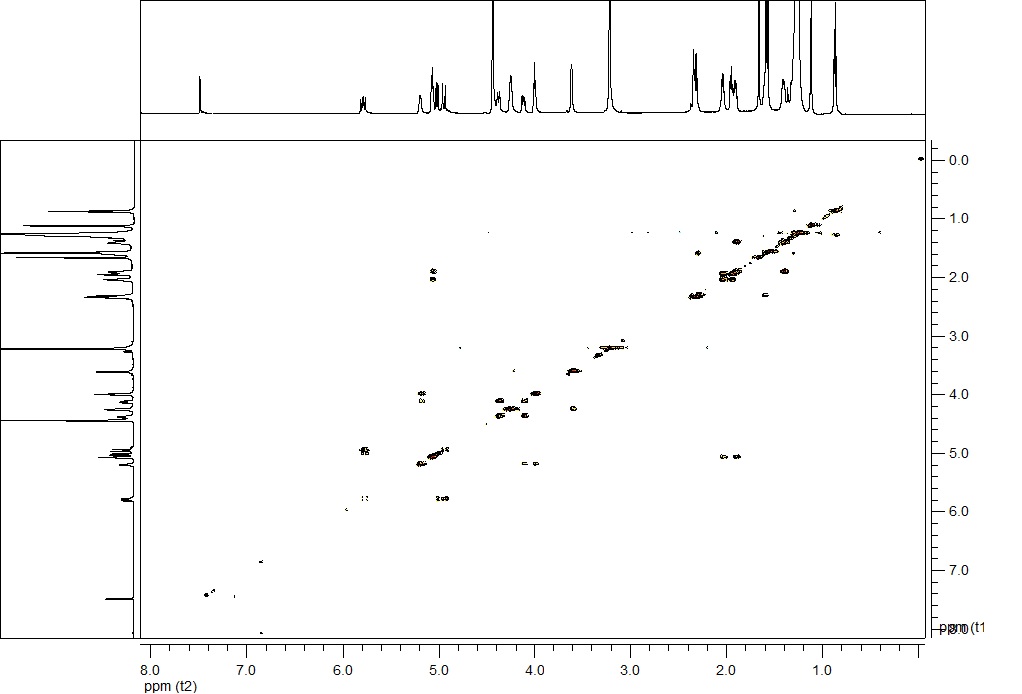
**

Supplement: S69 Fig — (DOCX) [file pone.0172238.s069.docx]

## S70 Fig. HSQC spectrum of 9b

**
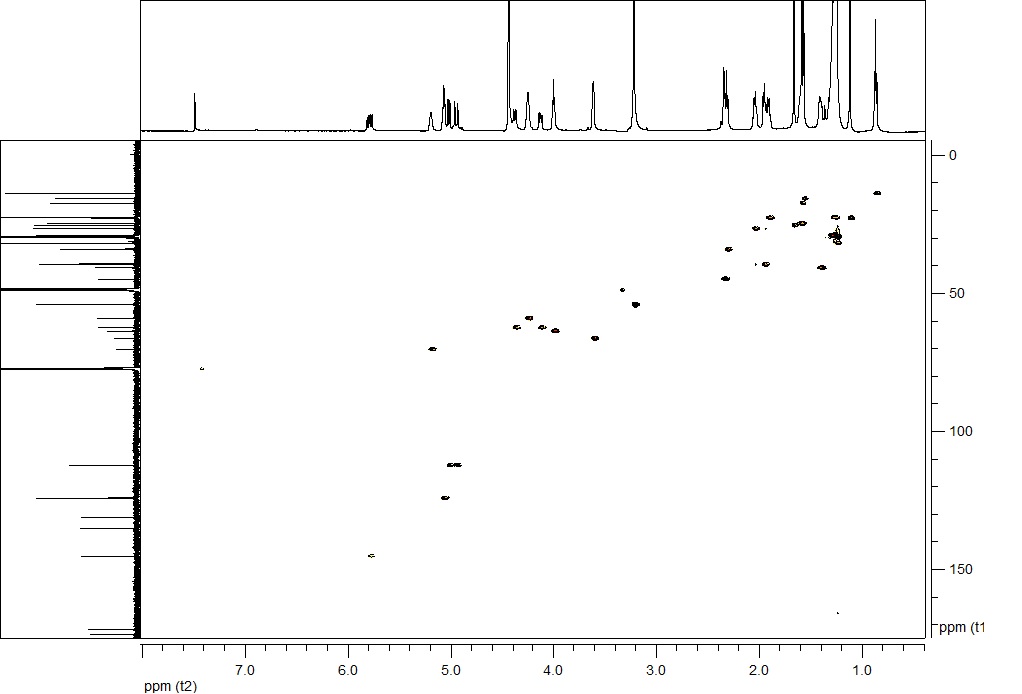
**

Supplement: S70 Fig — (DOCX) [file pone.0172238.s070.docx]

## S71 Fig. 1H NMR spectrum of 9c


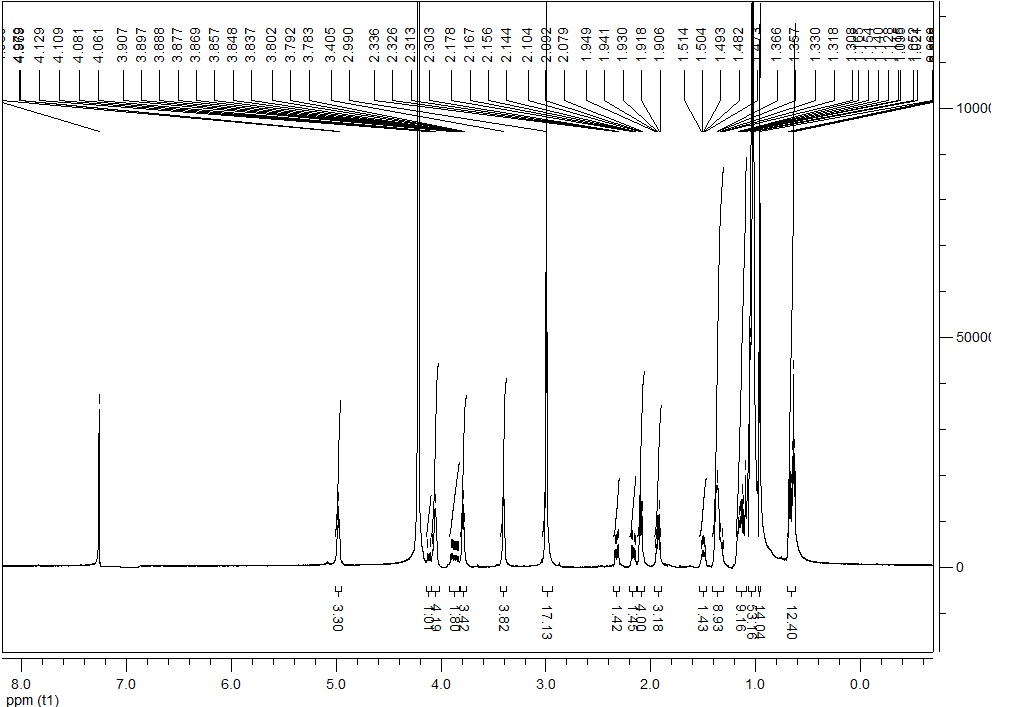

Supplement: S71 Fig — (DOCX) [file pone.0172238.s071.docx]

## S72 Fig. 13C NMR spectrum of 9c


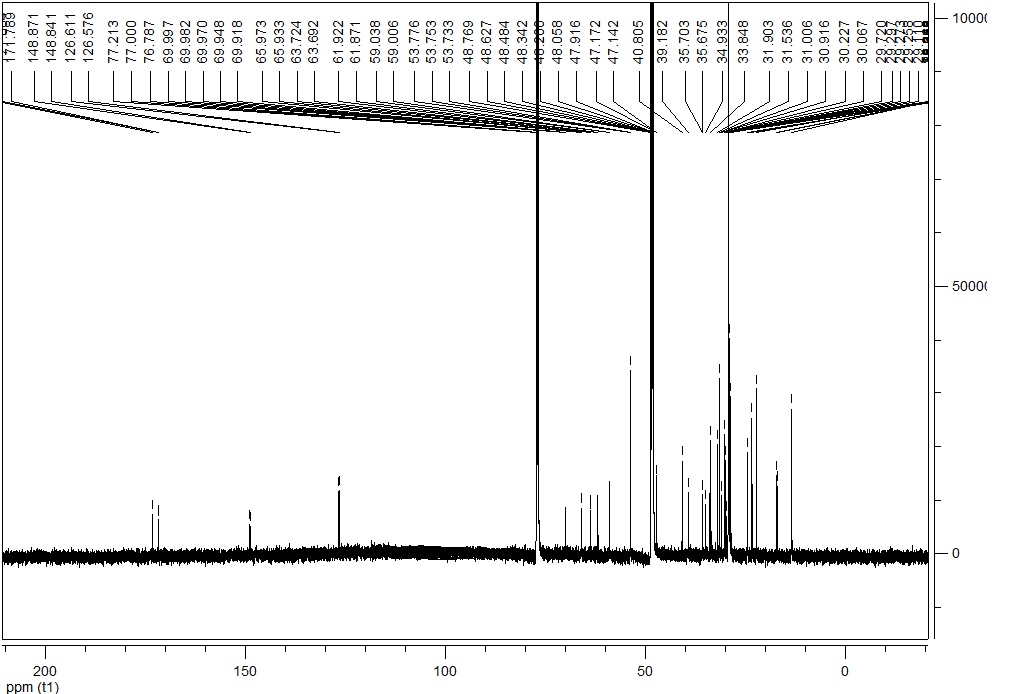

Supplement: S72 Fig — (DOCX) [file pone.0172238.s072.docx]

## S73 Fig. 31PNMR spectrum of 9c

##
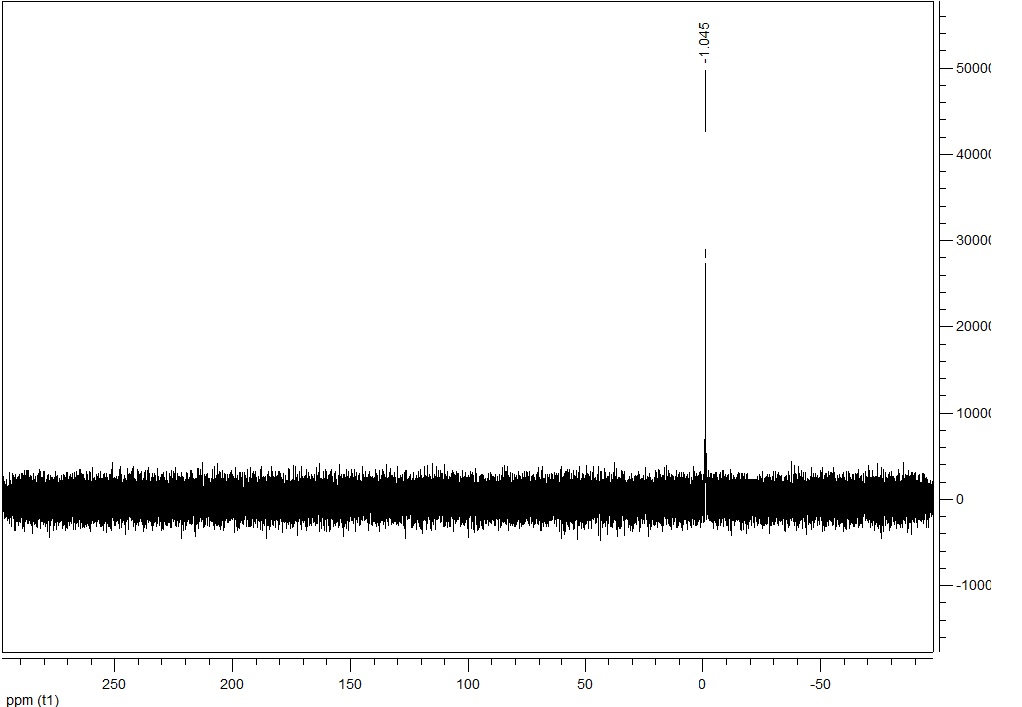

Supplement: S73 Fig — (DOCX) [file pone.0172238.s073.docx]

## S74 Fig. 1H – 1H COSY spectrum of 9c

**
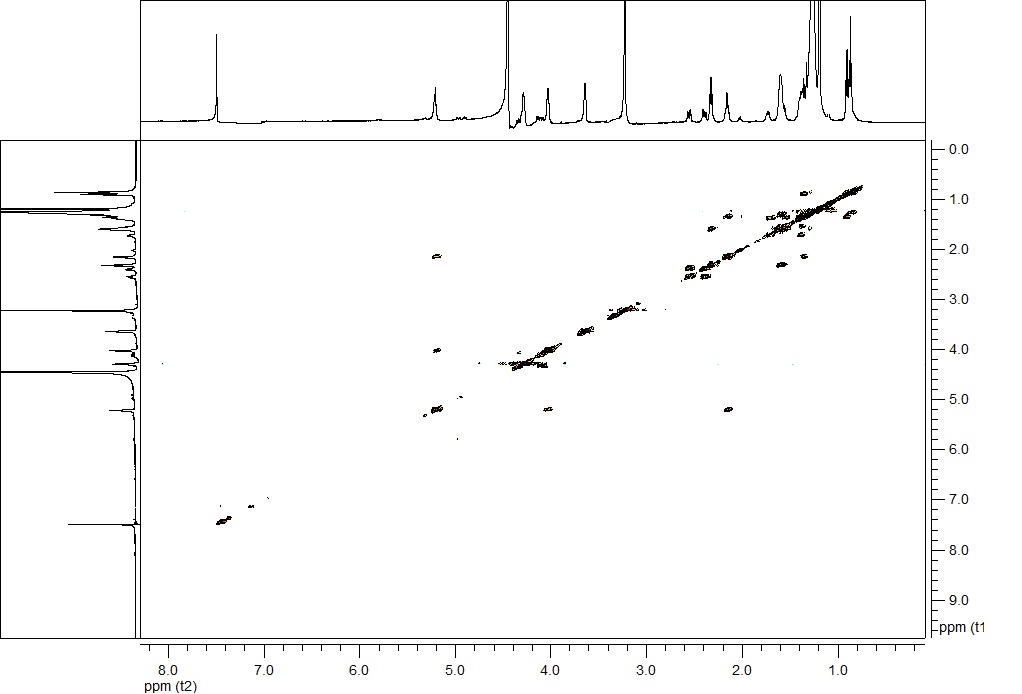
**

Supplement: S74 Fig — (DOCX) [file pone.0172238.s074.docx]

## S75 Fig. HSQC spectrum of 9c

**
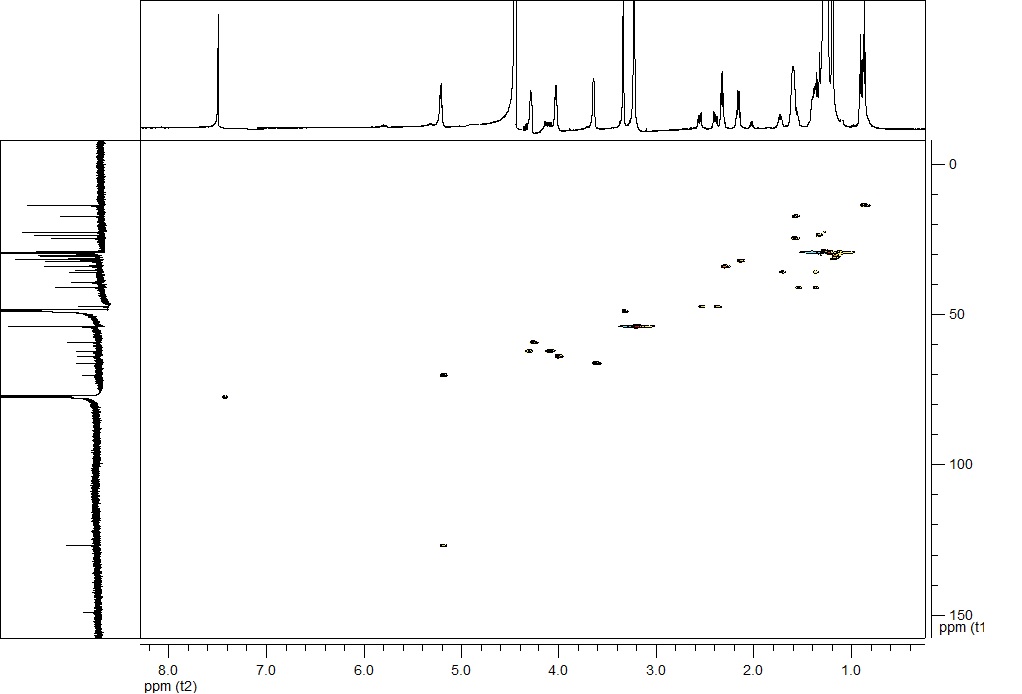
**

Supplement: S75 Fig — (DOCX) [file pone.0172238.s075.docx]

## S76 Fig. 1H NMR spectrum of 9d


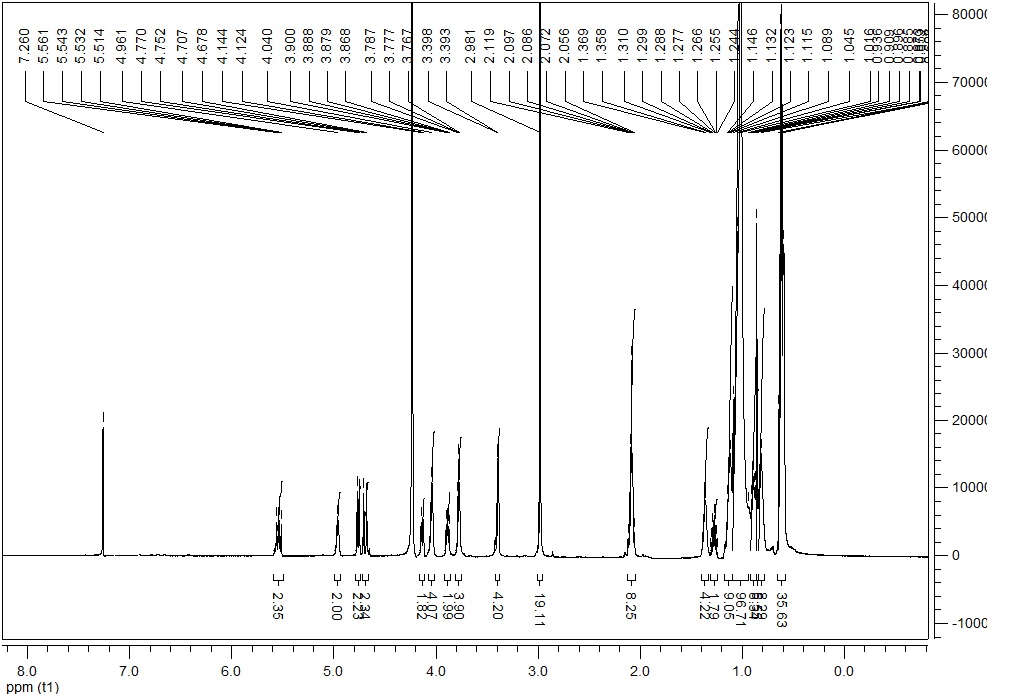

Supplement: S76 Fig — (DOCX) [file pone.0172238.s076.docx]

## S77 Fig. 13C NMR spectrum of 9d


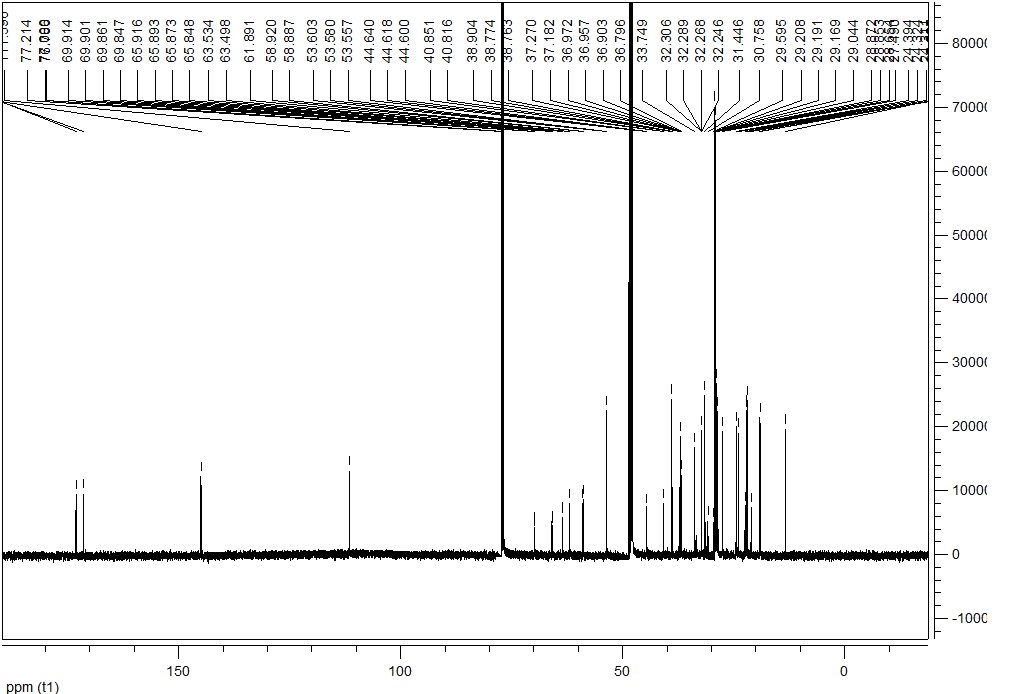

Supplement: S77 Fig — (DOCX) [file pone.0172238.s077.docx]

## S78 Fig. 31PNMR spectrum of 9d

**
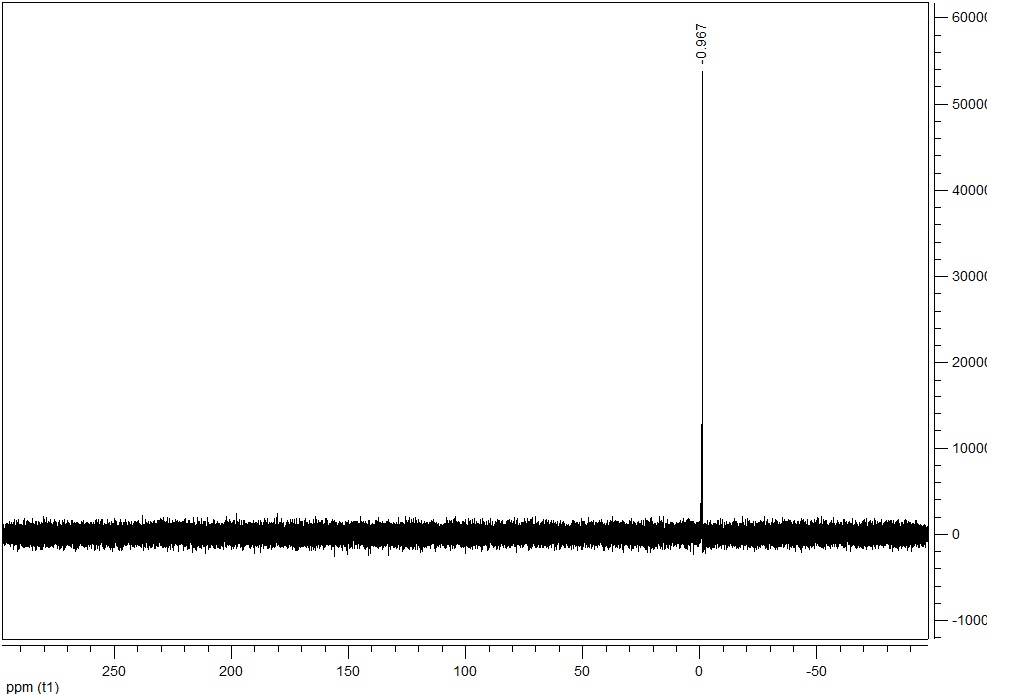
**

Supplement: S78 Fig — (DOCX) [file pone.0172238.s078.docx]

## S79 Fig. 1H – 1H COSY spectrum of 9d

**
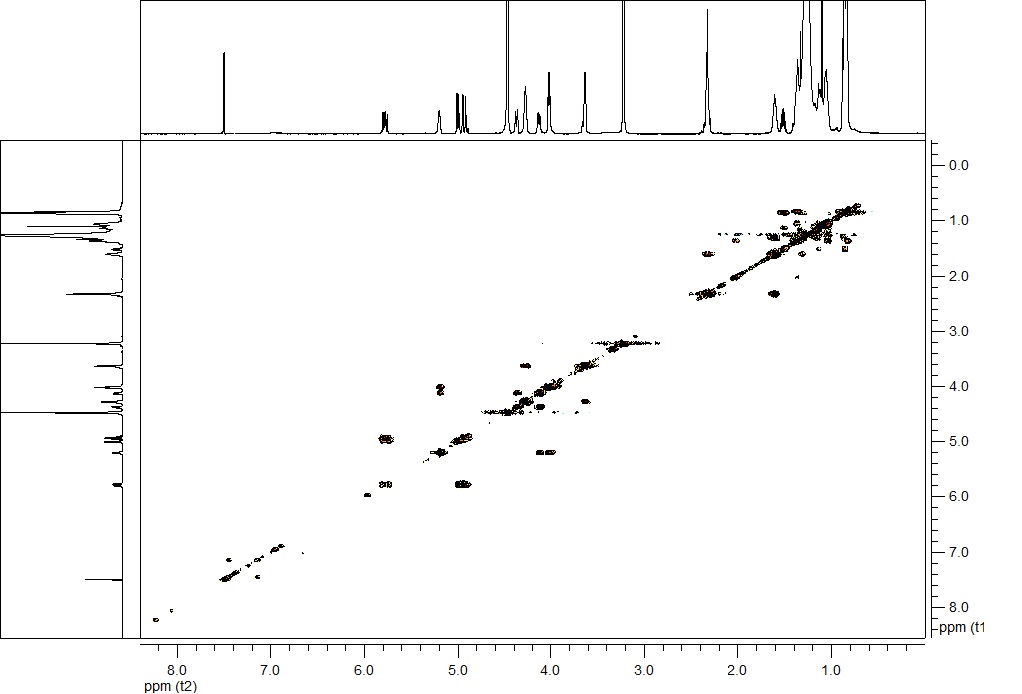
**

Supplement: S79 Fig — (DOCX) [file pone.0172238.s079.docx]

## S80 Fig. HSQC spectrum of 9d

**
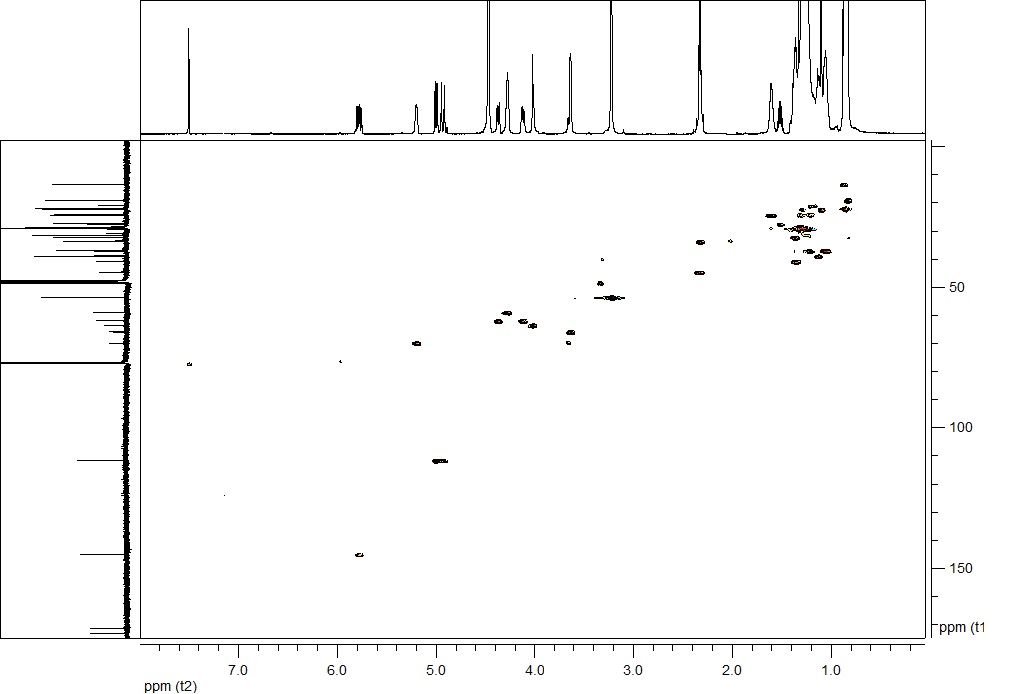
**

Supplement: S80 Fig — (DOCX) [file pone.0172238.s080.docx]
