# Supplementary material for: Isoprenoid-phospholipid conjugates as potential therapeutic agents: Synthesis, characterization and antiproliferative studies
Source: PLoS One. 2017 Feb 14;12(2):e0172238. doi: 10.1371/journal.pone.0172238 (PMC5308787; doi:10.1371/journal.pone.0172238)
Supplement: S58 Fig — (DOCX) [file pone.0172238.s058.docx]

## S58 Fig. ^31^P NMR spectrum of 8d
